# Brain micro-architecture and disinhibition: a latent phenotyping study across 33 impulsive and compulsive behaviours

**DOI:** 10.1038/s41386-020-00848-9

**Published:** 2020-09-12

**Authors:** Rafa Romero-Garcia, Roxanne W. Hook, Jeggan Tiego, Richard A. I. Bethlehem, Ian M. Goodyer, Peter B. Jones, Ray Dolan, Jon E. Grant, Edward T. Bullmore, Murat Yücel, Samuel R. Chamberlain

**Affiliations:** 1 Department of Psychiatry, University of Cambridge, and Cambridgeshire and Peterborough NHS Foundation Trust, Cambridge, UK; 2 School of Psychological Sciences and Turner Institute for Brain and Mental Health, Monash University, c/o MBI, 770 Blackburn Rd, Clayton, VIC 3800 Australia; 3 Max Planck University College London Centre for Computational Psychiatry and Ageing Research, London, UK; 4 Wellcome Centre for Human Neuroimaging, University College London, London, UK; 5 Department of Psychiatry & Behavioural Neuroscience, University of Chicago, Chicago, IL USA; 6 Department of Psychiatry, University of Southampton; and Southern Health NHS Foundation Trust, Southampton, UK

**Keywords:** Signs and symptoms, Neuroscience

## Abstract

Impulsive and compulsive symptoms are common, tend to co-occur, and collectively account for a substantive global disease burden. Latent phenotyping offers a promising approach to elucidate common neural mechanisms conferring vulnerability to such symptoms in the general population. We utilised the Neuroscience in Psychiatry Network (NSPN), a cohort of young people (aged 18–29 years) in the United Kingdom, who provided questionnaire data and Magnetic Resonance Imaging scans. Partial Least Squares was used to identify brain regions in which intra-cortical myelination (measured using Magnetisation Transfer, MT) was significantly associated with a disinhibition phenotype, derived from bi-factor modelling of 33 impulsive and compulsive problem behaviours. The neuroimaging sample comprised 126 participants, mean 22.8 (2.7 SD) years old, being 61.1% female. Disinhibition scores were significantly and positively associated with higher MT in the bilateral frontal and parietal lobes. 1279 genes associated with disinhibition-related brain regions were identified, which were significantly enriched for functional biological interactions reflecting receptor signalling pathways. This study indicates common microstructural brain abnormalities contributing to a multitude of related, prevalent, problem behaviours characterised by disinhibition. Such a latent phenotyping approach provides insights into common neurobiological pathways, which may help to improve disease models and treatment approaches. Now that this latent phenotyping model has been validated in a general population sample, it can be extended into patient settings.

## Introduction

The impulsivity-compulsivity diathesis has been fruitful for examining a range of psychiatric disorders that are commonplace globally, as well as day-to-day behaviours. Impulsivity refers to behaviours that are inappropriate, risky, unduly hasty, and that lead to untoward outcomes [1]. By contrast, compulsivity refers to repetitive, perseverative actions that are excessive and inappropriate to a given situation [2]. For example, an individual with attention-deficit hyperactivity disorder (ADHD) may manifest impulsive problems such as making a statement they regret to a colleague; or jumping a red light; whereas an individual with obsessive-compulsive disorder (OCD) may repeatedly (i.e. compulsively) check the front door is locked, for hours per occasion. Collectively, such symptoms lead to considerable functional impairment and burden of disease [3–6]. It was traditionally thought that impulsivity and compulsivity were diametrically opposed concepts, and indeed current nosological systems often place these disorders in disparate categories. However, impulsive and compulsive problems frequently co-occur in the same individual, and some types of disorders, such as addictions, may shift from being impulsive to compulsive over time [7], suggesting that in fact both types of symptom are biologically related.

It has been proposed that psychiatric symptoms may be driven by common mediators (termed ‘latent phenotypes’) that cut across conventionally discrete nosological boundaries [8, 9]. Such latent phenotypes are expected to exist in a dimensional or continuous fashion in the general population, confirmation of which can be seen as a precursor to using such models in clinical settings. Understanding of such dimensional phenotypes and their biological substrates is highly relevant to understanding the normal range of human behaviour, as well as prevalent mental disorders.

By collecting data regarding 33 types of impulsive and compulsive behaviours in a population sample, it was demonstrated that 70% of expression of these symptoms, within an optimal statistical model, was explained by a latent phenotype termed ‘disinhibition’ [10]. Conceptually, disinhibition—i.e. a loss of top down control governing behaviour—has been extensively implicated as a mechanism contributing to impulsive and compulsive disorders (such as ADHD and OCD), viewed individually (i.e. per disorder) in prior literature [11–14]. The frontal cortices work synergistically with other brain regions to enable top-down control over behaviours [15], and frontal architectural abnormalities have been reported in impulsive and compulsive disorders [16–24]. The latent phenotyping approach assumes that similar mechanisms (such as disinhibition) operate both in normative population samples, and in groups of people with significant psychopathology; and that it is the extent of latent phenotype (rather than its nature) that accounts for why some people exhibit psychiatric symptoms meeting threshold for a diagnosis, and others do not. The continuity of latent phenotypes has been exemplified in other areas of mental health research, notably in the context of psychosis [25] and general psychopathology [26]. Here, we consider common neurobiological mechanisms that may confer vulnerability for both impulsive and compulsive symptoms, considered dimensionally in a sample of young adults.

Myelinated fibres are extensively distributed within the cortex [27–29], and play a key role in neural plasticity and communication between cortical regions [30]. Intra-cortical myelin content is inversely related to neural circuit complexity: typically, higher myelination is found in early sensorimotor cortical regions, while lower myelination is evident in regions involved in complex higher-level cognitive processes, notably in the frontal lobes [31]. Nevertheless, high-level associative cortices get not only thinner during adolescence but also more myelinated, which could be driven by a genetically patterned process of consolidation of cortical regions that are more densely connected [32]. Previous studies have extensively documented reduced cortical thickness in disorders such as OCD [16] and ADHD [33]. Intra-cortical myelin content can be readily quantified using Magnetisation Transfer (MT) acquired using brain imaging [31], since MT exhibits strong positive correlations with myelination in histological brain samples [34, 35]. Frontal cortex MT was previously found to be abnormally elevated in OCD patients compared to controls [36]. MT is sensitive to neurodevelopmental changes in the brain [37], including longitudinal changes associated with impulsive traits and OC symptoms [38], and is a promising measure of brain architecture that can be related to dimensional phenotypes [39].

Given this explanatory power of the latent disinhibition phenotype, coupled with individual differences in myelin-related brain growth during early adulthood [40], understanding the neural mechanisms underpinning this novel latent phenotype is an important next step. Therefore, the primary aim of the current study was to identify relationships between intra-cortical myelination (quantified using MT) and the expression of a latent phenotype of disinhibition [10]; i.e. disinhibition-related myelination. Our premise was that disinhibition arises from alterations in frontal brain architecture, manifesting as increased intra-cortical white matter (MT) and concomitant reductions in cortical thickness. In view of the centrality of certain neurochemical systems in understanding impulsivity and compulsivity [41, 42], along with recent methodological developments [40, 43], the secondary aim of this study was to inferentially ascertain genes co-localised with disinhibition-related cortical regions, by cross-referencing against a public-domain brain atlas [44]. We hypothesised (i) that disinhibition would be associated with elevated MT (and concomitant reductions in grey matter cortical thickness), in frontal and other cortical regions; and (ii) that genes significantly associated with disinhibition-related regions would be identified, which would be inferentially enriched for functional interactions involving receptor signalling pathways implicated in impulsivity/compulsivity.

## Materials and methods

### Study design

**Fig. 1:**
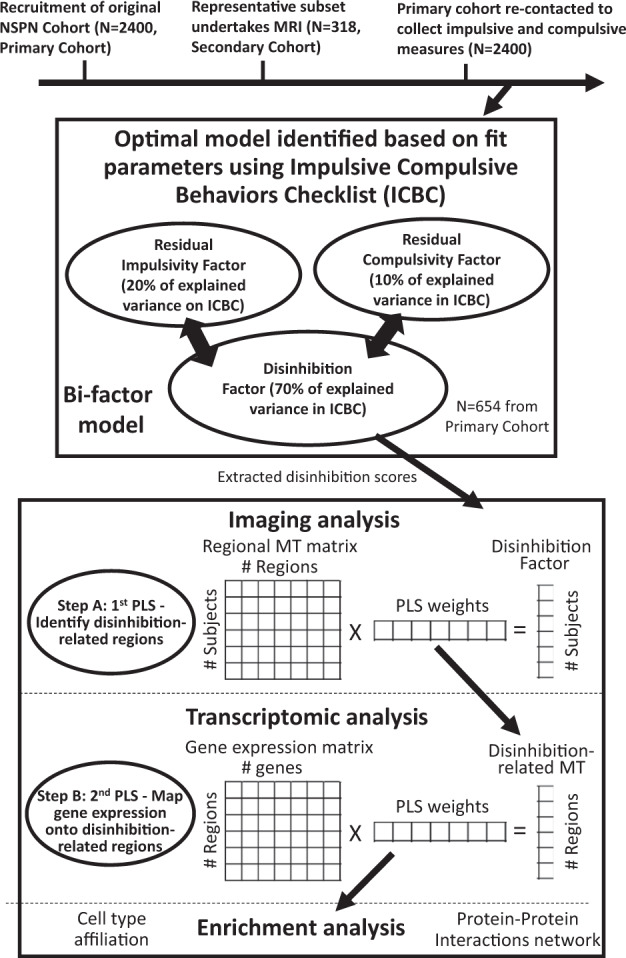
Overview of study design. Disinhibition scores for each subject were extracted using a previously validated optimal bi-factor model. We first (Part A) used Partial Least Squares (PLS) regression to map intra-cortical myelination to those disinhibition scores. We then (Part B) used PLS to identify genes inferentially over-expressed in those disinhibition-related brain regions.

An overview of the study design is provided in Fig. 1. Participants were recruited from a cohort of young people being followed over time to evaluate human development (the Neuroscience in Psychiatry Network, NSPN) [45]. The original NSPN cohort (primary cohort) comprised participants aged 14–25 years at enrolment, who were assessed by completion of psychopathology questionnaires. Subjects were recruited in five contiguous age-related strata, each balanced for sex and ethnicity. Exclusion criteria were a current or past history of clinical treatment for a psychiatric disorder, drug or alcohol dependence, neurological disorder including epilepsy, head injury causing loss of consciousness, or learning disability.

NSPN constitutes what can be considered a normative cohort, but not exclusively ‘healthy controls’. This makes the cohort extremely useful for exploring candidate latent phenotype markers, since problem behaviours will occur along continua, from no problems to many problems. A secondary cohort of these participants additionally completed a later in-unit assessment comprising magnetic resonance imaging (MRI). The secondary cohort was demographically balanced, and was sub-sampled from the primary cohort [43, 45]. Contraindication to MRI was exclusionary for the secondary cohort. We subsequently collected information regarding a comprehensive range of impulsive and compulsive problems by re-contacting the primary cohort (see Fig. 1), as described in more detail below.

### Ethics

All participants provided written informed consent and this research was approved by Research Ethics Committee (East of England—Cambridge East Research Ethics Committee).

### Bi-factor modelling of impulsive and compulsive symptoms (primary cohort)

Information on impulsive and compulsive symptoms was collected from 654 NSPN participants, who completed the Impulsive-Compulsive Behaviours Checklist (ICBC) [10]. This was achieved by contacting all individuals from the primary cohort via email, and inviting them to complete a follow-up questionnaire comprising more detailed information about impulsive and compulsive problems. This subset of the NSPN cohort who provided ICBC data were representative of the original cohort in terms of age at enrolment, gender, and ethnicity (Table S1). The ICBC [46] quantified 33 impulsive and compulsive symptoms; for example, it includes impulse control problems (gambling, substance use, aggression, etc.) and compulsive problems (e.g. washing, checking, making lists, counting, etc.) (Table S2). In prior analysis of structural models capable of explaining ICBC responses, the optimal model (according to fit parameters) was the bi-factor model shown in Fig. 1. The bi-factor model has superior model fit to the approach of using summary scores, across the full range of fit parameters (Comparative Fit Index, CFI; Root Mean Square Error of Approximation, RMSEA; Weighted Room Mean Square Residual, WRMR; and chi-square test) [10]. As such, the bi-factor approach is clinically preferred, rather than using (for example) the numerical sum of scores from the instrument, because the latter results in marked loss of information content. This bi-factor model yields the latent phenotype of disinhibition, which accounts for ~70% of explained variance in the expression of these 33 ICBC symptoms [10]. This disinhibition factor has some conceptual similarities with the “p factor” [47], but differs in that it applies specifically to impulsive and compulsive problems, rather than to other forms of psychopathology. This disinhibition model is also supported by complementary lines of evidence using other instruments, in normative and mixed clinical and non-clinical samples, including participants diagnosed with Gambling Disorder and OCD (i.e. archetypal impulsive and compulsive disorders) [48, 49]. To contextualise the levels of archetypal forms of impulsivity and compulsivity in the sample, participants also completed self-report questionnaires for ADHD [Adult ADHD Self-Report Screening Scale [50]], and OCD [Padua Obsessive-Compulsive Inventory (Washington State Revision) [51]]. In particular, it was intended to correlate latent disinhibition scores against these archetypal impulsive and compulsive symptoms, as measured using independent instruments, to support the validity of the model. Total scores from the Adult ADHD Self-Report Screening Scale and Padua Inventory were used for this purpose; we additionally examined sum of scores for impulsive items and inattentive items from the ADHD Scale separately, the expectation being that disinhibition would relate to impulsive items but not inattentive items.

Normalised and standardised disinhibition factor score estimates (hereafter referred to as ‘disinhibition scores’) were calculated using the regression method based on the modelling of *N* = 654 participants for whom complete ICBC data were available. We confirmed that the distribution of disinhibition scores for the imaging sample did not differ from those of non-imaging sample (imaging, mean [SD], 0.07 [0.84]; non-imaging, 0.06 [0.81]; *F* = 0.0297, *p* = 0.863).

For the 126 participants for whom MRI data were also available, we explored the disinhibition-related relationships between cortical MT measurements in 308 cortical areas, and the disinhibition factor score estimates, as described below. MRI data were not collected from everyone in the original NSPN study but rather from a random representative subset.

### MRI measurement of intra-cortical magnetisation transfer (secondary cohort)

Magnetic resonance imaging was undertaken using identical 3T MRI systems (Magnetom TIM Trio; VB17 software version; Siemens Healthcare) operating with the standard 32-channel radio-frequency (RF) receive head coil and RF body coil for transmission and located at the Wolfson Brain Imaging Centre, University of Cambridge; the Medical Research Council (MRC) Cognition & Brain Sciences Unit, University of Cambridge; or the Wellcome Trust Functional Imaging Laboratory (FIL) at University College London. Multi-parametric mapping (MPM) sequences were used to collect data on several microstructural parameters in a single scan, with satisfactory between-site reliability of measurement across all sites in a prior pilot study, e.g., the percentage (standard deviation) of between-site coefficient of variation for Magnetisation Transfer (MT) was 7.8 ± 0.8, 7.6 ± 2.7, 6.1 ± 0.6 and 7.4 ± 2.8 for grey matter, caudate nucleus, white matter and corpus callosum, respectively [52]. MPM comprises 3 multi-echo fast low angle shot (FLASH) scans with variable excitation flip angles. Multiple gradient echoes were acquired with alternating readout polarity at six equidistant echo times (TE) between 2.2 and 14.7 ms for the T1 weighted and MT weighted acquisitions and at 8 equidistant MT was quantified by appropriate choice of repetition time (TR) and flip angle (TR = 23.7 ms, α = 6°). Other acquisition parameters were: 1 mm^3^ voxel resolution, 176 sagittal slices and field of view (FOV) = 256 × 240 mm.

Pre-processing of MRI data was undertaken using Freesurfer pipelines [53], version 5.3.0. In brief, each image was subjected to skull stripping, segmentation, and reconstruction of the pial surface [54–56]. The Desikan-Killany atlas of 68 regions implemented in Freesurfer was subdivided into 308 contiguous parcels of approximately equal area of 500 mm^2^ using a subparcellation algorithm described in [57]. Increasing the resolution of the atlas allow us to define homogeneous parcels where regions represent the same proportion of the cortex and have similar SNR (i.e. average regional MT values are computed for each region across approximately the same number of voxels). The subdivided (308) parcellation was transformed from standard fsaverage space into the native space of each individual using surface-based corregistration to minimise geometric distortions and age-related biases [40, 57].

At each regional node, magnetisation transfer (MT) was estimated intra-cortically at 70% cortical depth, where pial surface was 0% depth and grey-white boundary was 100% depth [32]. We also extracted cortical thickness at each regional node, according to standard methodology [32].

Our overall imaging analytic approach used two distinct steps. First (Part A), we tested the relationship between intra-cortical myelination (MT) and disinhibition factor scores. Second (Part B), we used a gene expression matrix to identify genes overexpressed in the disinhibition-related brain regions identified from Part A (see Fig. 1 for illustration).

### Part A: analysis of relationships between cortical MT and disinhibition scores

We used the statistical technique of Partial Least Squares Regression (hereafter referred to as PLS) to identify relationships between MT and disinhibition. PLS is a multivariate statistical technique for modelling relationships between predictor and response variables, by fitting one or more components [58–60]. Unlike conventional statistical approaches (such as standard regression), PLS is suitable for use when variables are likely to be inter-correlated, and non-normal; and in datasets with relatively large numbers of variables relative to the sample size [61].

The first PLS analysis used many predictors (308 brain regions) to identify a combination of brain regions related to one outcome (126 disinhibition scores). The predictor variables comprised a matrix of 126 rows (participants) by 308 columns (intra-cortical MT measurements in each brain parcel). The response variable was a vector of length 126 (disinhibition scores). We fitted PLS models using leave-one-out (LOO) cross-validation (non-linear iterative partial least squares, NIPALS algorithm), and the optimal model was identified based on minimising predictive residual sum of the squares (PRESS). LOO cross-validation is preferred in situations involving relatively large numbers of variables [62]. From the initial model, measures with a Variable Importance Parameter (VIP) < 0.8 were excluded per standard PLS recommendations [63]. We then used bootstrapping [resampling with replacement [64] with 2500 iterations] to confirm whether the 95% confidence intervals for the amount of variance explained in the predictive and response variables, for this model, was significantly higher than those accounted for by a randomly permuted model. Individual predictive variables significantly contributing to the model (i.e. cortical MT in the parcelled brain regions explaining variance in disinhibition scores) were identified on the basis of 95% confidence intervals of the standardised model coefficients for the given predictor variable, again obtained using bootstrap (2500 iterations), did not cross the null line (i.e. zero). To identify normative psychological processes linked to the model coefficient of disinhibition-related brain regions, we used the Neurosynth tool (http://neurosynth.org) [65], which comprises a large pooled database of functional neuroimaging studies.

Correlations were undertaken between PLS scores and total scores from: the Adult ADHD Self-Report Screening Scale [50], and the Padua Obsessive-Compulsive Inventory (Washington State Revision) [66]. These two instruments measured archetypal impulsive and compulsive symptoms respectively; and were not used in the construction of the PLS model, nor in the calculation of disinhibition scores. Hence these correlations were undertaken to affirm that the identified brain regions were also related to these archetypal symptom types, as would be expected for a disinhibition phenotype. We checked that the findings were not confounded by alcohol use by correlating against total scores from the well-validated FAST alcohol use disorder tool [67].

### Part B: inferential mapping of brain genes whose expression related to disinhibition-relevant brain regions

To identify genes whose expression was inferentially correlated with the disinhibition-related brain regions, we used a second, separate PLS model. This second PLS analysis used many predictors (maps of brain gene expression) to identify genes inferentially over-expressed in disinhibition-related brain regions. The predictor variables constituted a gene expression matrix of 308 rows (i.e. 308 brain parcels) by 20647 columns (i.e. 20647 genes). The response variable constituted a vector of 308 values, being the matrix of disinhibition-related MT obtained in Part A. The micro-array gene expressions were obtained by utilising the Allen Human Brain Atlas database, which is a dataset from six adult donors whose brain expressions in different regions were quantified post mortem (three Caucasian, two African-American and one Hispanic; five males, one female; aged 57, 55, 49, 39, 31 and 24 years; www.brain-map.org) [44]. While this atlas comprises data from few subjects, it currently constitutes the gold standard in the field for cross-referencing against brain gene expression, until future larger studies are conduced across a broader set of subjects. Full details of the methodology for obtaining these gene expressions, and mapping them to cortical parcels are provided in [43]. The first PLS component was extracted, representing the linear combination of the weighted gene expression scores that had a cortical expression map that was most strongly associated with the disinhibition-related brain region map. Permutation testing based on 10,000 spherical rotations or “ spins” of the spatially correlated disinhibition-related myelination map (*P*_*spin*_) was used to test the null hypothesis that PLS explained no more covariance between disinhibition-related myelination and whole-genome expression than expected by chance [68].

Bootstrapping was used to estimate the variability of each gene’s weight on PLS and we tested the null hypothesis of zero weight for each gene with false discovery rate (FDR) of 5%. The STRING tool (https://string-db.org) [69] was then used to test for the presence of significant functional enrichment (i.e. gene-gene interactions) of the significant PLS genes against Gene Ontology Biological Processes, with Benjamini–Hochberg FDR correction (*q* < 0.05). We assigned a cellular affiliation score to each gene in the PLS gene list according to prior criteria for 4 cell types—neuron, astrocyte, microglia, or oligodendroglia [70]. We used a permutation test procedure that randomly reassigned cellular affiliation scores across genes to test the null hypothesis that genes correlated with disinhibition-related myelination have random cell type affiliations.

## Results

The demographic characteristics of the 126 subjects in the imaging analyses are provided in Table 1. The average (Standard Deviation) number of problematic behaviours endorsed per subject to at least a moderate degree was 2.2 (3.6), with a range of 0–17.

**Table 1:** Sample characteristics.

	Mean (Standard Deviation) [range] or *N* [%] *N* = 126
Age, years	22.8 (2.7) [18, 29]
Gender, Female	77 [61.1%]
Ethnic group^a^	
White Caucasian	101 [83.5%]
Mixed/Multiple ethnicity	12 [10.0%]
Asian/Asian British	5 [4.1%]
Other	3 [2.5%]
ADHD total score	8.2 (3.6) [0–20]
Padua Inventory (Obsessive-Compulsive) total scores	18.6 (17.7) [0–85]

^a^Five subjects did not disclose their ethnic group.

### Relationship between cortical MT and disinhibition scores

**Fig. 2:**
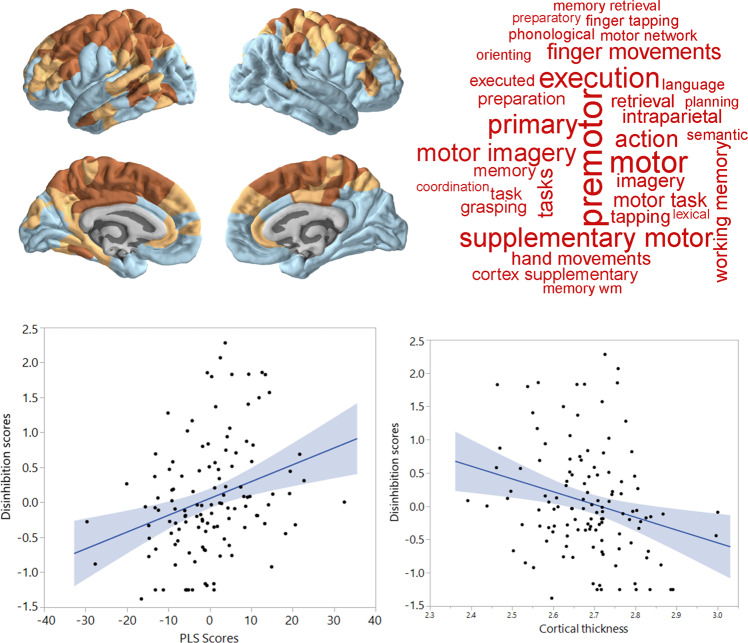
Results of PLS modelling linking Magnetisation Transfer (MT) to the latent disinhibition phenotype. Top left: Glass brain showing disinhibition-related regions in which intra-cortical myelination was significantly associated with disinhibition (dark brown: significant by bootstrap; light brown: variable importance parameter >0.8 but did not withstand bootstrap). Top-right: word cloud showing normative psychological processes linked, in functional imaging literature, to the disinhibition-related brain regions (http://neurosynth.org) [65]. It can be seen that many ontological terms related to motor planning and execution. Bottom left: plot of PLS Scores against disinhibition scores. Bottom right: plot of cortical thickness in those same regions, against disinhibition scores.

PLS revealed an optimal one-factor model, explaining 48% of MT variance in the identified brain regions, and 9.6% of variance in the disinhibition scores. Age, education, and gender were not significant contributors when added to this model, and nor was study site (all VIP < 0.8). The amount of disinhibition variance explained by the model differed significantly from that of the null model (Bootstrap *p* < 0.05). Disinhibition-related myelination cortical regions are shown in Fig. 2. Of 141 cortical parcels in the model, 61 were significant (bootstrap *p* < 0.05). Significant regions in both hemispheres comprised: frontal cortex (inferior, middle, superior, posterior cingulate, paracentral gyrus), and parietal cortex (superior, postcentral gyrus, supramarginal gyrus, precuneus). Additionally, left middle temporal cortex, and left pre-central gyrus, were significant. Higher MT (indicative of lower intra-cortical myelination) was associated with higher disinhibition scores in all the identified significant regions (see Table S3 for the full list of brain parcels and their model coefficients). As expected, disinhibition was also associated with concomitant reductions of cortical thickness in the implicated neural regions (Fig. 2).

PLS brain scores correlated significantly with ADHD symptoms (ADHD Self-Report Screening Scale, rho = 0.294, *p* = 0.001) and with OCD symptoms (Padua Obsessive-Compulsive Inventory, rho = 0.285, *p* = 0.001) (Fig. S1), these rating scales being separate from the instrument used to construct the original disinhibition scores. Additionally, the correlation was specifically significant for the sum of the impulsive items from the ADHD Self-Report Screening Scale (items 5 & 6, *p* = 0.0003) but not for the sum of the inattentive items (items 1–4, *p* = 0.4579). Brain scores were unrelated to alcohol use, as indexed by total scores on the FAST (*p* = 0.182). Distributions of disinhibition scores, and total scores on the ADHD and OCD inventories, are shown in Fig. S2.

In terms of the disinhibition model’s transcriptomic signature, PLS identified an optimal one-factor model that explained 11.3% of variation in brain gene expression and 30.9% of variation in disinhibition-related myelination. The amount of variance explained by the model differed significantly from the null models based on spun parcellation that controlled for regional contiguity and hemispheric symmetry (*P*_*spin*_ = 0.0014). There were 1279 genes significantly weighted on the PLS component (bootstrap, *p* < 0.05, FDR corrected). PLS analyses were repeated using both disinhibition-related myelination and disinhibition-related cortical thickness as response variables in the same model. However, explained variance of the model was reduced to 15.0% (*P*_*spin*_ = 0.022) and the resulting gene weights were extremely similar to the PLS disinhibition-related myelination standalone model (*R*^2^ = 0.95; Fig. S3). For those reasons, the following analyses were restricted to the disinhibition-related myelination model only. Genes weights derived from the disinhibition-related myelination model were well differentiated from gene weights derived from a schizotypy-related model [39] established in a recent study, with low overlap (*R*^2^ = 0.02; Fig. S3).

**Fig. 3:**
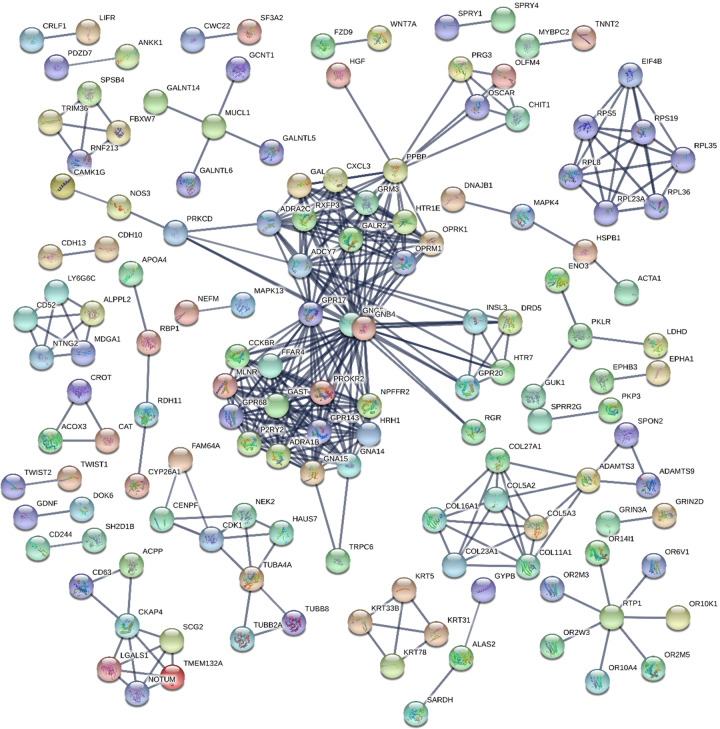
Protein-protein interaction network for top 500 genes whose expression mapped onto the disinhibition phenotype (all genes significant at FDR *p* < 0.001 in the PLS model). Nodes represent genes whose expression was positively associated with disinhibition-related Magnetisation Transfer (MT). Edges (i.e. lines) are known protein-protein interactions, and their weights are proportionate to the STRING confidence score. Only connected notes with high confidence (>0.7) are shown.

Significantly positively weighted genes on the PLS component (Top PLS) were enriched for astrocyte affiliation (permutation test, *P* < 10^−4^) whereas significantly negatively weighted genes (Bottom PLS) were enriched for microglia and oligodendrocyte affiliation (permutation test, *P* < 10^−4^). The top expressed 500 genes in this model are visualised in Fig. 3 using the STRING tool (see Table S4 for full list of genes). The protein-protein interactions that were enriched in the network are summarised in Table 2. It can be seen that the network was significantly enriched for protein-protein interactions relating to a variety of processes including neurochemical transmission (especially G protein-coupled receptor signalling pathways), cellular and biological adhesion, and high-level systems processes.

**Table 2:** Significant functional enrichments for protein-protein interactions in the network of disinhibition-related genes, as extracted using the STRING tool, labelled by (a) biological processes an (b) cellular component.

Term description, biological process	FDR p
Phospholipase C-activating G protein-coupled receptor signalling pathway	0.0017
Response to stimulus	0.007
Adenylate cyclase-modulating G protein-coupled receptor signalling pathway	0.0367
Biological adhesion	0.0367
Multicellular organismal process	0.0367
System process	0.0452
Cell adhesion	0.0452
G protein-coupled receptor signalling pathway	0.0452
Cellular response to stimulus	0.0452
Sensory organ morphogenesis	0.0452
Neuropeptide receptor activity	0.0177
G protein-coupled receptor activity	0.0272
G protein-coupled peptide receptor activity	0.0272
Transmembrane signalling receptor activity	0.031

## Discussion

This study identified micro-structural brain changes associated with an innovative latent phenotype of disinhibition, contributing to 33 impulsive and compulsive problems, in young adults. In keeping with our hypothesis, we found that the latent disinhibition phenotype was associated with higher Magnetisation Transfer (MT), indicative of higher intra-cortical myelination, in bilateral frontal and parietal cortices. There were concomitant reductions of cortical thickness in these regions, as predicted. These results are in accordance with the premise that disinhibition may arise from micro-architectural brain changes impeding the ability of the cortex to exert sufficient control over impulsive and compulsive tendencies. By cross-referencing against a gene expression human brain atlas, we also inferred a transcriptomic profile related to the disinhibition-myelination association; i.e. a network of interacting genes that were co-localised with the disinhibition-related regions. The set of genes differentiated well from those previously implicated in a distinct latent phenotype of schizotypy [39]. These functionally enriched gene-gene interactions were primarily involved in neurochemical transmission (specifically, neuropeptide and G-coupled receptors, and transmembrane signalling).

This latent phenotyping approach [8], highlighted as being valuable in the context of impulsivity-compulsivity [9], has received little application in this field to date. The overwhelming majority of studies examining neural underpinnings of impulsivity-compulsivity have used a case-control design. Current disease models of OCD and ADHD have separately implicated dysregulation of cortical regions, including the frontal lobes, responsible for the suppression of inappropriate behaviours [12, 41, 71–73]. These case-control approaches are extremely valuable. However, the manifestation of psychiatric disorders can be seen as stemming from extremely complex interactions between genetic and environmental factors, and so may be relatively “distal” to the underlying biological mechanisms explaining vulnerability [8]. Intermediate biologically-grounded phenotypes in the broader population may be more tractably linked to particular brain structural changes and expression of relevant genes. As rigorously demonstrated here using such a latent phenotyping approach, a broad range of impulsive and compulsive problems was associated with common micro-structural cortical abnormalities, namely elevated intra-cortical Magnetisation Transfer (MT) (Fig. 2). MT reflects the ratio of lipid to watery tissue in a particular brain region [74], and constitutes a developmental marker of myelination [75], being strongly correlated with actual myelination according to histology [34, 35]. There were concomitant reductions of cortical thickness. Collectively, these results are in keeping with our hypothesis that changes in cortical structure underpin disinhibition, by interfering with the ability of the cortex to sufficiently regulate urges and habits.

Due to the relatively recent emergence of imaging pipelines suitable for quantifying intra-cortical MT, there is a paucity of studies against which to compare the current results, highlighting the novelty of the study. Of note, the frontal regions associated with disinhibition we observed herein overlap with frontal regions previously found to have elevated MT in OCD patients versus controls [36]. Also of note, we found that disinhibition was associated with concomitant reductions of cortical thickness in the implicated neural regions. Previous studies have extensively documented reduced cortical thickness in disorders such as OCD [16] and ADHD [33]. The frontal lobes play a classic role in the suppression of both impulsive and compulsive response tendencies, according to neurobiological models of such disorders as ADHD [13] and OCD [12, 73, 76]. However, in addition to frontal regions, and extending beyond our initial hypothesis, higher MT in other brain regions (mainly parietal) was also significantly related to disinhibition. Tiers of evidence using other imaging techniques implicate many of these regions in impulsive and compulsive disorders, even though the traditional focus has been on the role of the frontal lobes [16, 60, 77–80].

By using data from the Allen Brain Human Atlas, we were able to infer genes, and enriched gene-gene interactions, significantly co-localised with the disinhibition-related brain map (Fig. 3 and Table 2). The set of genes was significantly enriched, in terms of gene-gene interactions, for biological processes involved in receptor signalling (peptide and G-Protein related).

Though this is the first study to explore brain substrates of disinhibition viewed across a comprehensive range of impulsive and compulsive problems, several limitations should be considered. Firstly, the current research was undertaken in a cohort recruited to be epidemiologically representative of the background population. In keeping with this, the mean scores on conventional impulsivity and compulsivity self-report scales were similar to those found in previous normative cohorts [51, 81, 82]. Though such a cohort is ideal for work on dimensional psychopathology, more extreme expression of disinhibition is to be expected in patient populations.

We did not examine neural and genetic associations with the residual impulsivity and residual compulsivity factors, since the vast majority of variance in impulsive-compulsive behaviours was explained by the disinhibition factor. By taking this approach, we do not mean to suggest that there are not *distinct* mechanisms also differentially contributing to impulsive and compulsive disorders [49]. But rather, we highlight the importance of considering common neural mechanisms related to disinhibition in future work, since this appears to be a major contributor to many forms of impulsive and compulsive behavioural manifestations, and is seldom considered in impulsivity/compulsivity research. It remains to be determined whether these findings would generalise to different cohorts, such as older participants (who may have relatively lower impulsivity). The identification of genes co-localised with disinhibition-related brain regions was by necessity an inferential analysis of gene expression in adults using the Allan Brain Atlas, since it is not possible to measure protein expression in vivo. Age and gender did not significantly explain the occurrence of disinhibition in statistical modelling. However, because Allan Brain Atlas donors were not matched to the current dataset in terms of demographic characteristics, some caution is needed when interpreting gene findings. The expression of particular forms of impulsive of compulsive problems may of course relate to these variables (e.g. antisocial tendencies are generally higher in men), indeed as previously demonstrated for residual factors using this bi-factor model [10]. Our results indicate that the common factor contributing to the full range of impulsive and compulsive problems was not significantly related to age or gender. Neuroimaging in the NSPN cohort was not conducted in all subjects, but rather on a representative subsample, as is common in cohort studies due to the relatively high cost of conducting brain scans. However, the sample size was ample to determine brain relationships, including with rigorous cross-validation procedures. Moreover, while larger imaging cohorts exist, they do not generally measure impulsive and compulsive problems sufficiently in order to quantify related dimensional phenotypes. For example, they might typically measure ADHD and OCD in binary form (presence or absence), but this is insufficient information from which to construct a valid disinhibition model. Lastly, the latent disinhibition phenotype correlated with impulsive and compulsive symptoms viewed dimensionally, including archetypal impulsive (ADHD) and compulsive (OCD) symptoms measured using standard rating scales not used to calculate the original disinhibition scores. Starting with a normative sample constitutes a vital precursor to work in patient groups, in keeping with the widely advocated Research Domain Criteria approach [8], with a view towards confirmation of truly trans-diagnostic phenotypes.

In summary, this study identified common architectural brain changes underlying a latent phenotype of impulsive and compulsive problems. The findings are directly relevant to understanding common biological processes conferring vulnerability to a range of problematic behaviours, as well as conditions such as ADHD and OCD. Future work could apply this phenotyping strategy in patient populations and evaluate the effects of existing and new treatments on this marker. We hypothesise that this latent dimensional phenotype will present in more extreme forms in such clinical groups. The latent phenotype focus is potentially valuable in order to improve disease models, but also as a means of developing treatments (including early interventions) capable of subverting those common aetiological pathways contributing to the emergence of a range of impulsive and compulsive problems.

## Funding and disclosure

This research was funded by a Clinical Fellowship from the Wellcome Trust to SRC (reference 110049/Z/15/Z & 110049/Z/15/A). The study was supported by the Neuroscience in Psychiatry Network, a strategic award from the Wellcome Trust to the University of Cambridge and University College London (095844/Z/11/Z); and by the NIHR Cambridge Biomedical Research Centre (Mental Health). R-RG was funded by Guarantors of Brain fellowship. ETB is an NIHR Senior Investigator. RAIB was funded by a British Academy Post-doctoral Fellowship. SRC consults for Promentis and Ieso Digital Health. SRC receives stipends from Elsevier from editorial work at Comprehensive Psychiatry; and at Neuroscience & Biobehavioral Reviews. JEG has received research grants from NIDA, National Center for Responsible Gaming, American Foundation for Suicide Prevention, and Forest and Roche Pharmaceuticals. JEG receives yearly compensation from Springer Publishing for acting as Editor-in-Chief of the Journal of Gambling Studies and has received royalties from Oxford University Press, American Psychiatric Publishing, Inc., Norton Press, Johns Hopkins University Press, and McGraw Hill. JT was supported by National Health and Medical Research Council (NHMRC) project grants 1050504 and 1146292. IMG consults for Lundbeck; is supported by a Wellcome Trust Strategic Award; and is Chairperson of and scientific advisor to the Peter Cundill Centre for Youth Depression Research, Centre for Addictions and Mental Health, University of Toronto. ETB is a member of the Sosei Heptares scientific advisory board and is a National Institute of Health Research Senior Investigator. MY was supported by a National Health and Medical Research Council of Australia Fellowship (#APP1117188) and the David Winston Turner Endowment Fund. The other authors report no conflicts of interest or disclosures

## Supplementary information

Supplement
