# Supplementary material for: Brain micro-architecture and disinhibition: a latent phenotyping study across 33 impulsive and compulsive behaviours
Source: Neuropsychopharmacology. 2020 Sep 12;46(2):423–31. doi: 10.1038/s41386-020-00848-9 (PMC7116462; doi:10.1038/s41386-020-00848-9)
Supplement: Supplementary file 1 — Supplement [file 41386_2020_848_MOESM1_ESM.docx]

Supplementary File - Disinhibition phenotype and MT

**Figure S1. Plots showing relationships between brain scores from the PLS model (PLS X Scores) and archetypal impulsive (ADHD, top graph) and compulsive (OCD, bottom graph) symptoms. Lines of best fit are indicated with 95% confidence intervals. The correlations were significant (rho=0.294, p=0.001; and rho=0.285, p=0.001 respectively).**

**
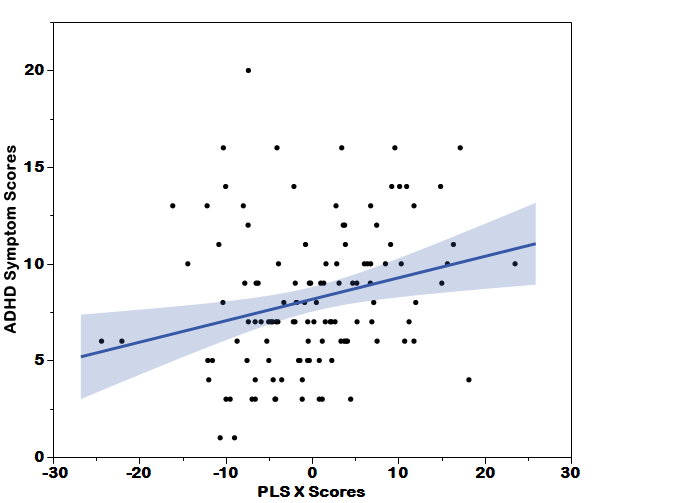

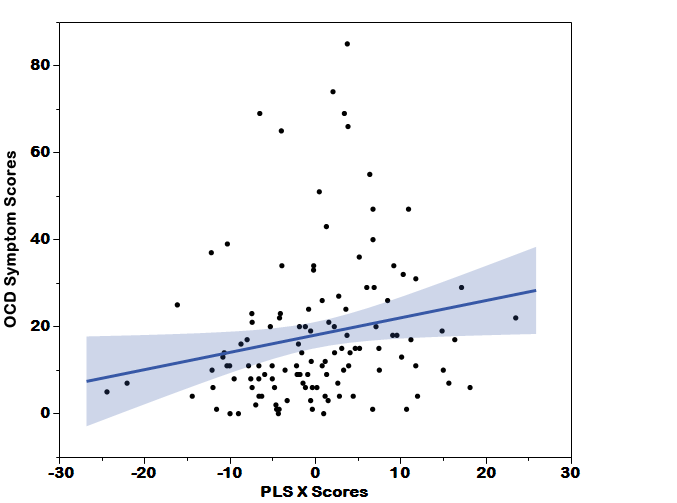
**

**Figure S2. Violin plots and histogram plots, showing distributions of disinhibition scores, and total scores on the ADHD Inventory, and Padua Inventory.**

**
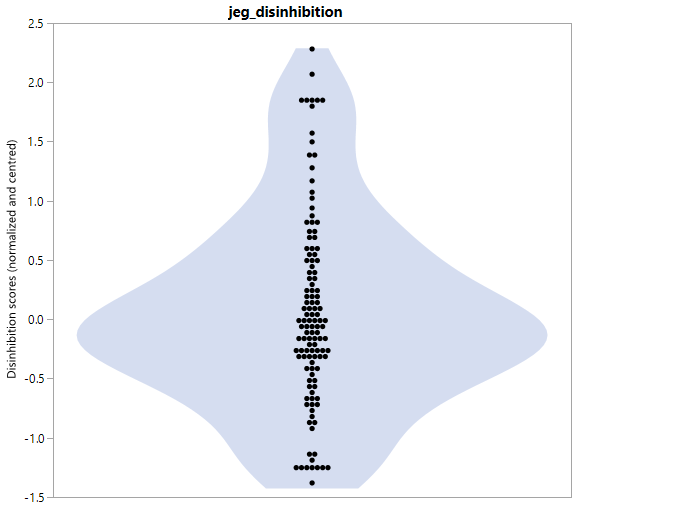
**

**
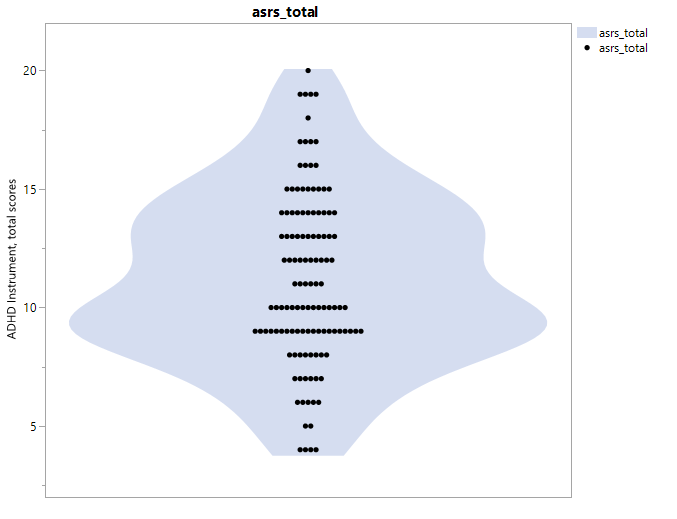
**

**
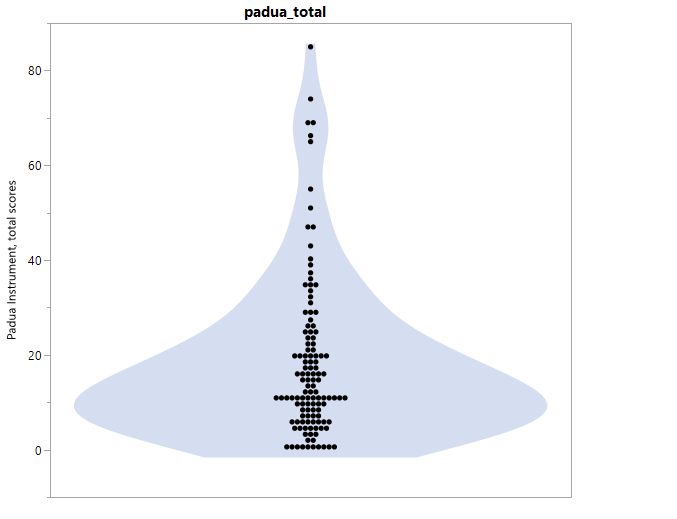
**

**Figure S3. Comparing PLS gene weights derived using different response variables.** PLS gene weights derived from disinhibition-related myelination in the manuscript (X variable) are compared with PLS gene weights of two different models (Y variable): (*i*) using both disinhibition-related myelination and disinhibition-related cortical thickness (left plot) as response variables in the same model and (*ii*) using schizotypy-related myelination as response variable (right plot, as described in Romero-Garcia *et al.* 2020, *Biological Psychiatry*).

**
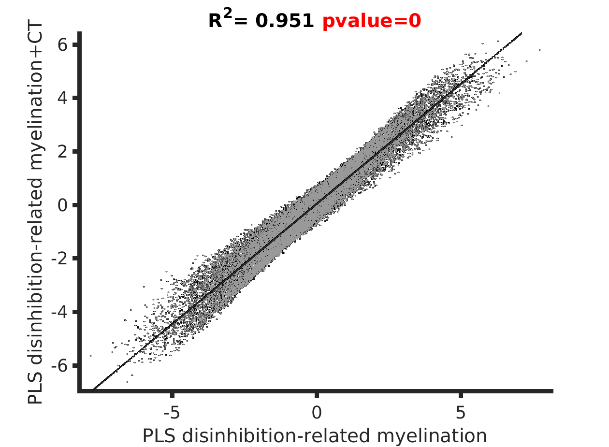

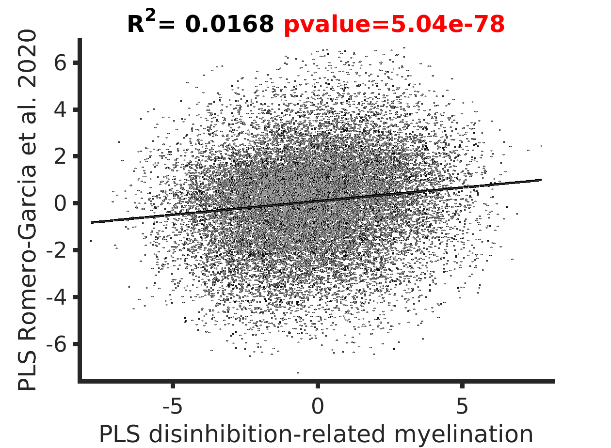
**

**Table S1**. Comparison of the imaging subgroup from NSPN against the rest of the baseline NSPN sample on age, gender, and ethnicity.

|  | Baseline NSPN  N=2331 | Imaging Subgroup  N=122 | Group Comparisons |
| --- | --- | --- | --- |
| Age (at baseline recruitment, Mean (SD)), years | 19.019 (3.023) | 18.512 (2.664) | t=1.814 ,p=0.070 |
| Gender  (Proportion female, N (%)) | 1501 (55.4%) | 76 (62.3%) | Chi-square=2.231, p=0.135 |
| Ethnicity (Proportion Caucasian, N (%)) | 1812 (94.8%) | 100 (82.6%) | Chi-square=1.336, p=0.513 |

**Table S2**. List of ICBC items corresponding to manuscript Figure 1. For each type of impulsive/compulsive behaviour, the participant is asked if THEY and OTHERS think they have a problem with the behaviour over the past 12 months, indicating: never, sometimes, often, or always (scored 1-4 accordingly). For full details of the instrument see Guo et al.., Addictive Behaviors, 2017.

ICBC Items

1. Washing

2. Smoking

3. Collecting

4. Money

5. Ordering

6. Shopping

7. List making

8. Counting

9. Grooming

10. Routines

11. Repeating

12. Exercising

13. Betting

14. Hair Picking

15. Lying

16. Sexual

17. Alcohol

18. Planning

19. Drug

20. Cleaning

21. Verbal

22. Violence

23. Swearing

24. Checking Locks

25. Checking Mirror

26. Driving

27. Medication over-use

28. Aggression

29. Social

30. Rules

31. Injury

32. Rewriting

33. Tattooing

**Table S3**. Standardized and centered model coefficients for the important X variables in the PLS model explaining variation in disinhibition scores (i.e. X variables with Variable Importance Parameter >0.8 from the initial model). Those X variables whose bootstrap distributions did not cross zero are shown with an asterisk.

| **Coefficient** | **disinhibition** |  |
| --- | --- | --- |
| **lh_bankssts_part1 *** | **0.0027** | **** |
| **lh_bankssts_part2** | **0.0023** | **** |
| **lh_caudalmiddlefrontal_part1 *** | **0.0056** | **** |
| **lh_caudalmiddlefrontal_part2 *** | **0.0043** | **** |
| **lh_caudalmiddlefrontal_part3 *** | **0.0039** | **** |
| **lh_caudalmiddlefrontal_part4 *** | **0.0043** | **** |
| **lh_cuneus_part2** | **-0.0025** | **** |
| **lh_fusiform_part1 *** | **0.0031** | **** |
| **lh_fusiform_part3** | **0.0024** | **** |
| **lh_fusiform_part5** | **0.0025** | **** |
| **lh_inferiorparietal_part1 *** | **0.0045** | **** |
| **lh_inferiorparietal_part6 *** | **0.0033** | **** |
| **lh_inferiorparietal_part8** | **0.0022** | **** |
| **lh_inferiortemporal_part2 *** | **0.0031** | **** |
| **lh_inferiortemporal_part3** | **0.0025** | **** |
| **lh_isthmuscingulate_part1** | **0.0020** | **** |
| **lh_isthmuscingulate_part2 *** | **0.0028** | **** |
| **lh_lateraloccipital_part2 *** | **0.0023** | **** |
| **lh_lingual_part1** | **0.0022** | **** |
| **lh_lingual_part3** | **0.0027** | **** |
| **lh_middletemporal_part1** | **0.0018** | **** |
| **lh_middletemporal_part2** | **0.0018** | **** |
| **lh_middletemporal_part3 *** | **0.0038** | **** |
| **lh_middletemporal_part4 *** | **0.0024** | **** |
| **lh_parahippocampal_part1** | **0.0028** | **** |
| **lh_parahippocampal_part2** | **0.0026** | **** |
| **lh_paracentral_part1** | **0.0025** | **** |
| **lh_paracentral_part2 *** | **0.0038** | **** |
| **lh_paracentral_part3 *** | **0.0037** | **** |
| **lh_parsopercularis_part1 *** | **0.0034** | **** |
| **lh_parsopercularis_part3 *** | **0.0033** | **** |
| **lh_parstriangularis_part1** | **0.0020** | **** |
| **lh_postcentral_part1 *** | **0.0034** | **** |
| **lh_postcentral_part3** | **0.0036** | **** |
| **lh_postcentral_part5 *** | **0.0046** | **** |
| **lh_postcentral_part6 *** | **0.0026** | **** |
| **lh_postcentral_part7 *** | **0.0037** | **** |
| **lh_postcentral_part8 *** | **0.0034** | **** |
| **lh_posteriorcingulate_part1 *** | **0.0031** | **** |
| **lh_posteriorcingulate_part2 *** | **0.0032** | **** |
| **lh_precentral_part1 *** | **0.0046** | **** |
| **lh_precentral_part3 *** | **0.0035** | **** |
| **lh_precentral_part5 *** | **0.0040** | **** |
| **lh_precentral_part6 *** | **0.0039** | **** |
| **lh_precentral_part8 *** | **0.0041** | **** |
| **lh_precentral_part9 *** | **0.0037** | **** |
| **lh_precuneus_part2 *** | **0.0034** | **** |
| **lh_precuneus_part4 *** | **0.0036** | **** |
| **lh_precuneus_part5** | **0.0023** | **** |
| **lh_precuneus_part6 *** | **0.0030** | **** |
| **lh_precuneus_part7** | **0.0023** | **** |
| **lh_rostralanteriorcingulate_part1** | **0.0023** | **** |
| **lh_rostralmiddlefrontal_part1** | **0.0026** | **** |
| **lh_rostralmiddlefrontal_part2** | **0.0022** | **** |
| **lh_rostralmiddlefrontal_part3 *** | **0.0033** | **** |
| **lh_rostralmiddlefrontal_part5 *** | **0.0044** | **** |
| **lh_rostralmiddlefrontal_part6** | **0.0023** | **** |
| **lh_rostralmiddlefrontal_part7 *** | **0.0033** | **** |
| **lh_rostralmiddlefrontal_part9** | **0.0031** | **** |
| **lh_superiorfrontal_part1** | **0.0022** | **** |
| **lh_superiorfrontal_part2 *** | **0.0032** | **** |
| **lh_superiorfrontal_part4 *** | **0.0042** | **** |
| **lh_superiorfrontal_part5** | **0.0025** | **** |
| **lh_superiorfrontal_part6 *** | **0.0044** | **** |
| **lh_superiorfrontal_part7** | **0.0028** | **** |
| **lh_superiorfrontal_part8 *** | **0.0042** | **** |
| **lh_superiorfrontal_part9** | **0.0027** | **** |
| **lh_superiorfrontal_part10 *** | **0.0041** | **** |
| **lh_superiorfrontal_part11 *** | **0.0045** | **** |
| **lh_superiorfrontal_part12 *** | **0.0040** | **** |
| **lh_superiorfrontal_part13 *** | **0.0032** | **** |
| **lh_superiorparietal_part1 *** | **0.0043** | **** |
| **lh_superiorparietal_part2** | **-0.0022** | **** |
| **lh_superiorparietal_part3 *** | **0.0055** | **** |
| **lh_superiorparietal_part4 *** | **0.0044** | **** |
| **lh_superiorparietal_part5 *** | **0.0033** | **** |
| **lh_superiorparietal_part7 *** | **0.0046** | **** |
| **lh_superiorparietal_part9 *** | **0.0033** | **** |
| **lh_superiorparietal_part10 *** | **0.0035** | **** |
| **lh_superiortemporal_part3** | **0.0021** | **** |
| **lh_superiortemporal_part5** | **0.0021** | **** |
| **lh_supramarginal_part1 *** | **0.0038** | **** |
| **lh_supramarginal_part3 *** | **0.0038** | **** |
| **lh_supramarginal_part4** | **0.0019** | **** |
| **lh_supramarginal_part5 *** | **0.0031** | **** |
| **lh_supramarginal_part6 *** | **0.0040** | **** |
| **lh_supramarginal_part7** | **0.0028** | **** |
| **lh_insula_part1** | **0.0024** | **** |
| **rh_bankssts_part1** | **0.0019** | **** |
| **rh_caudalmiddlefrontal_part1** | **0.0028** | **** |
| **rh_caudalmiddlefrontal_part2** | **0.0025** | **** |
| **rh_caudalmiddlefrontal_part3 *** | **0.0040** | **** |
| **rh_caudalmiddlefrontal_part4 *** | **0.0032** | **** |
| **rh_paracentral_part1 *** | **0.0027** | **** |
| **rh_paracentral_part2 *** | **0.0032** | **** |
| **rh_paracentral_part3** | **0.0022** | **** |
| **rh_parstriangularis_part1** | **0.0029** | **** |
| **rh_parstriangularis_part2** | **0.0023** |  |
| **rh_parstriangularis_part3 *** | **0.0033** |  |
| **rh_postcentral_part1 *** | **0.0035** |  |
| **rh_postcentral_part3** | **0.0024** |  |
| **rh_postcentral_part5** | **0.0027** |  |
| **rh_postcentral_part6** | **0.0021** |  |
| **rh_postcentral_part7 *** | **0.0035** |  |
| **rh_postcentral_part8** | **0.0027** |  |
| **rh_posteriorcingulate_part1** | **0.0020** |  |
| **rh_posteriorcingulate_part2 *** | **0.0031** |  |
| **rh_precentral_part2** | **0.0030** |  |
| **rh_precentral_part4 *** | **0.0038** |  |
| **rh_precentral_part6** | **0.0021** |  |
| **rh_precentral_part7** | **0.0019** |  |
| **rh_precentral_part8** | **0.0029** |  |
| **rh_precentral_part9 *** | **0.0035** |  |
| **rh_precuneus_part3 *** | **0.0047** |  |
| **rh_precuneus_part5** | **0.0024** |  |
| **rh_rostralanteriorcingulate_part1** | **0.0018** |  |
| **rh_rostralmiddlefrontal_part3 *** | **0.0039** |  |
| **rh_rostralmiddlefrontal_part5 *** | **0.0035** |  |
| **rh_rostralmiddlefrontal_part6** | **0.0024** |  |
| **rh_rostralmiddlefrontal_part7** | **0.0027** |  |
| **rh_rostralmiddlefrontal_part8** | **0.0033** |  |
| **rh_rostralmiddlefrontal_part10** | **0.0024** |  |
| **rh_superiorfrontal_part1 *** | **0.0036** |  |
| **rh_superiorfrontal_part3 *** | **0.0050** |  |
| **rh_superiorfrontal_part5 *** | **0.0042** |  |
| **rh_superiorfrontal_part6** | **0.0027** |  |
| **rh_superiorfrontal_part7 *** | **0.0039** |  |
| **rh_superiorfrontal_part8** | **0.0027** |  |
| **rh_superiorfrontal_part9 *** | **0.0043** |  |
| **rh_superiorfrontal_part10** | **0.0024** |  |
| **rh_superiorfrontal_part11 *** | **0.0036** |  |
| **rh_superiorfrontal_part12 *** | **0.0040** |  |
| **rh_superiorfrontal_part13 *** | **0.0037** |  |
| **rh_superiorparietal_part1** | **-0.0022** |  |
| **rh_superiorparietal_part2 *** | **0.0037** |  |
| **rh_superiorparietal_part6 *** | **0.0039** |  |
| **rh_superiorparietal_part7** | **0.0022** |  |
| **rh_superiorparietal_part8** | **0.0037** |  |
| **rh_superiorparietal_part9 *** | **0.0036** |  |
| **rh_supramarginal_part3** | **0.0027** |  |
| **rh_supramarginal_part6 *** | **0.0035** |  |

**Table S4**. List of genes significantly enriched in the brain parcels linking disinhibition to MT, at FDR p<0.05 corrected.

| Gene | GeneID | Z-score | FDR corrected p |
| --- | --- | --- | --- |
| C20orf195 | 2592 | 7.751098 | <0.000001 |
| CKAP4 | 4013 | 7.294347 | <0.000001 |
| C6orf105 | 2858 | 6.908775 | <0.000001 |
| KLK7 | 9288 | 6.74292 | <0.000001 |
| SH2D1B | 16428 | 6.668291 | <0.000001 |
| WTIP | 19751 | 6.55314 | <0.000001 |
| RP11-427P5.1 | 15596 | 6.497663 | <0.000001 |
| ADRA1B | 645 | 6.459265 | <0.000001 |
| ALAS2 | 815 | 6.458091 | <0.000001 |
| CDH22 | 3687 | 6.439371 | <0.000001 |
| ZC3H12D | 19925 | 6.351452 | <0.000001 |
| SPRR2G | 17328 | 6.343957 | <0.000001 |
| OR2M3 | 12939 | 6.328685 | <0.000001 |
| C10orf57 | 2034 | 6.298717 | <0.000001 |
| SEMG2 | 16240 | 6.295928 | <0.000001 |
| LOC339524 | 10325 | 6.235703 | <0.000001 |
| LOC284440 | 10301 | 6.233439 | <0.000001 |
| TMEM196 | 18376 | 6.200323 | <0.000001 |
| HRH1 | 8195 | 6.150933 | <0.000001 |
| SERPINB2 | 16305 | 6.136128 | <0.000001 |
| PCDH17 | 13453 | 6.126003 | <0.000001 |
| C22orf43 | 2668 | 6.124394 | <0.000001 |
| FREM3 | 6787 | 6.072065 | 0.000001 |
| KRT33B | 9352 | 6.070032 | 0.000001 |
| AC093283.3 | 318 | 6.055006 | 0.000001 |
| PKP3 | 13937 | 6.052422 | 0.000001 |
| KRT31 | 9349 | 6.040382 | 0.000001 |
| THEG | 18106 | 6.031542 | 0.000001 |
| LDHD | 9597 | 6.021323 | 0.000001 |
| OVOL2 | 13255 | 6.018364 | 0.000001 |
| RTP1 | 15917 | 6.015258 | 0.000001 |
| PCDHAC2 | 13470 | 6.014207 | 0.000001 |
| TUBA4B | 19053 | 6.008714 | 0.000001 |
| TWIST2 | 19090 | 5.988412 | 0.000001 |
| TSKU | 18911 | 5.974435 | 0.000001 |
| WNT7A | 19733 | 5.966687 | 0.000001 |
| NTNG2 | 12626 | 5.93917 | 0.000001 |
| LOC375295 | 10338 | 5.93786 | 0.000001 |
| SH3PXD2B | 16455 | 5.915983 | 0.000001 |
| COL11A1 | 4238 | 5.90597 | 0.000001 |
| CRYGN | 4496 | 5.890403 | 0.000001 |
| ACTA1 | 496 | 5.879545 | 0.000001 |
| AMIGO2 | 907 | 5.865529 | 0.000001 |
| TM7SF4 | 18229 | 5.846269 | 0.000001 |
| LOC400027 | 10377 | 5.837448 | 0.000001 |
| GUK1 | 7682 | 5.820768 | 0.000001 |
| CCDC3 | 3372 | 5.82072 | 0.000001 |
| GCK | 7051 | 5.81383 | 0.000001 |
| PNPLA3 | 14122 | 5.796576 | 0.000001 |
| RPL36 | 15758 | 5.794281 | 0.000001 |
| AC013356.1 | 211 | 5.783285 | 0.000001 |
| RABEP1 | 14939 | 5.7729 | 0.000002 |
| GDNF | 7082 | 5.768839 | 0.000002 |
| LRRC4C | 10830 | 5.766789 | 0.000002 |
| GNA15 | 7275 | 5.755643 | 0.000002 |
| LOC100130100 | 9927 | 5.747973 | 0.000002 |
| CNTN6 | 4215 | 5.741279 | 0.000002 |
| ADAMTS3 | 576 | 5.717373 | 0.000002 |
| SF3A2 | 16353 | 5.714046 | 0.000002 |
| CDC2 | 3634 | 5.704669 | 0.000002 |
| OR14I1 | 12872 | 5.700364 | 0.000002 |
| LOC100129122 | 9896 | 5.69981 | 0.000002 |
| CBLN2 | 3273 | 5.695015 | 0.000002 |
| ABCG5 | 81 | 5.681767 | 0.000002 |
| GPR120 | 7411 | 5.680556 | 0.000002 |
| PPEF1 | 14269 | 5.675966 | 0.000002 |
| GAST | 7009 | 5.65011 | 0.000002 |
| RP11-535K1.1 | 15617 | 5.630149 | 0.000003 |
| TUBA4A | 19052 | 5.627799 | 0.000003 |
| RDH11 | 15173 | 5.618235 | 0.000003 |
| GYPE | 7696 | 5.607408 | 0.000003 |
| GPR68 | 7486 | 5.600485 | 0.000003 |
| LOC100289263 | 10162 | 5.59962 | 0.000003 |
| ASGR2 | 1425 | 5.597611 | 0.000003 |
| AC130454.1 | 373 | 5.595133 | 0.000003 |
| YIF1B | 19827 | 5.592909 | 0.000003 |
| LY6G6C | 10921 | 5.575164 | 0.000003 |
| LOC100127925 | 9862 | 5.569753 | 0.000003 |
| MYBPC2 | 11910 | 5.545306 | 0.000004 |
| SPRY4 | 17334 | 5.544158 | 0.000004 |
| GXYLT2 | 7690 | 5.542236 | 0.000004 |
| GSG1 | 7601 | 5.540995 | 0.000004 |
| MUM1L1 | 11881 | 5.525569 | 0.000004 |
| FJX1 | 6599 | 5.519412 | 0.000004 |
| UBQLNL | 19209 | 5.513712 | 0.000004 |
| CRLF1 | 4459 | 5.506681 | 0.000004 |
| SPON2 | 17310 | 5.501924 | 0.000004 |
| RP11-520B13.4 | 15615 | 5.50171 | 0.000004 |
| C1orf183 | 2497 | 5.501129 | 0.000004 |
| DNLZ | 5293 | 5.493806 | 0.000005 |
| ARHGAP25 | 1260 | 5.491703 | 0.000005 |
| FAM136BP | 6116 | 5.481413 | 0.000005 |
| PRRX1 | 14580 | 5.477626 | 0.000005 |
| PSORS1C1 | 14681 | 5.47528 | 0.000005 |
| GRIN3A | 7563 | 5.464952 | 0.000005 |
| MUCL1 | 11877 | 5.461429 | 0.000005 |
| TRIB1 | 18756 | 5.45653 | 0.000005 |
| STMN2 | 17552 | 5.450743 | 0.000005 |
| CBLN1 | 3272 | 5.444931 | 0.000005 |
| MDGA1 | 11249 | 5.44478 | 0.000005 |
| LCP2 | 9586 | 5.442267 | 0.000005 |
| LCN1 | 9574 | 5.440661 | 0.000005 |
| AKR1C4 | 781 | 5.436715 | 0.000005 |
| AKR1C1 | 778 | 5.423933 | 0.000006 |
| CALHM1 | 3146 | 5.422404 | 0.000006 |
| WFDC1 | 19685 | 5.420101 | 0.000006 |
| DHRS7C | 5106 | 5.416238 | 0.000006 |
| LGALS2 | 9639 | 5.415023 | 0.000006 |
| DUXAP10 | 5475 | 5.41392 | 0.000006 |
| GPR20 | 7458 | 5.412703 | 0.000006 |
| PLCH1 | 13987 | 5.406006 | 0.000006 |
| LOC100294469 | 10237 | 5.393669 | 0.000006 |
| CROCCP3 | 4469 | 5.386892 | 0.000007 |
| RP11-178A10.1 | 15546 | 5.378964 | 0.000007 |
| ABHD1 | 83 | 5.375587 | 0.000007 |
| TNNT2 | 18591 | 5.369212 | 0.000007 |
| DRD5 | 5388 | 5.362695 | 0.000007 |
| CGREF1 | 3869 | 5.358715 | 0.000007 |
| AMAC1L2 | 892 | 5.352891 | 0.000008 |
| RNF150 | 15434 | 5.348479 | 0.000008 |
| LINC00545 | 9796 | 5.345976 | 0.000008 |
| WFDC12 | 19689 | 5.344024 | 0.000008 |
| TMEM159 | 18329 | 5.343571 | 0.000008 |
| PCDH21 | 13457 | 5.342388 | 0.000008 |
| AC018865.8 | 228 | 5.336649 | 0.000008 |
| HTR1E | 8289 | 5.334331 | 0.000008 |
| CLSTN1 | 4132 | 5.32782 | 0.000008 |
| CXCL14 | 4684 | 5.325261 | 0.000008 |
| SH2B2 | 16425 | 5.323042 | 0.000008 |
| GPR17 | 7443 | 5.30975 | 0.000009 |
| TSPAN33 | 18933 | 5.305042 | 0.000009 |
| RP1-21O18.1 | 15526 | 5.302117 | 0.000009 |
| ZNF648 | 20434 | 5.297552 | 0.000009 |
| SORCS2 | 17154 | 5.296453 | 0.000009 |
| LOC727938 | 10597 | 5.294392 | 0.000009 |
| KIF17 | 9154 | 5.289061 | 0.000009 |
| CENPF | 3793 | 5.281486 | 0.00001 |
| PRAP1 | 14409 | 5.280932 | 0.00001 |
| CROT | 4470 | 5.274783 | 0.00001 |
| LOC138864 | 10250 | 5.273957 | 0.00001 |
| C4orf19 | 2785 | 5.273374 | 0.00001 |
| CLPS | 4124 | 5.272802 | 0.00001 |
| S100A6 | 15968 | 5.270707 | 0.00001 |
| COL5A3 | 4270 | 5.267722 | 0.00001 |
| GYPB | 7694 | 5.264522 | 0.00001 |
| DAND5 | 4846 | 5.256454 | 0.00001 |
| IER5 | 8360 | 5.254367 | 0.00001 |
| WIPF2 | 19708 | 5.240274 | 0.000011 |
| C22orf26 | 2655 | 5.238654 | 0.000011 |
| KCNJ8 | 8918 | 5.226351 | 0.000012 |
| CHRNB1 | 3954 | 5.221157 | 0.000012 |
| PRSS1 | 14582 | 5.218452 | 0.000012 |
| PRODH2 | 14520 | 5.216208 | 0.000012 |
| GCNT1 | 7058 | 5.215289 | 0.000012 |
| OR6V1 | 13134 | 5.208005 | 0.000013 |
| MEI1 | 11310 | 5.203683 | 0.000013 |
| FAM19A5 | 6204 | 5.203142 | 0.000013 |
| GAL | 6950 | 5.200777 | 0.000013 |
| GPR143 | 7426 | 5.194436 | 0.000013 |
| NOTUM | 12467 | 5.18865 | 0.000014 |
| ANKK1 | 959 | 5.179973 | 0.000014 |
| C21orf110 | 2623 | 5.176352 | 0.000014 |
| GAS2L3 | 7004 | 5.169495 | 0.000015 |
| S100A5 | 15967 | 5.168138 | 0.000015 |
| RBP1 | 15137 | 5.161931 | 0.000015 |
| GPR153 | 7435 | 5.148646 | 0.000016 |
| FGFR1 | 6560 | 5.147998 | 0.000016 |
| CTXN3 | 4651 | 5.143412 | 0.000017 |
| FAM163A | 6149 | 5.139362 | 0.000017 |
| SLC39A11 | 16817 | 5.138105 | 0.000017 |
| WHSC1 | 19702 | 5.128514 | 0.000018 |
| CEACAM4 | 3761 | 5.127646 | 0.000018 |
| C1QL4 | 2434 | 5.12729 | 0.000018 |
| COL16A1 | 4244 | 5.120918 | 0.000018 |
| TUBB7P | 19063 | 5.119285 | 0.000018 |
| RPAP1 | 15700 | 5.116786 | 0.000018 |
| OLFM3 | 12779 | 5.10929 | 0.000019 |
| ISLR2 | 8722 | 5.103844 | 0.000019 |
| GGT8P | 7128 | 5.099756 | 0.00002 |
| NAP1L6 | 12057 | 5.099181 | 0.00002 |
| HMX2 | 8081 | 5.098716 | 0.00002 |
| C10orf114 | 2004 | 5.096092 | 0.00002 |
| KCNMB1 | 8936 | 5.095638 | 0.00002 |
| CD63 | 3602 | 5.095146 | 0.00002 |
| CTC1 | 4593 | 5.088799 | 0.00002 |
| PCDH24 | 13458 | 5.087942 | 0.00002 |
| AC183087.2 | 405 | 5.085072 | 0.00002 |
| LOC339240 | 10321 | 5.077086 | 0.000021 |
| PAX6 | 13422 | 5.072343 | 0.000021 |
| SBF1P1 | 16047 | 5.066578 | 0.000022 |
| CDH10 | 3676 | 5.066292 | 0.000022 |
| CSF3R | 4517 | 5.064673 | 0.000022 |
| SNAI2 | 17036 | 5.060551 | 0.000022 |
| AC007688.2 | 154 | 5.055174 | 0.000023 |
| SLC35G4 | 16795 | 5.0547 | 0.000023 |
| P2RY2 | 13284 | 5.053513 | 0.000023 |
| OPRK1 | 12810 | 5.051821 | 0.000023 |
| SLC35F1 | 16790 | 5.048084 | 0.000023 |
| C9orf24 | 3034 | 5.045594 | 0.000023 |
| CYP26A1 | 4765 | 5.03506 | 0.000025 |
| LOC440871 | 10427 | 5.034779 | 0.000025 |
| FZD9 | 6889 | 5.032119 | 0.000025 |
| PDZD7 | 13646 | 5.031031 | 0.000025 |
| ZNF433 | 20272 | 5.03047 | 0.000025 |
| PRB1 | 14410 | 5.028902 | 0.000025 |
| HES4 | 7872 | 5.025685 | 0.000025 |
| MORF4 | 11586 | 5.002575 | 0.000028 |
| HAUS7 | 7761 | 5.000416 | 0.000028 |
| LGALS1 | 9634 | 4.995201 | 0.000029 |
| LYZL4 | 10962 | 4.984492 | 0.000031 |
| SALL3 | 15999 | 4.982088 | 0.000031 |
| SARDH | 16029 | 4.981946 | 0.000031 |
| IL2RB | 8541 | 4.979391 | 0.000031 |
| RAB39 | 14904 | 4.979276 | 0.000031 |
| AC078937.4 | 289 | 4.973166 | 0.000032 |
| TTYH1 | 19042 | 4.964731 | 0.000033 |
| C13orf33 | 2167 | 4.962069 | 0.000033 |
| TEX26 | 18028 | 4.958952 | 0.000034 |
| PF4V1 | 13695 | 4.9576 | 0.000034 |
| SLN | 16959 | 4.953935 | 0.000034 |
| RGL3 | 15266 | 4.946693 | 0.000035 |
| TFPT | 18059 | 4.946398 | 0.000035 |
| NPCDR1 | 12488 | 4.943946 | 0.000035 |
| NPFFR2 | 12494 | 4.94317 | 0.000035 |
| COL5A2 | 4269 | 4.935863 | 0.000037 |
| SLC22A18 | 16646 | 4.935179 | 0.000037 |
| DDAH1 | 4930 | 4.92897 | 0.000037 |
| TUBB8 | 19064 | 4.92819 | 0.000037 |
| DPEP1 | 5338 | 4.927879 | 0.000037 |
| NR1H3 | 12535 | 4.927399 | 0.000037 |
| SEC16B | 16186 | 4.924469 | 0.000038 |
| IRGC | 8702 | 4.923707 | 0.000038 |
| MDFIC | 11248 | 4.922802 | 0.000038 |
| PDLIM3 | 13622 | 4.920533 | 0.000038 |
| GREM2 | 7541 | 4.914044 | 0.000039 |
| NTN1 | 12621 | 4.905964 | 0.000041 |
| TM7SF2 | 18227 | 4.904382 | 0.000041 |
| EPB41L4A | 5810 | 4.902417 | 0.000041 |
| TMEM132A | 18297 | 4.902319 | 0.000041 |
| GALNTL5 | 6978 | 4.899151 | 0.000041 |
| FMO1 | 6694 | 4.881961 | 0.000045 |
| GNG5 | 7302 | 4.881822 | 0.000045 |
| VIT | 19489 | 4.875338 | 0.000046 |
| MTL5 | 11824 | 4.870893 | 0.000047 |
| DEFA5 | 4999 | 4.870731 | 0.000047 |
| LRRC42 | 10822 | 4.870273 | 0.000047 |
| TCP10L | 17954 | 4.867391 | 0.000047 |
| LIFR | 9679 | 4.866936 | 0.000047 |
| AC134312.1 | 379 | 4.864378 | 0.000048 |
| DYTN | 5502 | 4.859082 | 0.000049 |
| FITM1 | 6596 | 4.855457 | 0.000049 |
| GALR2 | 6982 | 4.853956 | 0.00005 |
| TGFBI | 18069 | 4.851664 | 0.00005 |
| TTTY4B | 19036 | 4.851298 | 0.00005 |
| DNAJB1 | 5237 | 4.847877 | 0.000051 |
| CAMK1G | 3163 | 4.846428 | 0.000051 |
| CD52 | 3595 | 4.845733 | 0.000051 |
| FRMPD2 | 6803 | 4.836723 | 0.000053 |
| TRIM36 | 18784 | 4.829061 | 0.000055 |
| TSPAN17 | 18926 | 4.826897 | 0.000055 |
| AC109486.1 | 350 | 4.823068 | 0.000056 |
| TADA3L | 17749 | 4.819954 | 0.000057 |
| DIO2 | 5135 | 4.817519 | 0.000057 |
| HSPB1 | 8266 | 4.815912 | 0.000057 |
| ARNT2 | 1349 | 4.815879 | 0.000057 |
| PKLR | 13927 | 4.811881 | 0.000058 |
| FSTL5 | 6828 | 4.801803 | 0.000061 |
| FTHL16 | 6832 | 4.801021 | 0.000061 |
| AQP9 | 1220 | 4.800443 | 0.000061 |
| NOS3 | 12458 | 4.798464 | 0.000061 |
| AC004241.1 | 123 | 4.795206 | 0.000062 |
| HYAL3 | 8315 | 4.791049 | 0.000063 |
| CHRNB3 | 3956 | 4.785574 | 0.000064 |
| LY86-AS1 | 10929 | 4.780074 | 0.000066 |
| AC010087.3 | 181 | 4.774726 | 0.000067 |
| CD81 | 3612 | 4.773953 | 0.000067 |
| MYO5A | 11977 | 4.773217 | 0.000067 |
| PRDM16 | 14425 | 4.7729 | 0.000067 |
| FTH1P20 | 6831 | 4.767933 | 0.000069 |
| PRG3 | 14449 | 4.766376 | 0.000069 |
| CCDC68 | 3409 | 4.765756 | 0.000069 |
| TRAM2 | 18724 | 4.763083 | 0.00007 |
| NIPAL1 | 12336 | 4.762742 | 0.00007 |
| HPSE2 | 8183 | 4.76093 | 0.00007 |
| CCDC110 | 3314 | 4.759401 | 0.00007 |
| SKAP2 | 16551 | 4.758187 | 0.000071 |
| SH3BGRL3 | 16440 | 4.75578 | 0.000071 |
| LOC728470 | 10616 | 4.754119 | 0.000071 |
| TP53BP2 | 18647 | 4.751575 | 0.000072 |
| CXCL3 | 4688 | 4.751407 | 0.000072 |
| EFHC2 | 5593 | 4.735489 | 0.000078 |
| CDH13 | 3679 | 4.732972 | 0.000078 |
| HDDC2 | 7821 | 4.729737 | 0.000079 |
| PVRL3 | 14814 | 4.729337 | 0.000079 |
| ZNF469 | 20295 | 4.727852 | 0.000079 |
| SP5 | 17191 | 4.724849 | 0.00008 |
| BMP3 | 1855 | 4.724124 | 0.00008 |
| BEND5 | 1795 | 4.722543 | 0.000081 |
| PHLDA2 | 13794 | 4.721024 | 0.000081 |
| NWD1 | 12705 | 4.718575 | 0.000082 |
| FBXW7 | 6466 | 4.714375 | 0.000083 |
| TSPAN10 | 18919 | 4.714062 | 0.000083 |
| SAMD4A | 16009 | 4.711425 | 0.000084 |
| TNFAIP8 | 18537 | 4.705721 | 0.000086 |
| C7orf68 | 2964 | 4.704338 | 0.000086 |
| CD6 | 3601 | 4.703987 | 0.000086 |
| FBP2 | 6400 | 4.703221 | 0.000086 |
| CLIC5 | 4099 | 4.699866 | 0.000087 |
| UNQ9391 | 19308 | 4.692265 | 0.00009 |
| CLRN3 | 4130 | 4.691855 | 0.00009 |
| RASGEF1C | 15046 | 4.685907 | 0.000092 |
| CAT | 3254 | 4.685654 | 0.000092 |
| GPR152 | 7434 | 4.676431 | 0.000096 |
| KRT5 | 9361 | 4.672385 | 0.000098 |
| OR2W3 | 12958 | 4.671874 | 0.000098 |
| AC100803.1 | 334 | 4.662714 | 0.000102 |
| FAM64A | 6286 | 4.660539 | 0.000103 |
| CHST15 | 3967 | 4.66027 | 0.000103 |
| ANP32A-IT1 | 1050 | 4.658949 | 0.000103 |
| FITM2 | 6597 | 4.65833 | 0.000103 |
| RNF222 | 15468 | 4.657443 | 0.000103 |
| RP5-1027G4.3 | 15681 | 4.656534 | 0.000103 |
| GRASP | 7535 | 4.652619 | 0.000105 |
| GNA14 | 7274 | 4.649094 | 0.000106 |
| ADRA2C | 649 | 4.645163 | 0.000108 |
| UBTD2 | 19217 | 4.637543 | 0.000112 |
| FAM43B | 6250 | 4.63514 | 0.000113 |
| KRT78 | 9373 | 4.63369 | 0.000113 |
| C15orf41 | 2245 | 4.633581 | 0.000113 |
| SPSB4 | 17341 | 4.630432 | 0.000114 |
| VSX1 | 19553 | 4.626145 | 0.000116 |
| C6orf201 | 2892 | 4.625988 | 0.000116 |
| TSFM | 18898 | 4.620893 | 0.000118 |
| SMPX | 17019 | 4.617577 | 0.00012 |
| LBH | 9543 | 4.615252 | 0.000121 |
| AC062028.1 | 263 | 4.611632 | 0.000123 |
| GNB4 | 7292 | 4.610955 | 0.000123 |
| KIRREL2 | 9200 | 4.6088 | 0.000124 |
| CSN1S1 | 4528 | 4.607491 | 0.000124 |
| APOA4 | 1170 | 4.604376 | 0.000126 |
| ETV2 | 5936 | 4.603296 | 0.000126 |
| TIMP4 | 18171 | 4.602227 | 0.000126 |
| CD7 | 3605 | 4.5978 | 0.000128 |
| CRYZ | 4500 | 4.597214 | 0.000128 |
| C22ORF45 | 2649 | 4.593812 | 0.00013 |
| GAP43 | 6989 | 4.589127 | 0.000133 |
| POU3F3 | 14234 | 4.586197 | 0.000134 |
| TTTY13 | 19027 | 4.585722 | 0.000134 |
| MAPK4 | 11117 | 4.585323 | 0.000134 |
| ANO3 | 1042 | 4.584291 | 0.000134 |
| SLURP1 | 16963 | 4.582583 | 0.000135 |
| CD1B | 3547 | 4.577122 | 0.000138 |
| ACPP | 472 | 4.57704 | 0.000138 |
| AC116165.2 | 360 | 4.574104 | 0.000139 |
| EIF4B | 5679 | 4.56763 | 0.000143 |
| OSCAR | 13213 | 4.564488 | 0.000145 |
| FKBP2 | 6606 | 4.563029 | 0.000146 |
| DOK7 | 5328 | 4.56232 | 0.000146 |
| PLA2G3 | 13950 | 4.550733 | 0.000154 |
| PRSS12 | 14583 | 4.55005 | 0.000154 |
| GRM3 | 7578 | 4.549348 | 0.000154 |
| LIME1 | 9700 | 4.545644 | 0.000156 |
| OR10K1 | 12838 | 4.545228 | 0.000156 |
| LRP1B | 10773 | 4.544878 | 0.000156 |
| AC226150.1 | 408 | 4.541234 | 0.000158 |
| SOX5 | 17178 | 4.541189 | 0.000158 |
| LOC401021 | 10390 | 4.540225 | 0.000158 |
| RS1 | 15877 | 4.539865 | 0.000158 |
| HGF | 7891 | 4.539475 | 0.000158 |
| C14orf177 | 2204 | 4.53575 | 0.00016 |
| PRAC | 14389 | 4.534656 | 0.00016 |
| LOC100291206 | 10206 | 4.534644 | 0.00016 |
| MESDC2 | 11327 | 4.529242 | 0.000164 |
| TFRC | 18061 | 4.527544 | 0.000164 |
| STBD1 | 17517 | 4.527278 | 0.000164 |
| SPRY1 | 17331 | 4.526827 | 0.000164 |
| AIRE | 748 | 4.519109 | 0.00017 |
| C1orf21 | 2510 | 4.517909 | 0.00017 |
| FUT6 | 6860 | 4.51789 | 0.00017 |
| RP11-694I15.6 | 15638 | 4.515283 | 0.000172 |
| OVOL3 | 13256 | 4.513604 | 0.000173 |
| GPR26 | 7462 | 4.511065 | 0.000174 |
| OPRM1 | 12812 | 4.506697 | 0.000177 |
| NFKBIE | 12290 | 4.504604 | 0.000179 |
| GRB14 | 7537 | 4.501865 | 0.000181 |
| LOC642031 | 10457 | 4.497523 | 0.000184 |
| PLEKHA7 | 14011 | 4.496834 | 0.000184 |
| CCKBR | 3452 | 4.495939 | 0.000184 |
| OSBPL3 | 13207 | 4.493091 | 0.000186 |
| TNFSF14 | 18567 | 4.490865 | 0.000187 |
| GALNT14 | 6966 | 4.490776 | 0.000187 |
| FOXA3 | 6732 | 4.48908 | 0.000188 |
| RP3-410C9.1 | 15663 | 4.486486 | 0.00019 |
| LYPD6 | 10943 | 4.485974 | 0.00019 |
| CACNB1 | 3116 | 4.48501 | 0.00019 |
| GUCA1B | 7669 | 4.484616 | 0.00019 |
| CA10 | 3074 | 4.483378 | 0.000191 |
| GALNTL6 | 6979 | 4.482727 | 0.000191 |
| SIX6 | 16549 | 4.48148 | 0.000192 |
| ENSAP3 | 5789 | 4.481232 | 0.000192 |
| ADCY7 | 609 | 4.477395 | 0.000195 |
| SCRT2 | 16135 | 4.474821 | 0.000196 |
| TCERG1L | 17928 | 4.472183 | 0.000198 |
| LOC100133794 | 10047 | 4.471571 | 0.000198 |
| ENO3 | 5775 | 4.470835 | 0.000199 |
| ACOX3 | 465 | 4.469456 | 0.000199 |
| TOMM20L | 18616 | 4.468228 | 0.0002 |
| CLCN5 | 4037 | 4.467136 | 0.000201 |
| LAIR2 | 9494 | 4.465898 | 0.000201 |
| SLC36A3 | 16798 | 4.464556 | 0.000202 |
| LRRC8A | 10848 | 4.464324 | 0.000202 |
| NEK2 | 12230 | 4.463584 | 0.000202 |
| KRTAP2-4 | 9423 | 4.460953 | 0.000204 |
| ST5 | 17460 | 4.459466 | 0.000205 |
| C6orf126 | 2866 | 4.456828 | 0.000207 |
| NDUFS5P2 | 12199 | 4.455408 | 0.000208 |
| FAM98A | 6350 | 4.452511 | 0.00021 |
| ANXA4 | 1070 | 4.448948 | 0.000213 |
| INSL3 | 8624 | 4.448633 | 0.000213 |
| EPHB3 | 5831 | 4.44822 | 0.000213 |
| LOC100130000 | 9923 | 4.445866 | 0.000215 |
| TCL1A | 17946 | 4.440977 | 0.000219 |
| FAM129B | 6100 | 4.439393 | 0.00022 |
| PRR10 | 14554 | 4.436482 | 0.000223 |
| LBX2-AS1 | 9548 | 4.43398 | 0.000224 |
| SCG2 | 16083 | 4.433693 | 0.000224 |
| LOC729538 | 10665 | 4.431172 | 0.000226 |
| BHMT | 1820 | 4.43108 | 0.000226 |
| OR2M5 | 12941 | 4.430355 | 0.000226 |
| GSX1 | 7632 | 4.429359 | 0.000227 |
| MGC50722 | 11416 | 4.428099 | 0.000228 |
| TMEM102 | 18270 | 4.426427 | 0.000229 |
| SLCO2A1 | 16931 | 4.425097 | 0.00023 |
| RP11-844P9.2 | 15648 | 4.423311 | 0.000231 |
| TWIST1 | 19089 | 4.419203 | 0.000235 |
| RBPMS2 | 15146 | 4.41879 | 0.000235 |
| C12orf43 | 2127 | 4.416562 | 0.000237 |
| CBS | 3279 | 4.414654 | 0.000238 |
| ADAMTS9 | 582 | 4.409871 | 0.000243 |
| CHIT1 | 3913 | 4.403737 | 0.00025 |
| RHPN1 | 15334 | 4.402218 | 0.000251 |
| TUBB2A | 19059 | 4.39987 | 0.000253 |
| PRKCD | 14477 | 4.397323 | 0.000255 |
| COL23A1 | 4253 | 4.396443 | 0.000256 |
| OR10A4 | 12817 | 4.393701 | 0.000258 |
| CD244 | 3560 | 4.393556 | 0.000258 |
| CRYBB1 | 4487 | 4.392261 | 0.000259 |
| THEM5 | 18108 | 4.389768 | 0.000261 |
| RNF213 | 15460 | 4.385752 | 0.000266 |
| LOC643438 | 10474 | 4.384731 | 0.000266 |
| PPBP | 14263 | 4.382878 | 0.000268 |
| TNN | 18583 | 4.382413 | 0.000268 |
| C6ORF141 | 2850 | 4.378079 | 0.000272 |
| ALDH3A1 | 827 | 4.377661 | 0.000272 |
| CWC22 | 4671 | 4.377432 | 0.000272 |
| MLNR | 11509 | 4.375747 | 0.000274 |
| C8orf82 | 3001 | 4.372728 | 0.000277 |
| ABHD12B | 87 | 4.372088 | 0.000277 |
| LINC00302 | 9751 | 4.371778 | 0.000277 |
| ABCA17P | 40 | 4.369772 | 0.000279 |
| HTR7 | 8302 | 4.36888 | 0.000279 |
| RGR | 15275 | 4.368467 | 0.000279 |
| DOK6 | 5327 | 4.368181 | 0.000279 |
| MAVS | 11178 | 4.36761 | 0.000279 |
| COL27A1 | 4256 | 4.366298 | 0.000281 |
| C21orf58 | 2637 | 4.365661 | 0.000281 |
| WBSCR27 | 19595 | 4.364066 | 0.000282 |
| TMEM129 | 18294 | 4.352929 | 0.000296 |
| NDRG2 | 12155 | 4.35223 | 0.000297 |
| CYP4F2 | 4799 | 4.342771 | 0.000309 |
| RXFP3 | 15947 | 4.342062 | 0.000309 |
| NEFM | 12222 | 4.339582 | 0.000312 |
| TMEM40 | 18423 | 4.337151 | 0.000315 |
| HOMER1 | 8115 | 4.335485 | 0.000316 |
| AC004863.6 | 129 | 4.335484 | 0.000316 |
| EPHA1 | 5821 | 4.33416 | 0.000317 |
| MYO3A | 11975 | 4.332766 | 0.000319 |
| LRP3 | 10776 | 4.330371 | 0.000321 |
| LRRC24 | 10797 | 4.329215 | 0.000323 |
| DUSP2 | 5453 | 4.32873 | 0.000323 |
| TPBG | 18661 | 4.325227 | 0.000327 |
| PRR5 | 14569 | 4.324707 | 0.000327 |
| GRIN2D | 7562 | 4.322961 | 0.000328 |
| LOC644242 | 10488 | 4.322653 | 0.000328 |
| TRPC6 | 18867 | 4.322576 | 0.000328 |
| PROKR2 | 14524 | 4.319485 | 0.000332 |
| TNP2 | 18594 | 4.319146 | 0.000332 |
| NDST3 | 12160 | 4.317167 | 0.000334 |
| SLC16A3 | 16606 | 4.31664 | 0.000334 |
| OLFM4 | 12780 | 4.316294 | 0.000334 |
| AC005551.1 | 141 | 4.312655 | 0.000339 |
| MT1X | 11792 | 4.309634 | 0.000343 |
| ODF3L2 | 12755 | 4.30745 | 0.000346 |
| GDF5 | 7076 | 4.306989 | 0.000346 |
| LOC100131582 | 9973 | 4.300911 | 0.000355 |
| KIAA1549 | 9114 | 4.29989 | 0.000355 |
| ALPPL2 | 881 | 4.29974 | 0.000355 |
| DHFRP1 | 5091 | 4.298829 | 0.000356 |
| MAPK13 | 11112 | 4.298167 | 0.000356 |
| CDT1 | 3744 | 4.295745 | 0.000359 |
| MBD1 | 11182 | 4.29359 | 0.000362 |
| RAB7L1 | 14932 | 4.292028 | 0.000364 |
| DACT3 | 4839 | 4.290691 | 0.000365 |
| PTP4A1 | 14739 | 4.290507 | 0.000365 |
| SCRG1 | 16129 | 4.288357 | 0.000368 |
| C13orf27 | 2162 | 4.28737 | 0.000369 |
| PSPH | 14684 | 4.287103 | 0.000369 |
| VAMP2 | 19430 | 4.282578 | 0.000375 |
| FAM131C | 6106 | 4.282416 | 0.000375 |
| PF4 | 13694 | 4.282078 | 0.000375 |
| C20orf112 | 2570 | 4.28092 | 0.000375 |
| ETFB | 5928 | 4.280431 | 0.000375 |
| SV2B | 17661 | 4.280372 | 0.000375 |
| AKR1C3 | 780 | 4.279708 | 0.000376 |
| ACTN2 | 511 | 4.277796 | 0.000378 |
| C13orf39 | 2173 | 4.276943 | 0.000379 |
| POLM | 14160 | 4.27139 | 0.000388 |
| FAM102B | 6044 | 4.268464 | 0.000392 |
| ANKRD2 | 978 | 4.267553 | 0.000393 |
| C3orf55 | 2763 | 4.264001 | 0.000399 |
| SCGB3A2 | 16094 | 4.263435 | 0.000399 |
| SKAP1 | 16550 | 4.260205 | 0.000404 |
| WNT10A | 19721 | 4.252971 | 0.000416 |
| FADS2 | 6027 | 4.251412 | 0.000419 |
| SCG3 | 16084 | 4.248867 | 0.000422 |
| NANOS3 | 12049 | 4.246508 | 0.000426 |
| NPPC | 12510 | 4.244456 | 0.000429 |
| LOC90784 | 10707 | 4.243467 | 0.00043 |
| WDR32 | 19618 | 4.242694 | 0.000431 |
| OR4N2 | 13001 | 4.240416 | 0.000435 |
| EYA2 | 5989 | 4.235502 | 0.000443 |
| RP1-163G9.1 | 15522 | 4.235401 | 0.000443 |
| EN2 | 5763 | 4.22935 | 0.000454 |
| MTRR | 11851 | 4.225737 | 0.00046 |
| CTSB | 4630 | 4.224425 | 0.000462 |
| GNB1L | 7288 | 4.224023 | 0.000462 |
| GPR116P2 | 7408 | 4.221986 | 0.000465 |
| LOC729887 | 10679 | 4.221737 | 0.000465 |
| JUN | 8832 | 4.22116 | 0.000466 |
| ADAM33 | 557 | 4.219127 | 0.000469 |
| OR4C15 | 12972 | 4.216961 | 0.000472 |
| TOMM20 | 18615 | 4.216679 | 0.000472 |
| FZD3 | 6883 | 4.215786 | 0.000472 |
| COX6A2 | 4345 | 4.215766 | 0.000472 |
| FAM65B | 6288 | 4.214307 | 0.000474 |
| MSX2P1 | 11783 | 4.214054 | 0.000474 |
| NUDT14 | 12652 | 4.209458 | 0.000483 |
| LINC00086 | 9718 | 4.207987 | 0.000483 |
| RASSF5 | 15064 | 4.20798 | 0.000483 |
| HPGD | 8173 | 4.207953 | 0.000483 |
| ABLIM3 | 106 | 4.207699 | 0.000483 |
| AMDHD1 | 898 | 4.20607 | 0.000486 |
| OR11A1 | 12849 | 4.205795 | 0.000486 |
| OR1N2 | 12898 | 4.205447 | 0.000486 |
| GSTM3 | 7622 | 4.205052 | 0.000486 |
| PET112L | 13677 | 4.203217 | 0.000488 |
| GPR182 | 7455 | 4.202897 | 0.000488 |
| BHLHE23 | 1817 | 4.20136 | 0.000491 |
| RRAGC | 15855 | 4.201102 | 0.000491 |
| KCNH7 | 8898 | 4.19955 | 0.000493 |
| FOXF2 | 6745 | 4.197752 | 0.000496 |
| HSPB8 | 8272 | 4.190139 | 0.000512 |
| THNSL2 | 18112 | 4.188989 | 0.000514 |
| SLC22A12 | 16640 | 4.185609 | 0.00052 |
| LILRA3 | 9685 | 4.185507 | 0.00052 |
| TCF7L1 | 17939 | 4.183449 | 0.000524 |
| AC010896.1 | 191 | 4.181802 | 0.000527 |
| KCNN2 | 8941 | 4.178325 | 0.000533 |
| SHH | 16479 | 4.178189 | 0.000533 |
| IGFALS | 8426 | 4.177684 | 0.000533 |
| INHBB | 8600 | 4.176474 | 0.000535 |
| LOC389458 | 10350 | 4.174599 | 0.000538 |
| CLEC4G | 4087 | 4.174592 | 0.000538 |
| CD4 | 3587 | 4.168964 | 0.00055 |
| SYT4 | 17716 | 4.168167 | 0.000551 |
| CAMK2N2 | 3169 | 4.167526 | 0.000552 |
| DLX5 | 5193 | 4.167056 | 0.000552 |
| THBS4 | 18105 | 4.163401 | 0.00056 |
| PPP2R1B | 14349 | 4.162582 | 0.000561 |
| FAM119A | 6078 | 4.16163 | 0.000562 |
| ONECUT2 | 12794 | 4.160613 | 0.000564 |
| NANP | 12050 | 4.159913 | 0.000564 |
| DBT | 4876 | 4.159668 | 0.000564 |
| KLF8 | 9227 | 4.159407 | 0.000564 |
| BSN | 1940 | 4.15589 | 0.000572 |
| GAS2L1 | 7002 | 4.152934 | 0.000578 |
| ANKRD55 | 1022 | 4.152401 | 0.000579 |
| HMBOX1 | 8041 | 4.150054 | 0.000583 |
| RTN4R | 15914 | 4.149892 | 0.000583 |
| SLC5A5 | 16870 | 4.148489 | 0.000586 |
| XRCC3 | 19801 | 4.146723 | 0.000589 |
| SLC18A3 | 16624 | 4.144332 | 0.000594 |
| ZKSCAN2 | 20051 | 4.141494 | 0.000601 |
| MAFF | 10989 | 4.140635 | 0.000602 |
| DUSP13 | 5447 | 4.137746 | 0.000609 |
| METTL7B | 11354 | 4.136572 | 0.00061 |
| FAM148B | 6123 | 4.136552 | 0.00061 |
| FAM65A | 6287 | 4.136034 | 0.00061 |
| SLC22A13 | 16641 | 4.132011 | 0.00062 |
| SPANXA2-OT1 | 17214 | 4.131147 | 0.000621 |
| XCR1 | 19770 | 4.130053 | 0.000623 |
| NR3C1 | 12547 | 4.129348 | 0.000624 |
| C13orf16 | 2159 | 4.127648 | 0.000627 |
| MUC20 | 11867 | 4.126965 | 0.000628 |
| C1orf69 | 2542 | 4.126234 | 0.000629 |
| SLC25A33 | 16696 | 4.125633 | 0.00063 |
| OSTN | 13227 | 4.123539 | 0.000635 |
| FABP6 | 6022 | 4.121882 | 0.000638 |
| RAB15 | 14876 | 4.117294 | 0.00065 |
| WAC | 19572 | 4.116846 | 0.00065 |
| DRD3 | 5386 | 4.115369 | 0.000652 |
| KIAA1045 | 9072 | 4.115354 | 0.000652 |
| LDHA | 9592 | 4.113644 | 0.000656 |
| ETV5 | 5940 | 4.11271 | 0.000657 |
| SC5DL | 16055 | 4.112627 | 0.000657 |
| FUT1 | 6853 | 4.110814 | 0.000661 |
| LOC440461 | 10422 | 4.110396 | 0.000661 |
| LRFN3 | 10758 | 4.107548 | 0.000668 |
| KCNK5 | 8931 | 4.10594 | 0.000671 |
| SATL1 | 16042 | 4.105537 | 0.000671 |
| C1orf175 | 2493 | 4.105251 | 0.000671 |
| URM1 | 19337 | 4.105104 | 0.000671 |
| EFHB | 5591 | 4.104321 | 0.000672 |
| HHIPL1 | 7899 | 4.096817 | 0.000693 |
| HSPB3 | 8269 | 4.096052 | 0.000694 |
| CTD-2536I1.1 | 4601 | 4.094928 | 0.000696 |
| STYK1 | 17602 | 4.089258 | 0.000711 |
| TUBB2B | 19060 | 4.089235 | 0.000711 |
| TDO2 | 17972 | 4.08877 | 0.000712 |
| PHF10 | 13766 | 4.087432 | 0.000715 |
| FAM113B | 6067 | 4.08616 | 0.000718 |
| OR2T6 | 12954 | 4.083256 | 0.000725 |
| IGSF1 | 8453 | 4.082935 | 0.000725 |
| MTCH2 | 11802 | 4.081515 | 0.000727 |
| SLC6A2 | 16886 | 4.081368 | 0.000727 |
| DENND2D | 5039 | 4.081138 | 0.000727 |
| NXPH2 | 12716 | 4.079108 | 0.000733 |
| DDX52 | 4980 | 4.078415 | 0.000734 |
| CHST8 | 3974 | 4.074794 | 0.000744 |
| LASS6 | 9536 | 4.072026 | 0.000752 |
| XYLT1 | 19814 | 4.071212 | 0.000752 |
| RAPSN | 15025 | 4.070605 | 0.000752 |
| LAX1 | 9541 | 4.070401 | 0.000752 |
| HCN4 | 7801 | 4.070094 | 0.000752 |
| FZD8 | 6888 | 4.070091 | 0.000752 |
| GLI1 | 7204 | 4.068424 | 0.000756 |
| CIDEA | 3991 | 4.068079 | 0.000756 |
| UMOD | 19282 | 4.067137 | 0.000758 |
| PTRHD1 | 14789 | 4.066765 | 0.000758 |
| SMYD2 | 17031 | 4.066278 | 0.000759 |
| ZNF784 | 20535 | 4.064879 | 0.000761 |
| PRR7 | 14571 | 4.064789 | 0.000761 |
| ASB6 | 1410 | 4.064186 | 0.000761 |
| RASGRF2 | 15048 | 4.064057 | 0.000761 |
| CR2 | 4413 | 4.062029 | 0.000767 |
| CPT1A | 4402 | 4.061168 | 0.000768 |
| MRAP2 | 11636 | 4.059103 | 0.000774 |
| RPH3AL | 15711 | 4.054976 | 0.000787 |
| TRIB2 | 18757 | 4.051956 | 0.000796 |
| MYBPC1 | 11909 | 4.051179 | 0.000797 |
| GCC1 | 7043 | 4.04901 | 0.000801 |
| BMP8B | 1861 | 4.048797 | 0.000801 |
| AC005077.3 | 133 | 4.048733 | 0.000801 |
| FDX1L | 6502 | 4.048678 | 0.000801 |
| FAM109A | 6058 | 4.047482 | 0.000804 |
| SYT9 | 17721 | 4.047059 | 0.000804 |
| NME7 | 12410 | 4.044666 | 0.000811 |
| ME1 | 11261 | 4.041393 | 0.00082 |
| FLJ23867 | 6634 | 4.04106 | 0.00082 |
| LOC339352 | 10324 | 4.040818 | 0.00082 |
| KDELR3 | 8987 | 4.040556 | 0.00082 |
| GFI1B | 7100 | 4.03851 | 0.000826 |
| PCDH12 | 13451 | 4.037677 | 0.000828 |
| FAM120AOS | 6081 | 4.036755 | 0.00083 |
| CYorf15B | 4828 | 4.035431 | 0.000834 |
| NINL | 12332 | 4.035044 | 0.000834 |
| SFI1 | 16361 | 4.034034 | 0.000836 |
| EFNB2 | 5602 | 4.032656 | 0.00084 |
| PRDM4 | 14427 | 4.031744 | 0.000841 |
| CCNYL1 | 3507 | 4.031448 | 0.000841 |
| MCTP2 | 11244 | 4.03131 | 0.000841 |
| BIN1 | 1827 | 4.029761 | 0.000845 |
| CYP21A2 | 4763 | 4.026912 | 0.000854 |
| KIAA1377 | 9097 | 4.025608 | 0.000858 |
| RP11-90D4.2 | 15654 | 4.023592 | 0.000864 |
| MXRA5 | 11899 | 4.023117 | 0.000864 |
| ZFR2 | 20024 | 4.021259 | 0.000869 |
| TAOK3 | 17788 | 4.021202 | 0.000869 |
| PTN | 14737 | 4.020634 | 0.00087 |
| SDK1 | 16167 | 4.017378 | 0.00088 |
| LRFN2 | 10757 | 4.015968 | 0.000884 |
| SOD3 | 17145 | 4.01505 | 0.000887 |
| AHNAK2 | 728 | 4.013029 | 0.000891 |
| CABYR | 3100 | 4.012739 | 0.000891 |
| DNAJC15 | 5256 | 4.01246 | 0.000891 |
| SCML1 | 16102 | 4.012156 | 0.000891 |
| CXorf67 | 4726 | 4.012066 | 0.000891 |
| TMEM54 | 18441 | 4.011067 | 0.000894 |
| GLRA3 | 7223 | 4.007852 | 0.000905 |
| NMUR1 | 12420 | 4.007198 | 0.000906 |
| C7orf16 | 2927 | 4.005126 | 0.000913 |
| EGFR | 5614 | 4.004165 | 0.000915 |
| YBX2 | 19821 | 3.999883 | 0.000931 |
| AC063977.1 | 265 | 3.998632 | 0.000934 |
| HAPLN1 | 7743 | 3.997501 | 0.000937 |
| DACH2 | 4836 | 3.99565 | 0.000943 |
| HOXB13 | 8139 | 3.991588 | 0.000958 |
| C17orf96 | 2350 | 3.991005 | 0.000959 |
| HSP90B1 | 8250 | 3.990244 | 0.00096 |
| TPST1 | 18693 | 3.990237 | 0.00096 |
| RXFP1 | 15945 | 3.987856 | 0.000968 |
| MTTP | 11854 | 3.986047 | 0.000974 |
| RHOBTB2 | 15317 | 3.985537 | 0.000975 |
| AARD | 25 | 3.980535 | 0.000994 |
| NDST4 | 12161 | 3.979962 | 0.000995 |
| LINC00334 | 9768 | 3.978589 | 0.001 |
| SLCO4C1 | 16935 | 3.977777 | 0.001 |
| LASS1 | 9531 | 3.977702 | 0.001 |
| FAM70B | 6292 | 3.97726 | 0.001 |
| SNRPD2P1 | 17086 | 3.977188 | 0.001 |
| KANK4 | 8842 | 3.974994 | 0.001007 |
| LOC100286895 | 10061 | 3.974955 | 0.001007 |
| OR51S1 | 13029 | 3.974067 | 0.001008 |
| TRIM10 | 18760 | 3.97402 | 0.001008 |
| GPX3 | 7520 | 3.973622 | 0.001008 |
| VSTM2L | 19551 | 3.973293 | 0.001008 |
| B3GALT2 | 1642 | 3.971667 | 0.001013 |
| NXNL1 | 12712 | 3.968062 | 0.001027 |
| KCNJ16 | 8911 | 3.967934 | 0.001027 |
| ACTN3 | 512 | 3.963368 | 0.001043 |
| ANGPTL4 | 945 | 3.963182 | 0.001043 |
| MTHFD1 | 11815 | 3.963009 | 0.001043 |
| AC034199.2 | 258 | 3.962872 | 0.001043 |
| GEMIN7 | 7093 | 3.962569 | 0.001043 |
| AKAP8 | 767 | 3.961 | 0.001048 |
| LIF | 9678 | 3.957443 | 0.001063 |
| FIGNL2 | 6591 | 3.952417 | 0.001084 |
| CPA5 | 4362 | 3.952107 | 0.001084 |
| EMID1 | 5743 | 3.95123 | 0.001086 |
| KAAG1 | 8836 | 3.950865 | 0.001086 |
| TMTC1 | 18521 | 3.948369 | 0.001096 |
| OR2A14 | 12904 | 3.948184 | 0.001096 |
| MYO5BP2 | 11979 | 3.947294 | 0.001098 |
| C1QL1 | 2432 | 3.945746 | 0.001104 |
| MIF | 11448 | 3.943856 | 0.00111 |
| TMEM63C | 18453 | 3.94375 | 0.00111 |
| FMR1 | 6701 | 3.943524 | 0.00111 |
| SPRN | 17320 | 3.943166 | 0.00111 |
| RHCG | 15309 | 3.942449 | 0.001112 |
| C20orf91 | 2619 | 3.940009 | 0.001122 |
| ASRGL1 | 1446 | 3.938994 | 0.001125 |
| FAM166A | 6151 | 3.938253 | 0.001127 |
| TCTN3 | 17967 | 3.936492 | 0.001134 |
| SRP14P1 | 17382 | 3.933309 | 0.001147 |
| TSPAN2 | 18929 | 3.928016 | 0.001171 |
| C3orf67 | 2769 | 3.927609 | 0.001172 |
| ASB2 | 1406 | 3.926148 | 0.001177 |
| PSMC1 | 14650 | 3.924082 | 0.001186 |
| PCBP3 | 13443 | 3.923105 | 0.001189 |
| GPR32 | 7466 | 3.922462 | 0.001191 |
| CHRDL1 | 3937 | 3.920955 | 0.001196 |
| METRNL | 11337 | 3.920419 | 0.001198 |
| CREM | 4440 | 3.918257 | 0.001207 |
| NR4A2 | 12550 | 3.916405 | 0.001214 |
| C12orf28 | 2113 | 3.915542 | 0.001217 |
| CD28 | 3566 | 3.915135 | 0.001218 |
| WFDC3 | 19692 | 3.914757 | 0.001218 |
| C9orf106 | 3010 | 3.911949 | 0.001231 |
| NR5A2 | 12553 | 3.908855 | 0.001245 |
| LOC642838 | 10464 | 3.903108 | 0.001272 |
| ZNF350 | 20227 | 3.903092 | 0.001272 |
| KLK15 | 9282 | 3.902207 | 0.001275 |
| C6orf129 | 2868 | 3.899591 | 0.001287 |
| RAD51AP2 | 14966 | 3.898659 | 0.00129 |
| MAPK11 | 11110 | 3.898336 | 0.00129 |
| KNG1 | 9305 | 3.898135 | 0.00129 |
| C1orf113 | 2458 | 3.897158 | 0.001293 |
| VDR | 19466 | 3.895952 | 0.001298 |
| NAPRT1 | 12062 | 3.895616 | 0.001298 |
| CNTNAP2 | 4217 | 3.894841 | 0.001301 |
| ALDH9A1 | 836 | 3.892976 | 0.001309 |
| C6orf106 | 2859 | 3.892264 | 0.001311 |
| FAM148C | 6124 | 3.891781 | 0.001312 |
| GABRA3 | 6915 | 3.889127 | 0.001325 |
| CNTN4 | 4213 | 3.888697 | 0.001325 |
| PLA2G7 | 13958 | 3.886694 | 0.001332 |
| TRIM35 | 18783 | 3.886692 | 0.001332 |
| ANKRD28 | 991 | 3.886584 | 0.001332 |
| OR5B17 | 13073 | 3.88233 | 0.001354 |
| TNRC18B | 18599 | 3.881941 | 0.001354 |
| DNAJC27 | 5267 | 3.881498 | 0.001355 |
| ARHGAP26 | 1261 | 3.87954 | 0.001364 |
| PLXDC1 | 14065 | 3.877598 | 0.001373 |
| FABP5 | 6020 | 3.877074 | 0.001375 |
| PPARA | 14257 | 3.874108 | 0.00139 |
| LCE1F | 9557 | 3.871805 | 0.001401 |
| KRTAP6-3 | 9462 | 3.86699 | 0.001427 |
| LINC00087 | 9719 | 3.865235 | 0.001436 |
| CRBN | 4422 | 3.864834 | 0.001436 |
| MT3 | 11794 | 3.864508 | 0.001436 |
| UTS2D | 19422 | 3.863951 | 0.001436 |
| ACAD8 | 416 | 3.863909 | 0.001436 |
| FADS1 | 6026 | 3.859985 | 0.001458 |
| LARS2 | 9528 | 3.859179 | 0.001461 |
| PCDHB6 | 13486 | 3.858325 | 0.001464 |
| GLDC | 7200 | 3.855474 | 0.001479 |
| GUCA2A | 7671 | 3.854809 | 0.001482 |
| GORAB | 7347 | 3.853594 | 0.001487 |
| CCDC168 | 3360 | 3.850848 | 0.001502 |
| VSIG2 | 19544 | 3.84713 | 0.001521 |
| BCORP1 | 1781 | 3.847107 | 0.001521 |
| C11orf86 | 2098 | 3.846622 | 0.001523 |
| PTPN14 | 14752 | 3.84137 | 0.001554 |
| DTL | 5419 | 3.841008 | 0.001554 |
| LMX1B | 9852 | 3.840401 | 0.001556 |
| SLC39A12 | 16818 | 3.838632 | 0.001564 |
| LPAR5 | 10732 | 3.838516 | 0.001564 |
| ACADS | 420 | 3.838303 | 0.001564 |
| MMP23A | 11538 | 3.837966 | 0.001564 |
| LYPD2 | 10939 | 3.836434 | 0.001571 |
| CDO1 | 3733 | 3.836167 | 0.001571 |
| PRM2 | 14502 | 3.834504 | 0.00158 |
| NEURL | 12251 | 3.832331 | 0.001592 |
| BCL2L11 | 1762 | 3.83144 | 0.001596 |
| NRG1 | 12564 | 3.830665 | 0.001599 |
| C22orf34 | 2662 | 3.830459 | 0.001599 |
| PRSS3 | 14590 | 3.826716 | 0.00162 |
| UGT2B10 | 19260 | 3.826652 | 0.00162 |
| ATP6V0A1 | 1556 | 3.825127 | 0.001628 |
| MARVELD3 | 11159 | 3.824628 | 0.001629 |
| VN1R4 | 19499 | 3.823806 | 0.001632 |
| MAPT-AS1 | 11135 | 3.81983 | 0.001657 |
| PDE4C | 13582 | 3.813936 | 0.001695 |
| C16orf3 | 2267 | 3.812747 | 0.001701 |
| HES5 | 7873 | 3.812015 | 0.001704 |
| PRDM10 | 14419 | 3.811702 | 0.001704 |
| SPAM1 | 17212 | 3.81145 | 0.001704 |
| GPR56 | 7479 | 3.810133 | 0.001711 |
| LOC158572 | 10267 | 3.808123 | 0.001723 |
| AGMAT | 696 | 3.806455 | 0.001733 |
| CCBP2 | 3301 | 3.80543 | 0.001738 |
| CIB1 | 3985 | 3.804543 | 0.001742 |
| ILDR1 | 8566 | 3.802981 | 0.001751 |
| TMEM133 | 18302 | 3.802068 | 0.001755 |
| PLXNA1 | 14067 | 3.799968 | 0.001768 |
| HIF3A | 7912 | 3.798845 | 0.001774 |
| CHST7 | 3973 | 3.794742 | 0.001801 |
| THAP8 | 18099 | 3.792949 | 0.001812 |
| DPEP2 | 5339 | 3.791914 | 0.001817 |
| VEGFA | 19467 | 3.791789 | 0.001817 |
| STAT6 | 17513 | 3.789324 | 0.001832 |
| SERF2 | 16277 | 3.788412 | 0.001834 |
| DYRK2 | 5497 | 3.788294 | 0.001834 |
| CDH11 | 3677 | 3.788278 | 0.001834 |
| SCGN | 16096 | 3.787354 | 0.001836 |
| NPW | 12522 | 3.787346 | 0.001836 |
| AGXT | 717 | 3.787069 | 0.001836 |
| SYNPO | 17697 | 3.786715 | 0.001837 |
| FAM179A | 6176 | 3.785876 | 0.001841 |
| SNCG | 17052 | 3.785366 | 0.001842 |
| AGAP3 | 683 | 3.784836 | 0.001844 |
| SOST | 17161 | 3.7828 | 0.001856 |
| PLK3 | 14044 | 3.782511 | 0.001856 |
| CBFA2T3 | 3266 | 3.78235 | 0.001856 |
| CALML3 | 3152 | 3.781602 | 0.00186 |
| GLI3 | 7206 | 3.776177 | 0.001898 |
| AVPR2 | 1626 | 3.773968 | 0.001913 |
| ZNF534 | 20342 | 3.772631 | 0.001921 |
| PPP1R12A | 14316 | 3.772376 | 0.001921 |
| LDHC | 9596 | 3.771983 | 0.001922 |
| FAM86C | 6335 | 3.77121 | 0.001926 |
| ELAC2 | 5699 | 3.770208 | 0.001931 |
| LOC100129726 | 9917 | 3.766102 | 0.001961 |
| ZNF788 | 20539 | 3.764809 | 0.001969 |
| HMGCS2 | 8065 | 3.763469 | 0.001977 |
| CT47A11 | 4575 | 3.76296 | 0.001978 |
| LOC645955 | 10515 | 3.762824 | 0.001978 |
| TMEM105 | 18272 | 3.761964 | 0.001982 |
| B4GALT2 | 1664 | 3.760746 | 0.00199 |
| ZBTB43 | 19903 | 3.758852 | 0.002002 |
| TUBB2C | 19061 | 3.756978 | 0.002015 |
| FXYD7 | 6875 | 3.754995 | 0.002029 |
| FLJ35390 | 6646 | 3.753775 | 0.002037 |
| C6orf222 | 2898 | 3.753243 | 0.002037 |
| ONECUT3 | 12795 | 3.752921 | 0.002037 |
| RFPL4A | 15243 | 3.752657 | 0.002037 |
| DUOXA2 | 5436 | 3.752591 | 0.002037 |
| MAN2A1 | 11051 | 3.751356 | 0.002045 |
| COG3 | 4230 | 3.749607 | 0.002057 |
| COL20A1 | 4250 | 3.748979 | 0.00206 |
| KIAA0748 | 9054 | 3.745443 | 0.002086 |
| MGC23284 | 11403 | 3.74131 | 0.002119 |
| LOC100288282 | 10121 | 3.73981 | 0.002129 |
| ACOT2 | 457 | 3.73856 | 0.002137 |
| HSD11B1L | 8217 | 3.736088 | 0.002156 |
| SERPINI1 | 16322 | 3.733216 | 0.002178 |
| LTK | 10907 | 3.731463 | 0.002191 |
| SLC12A1 | 16579 | 3.730257 | 0.002199 |
| SSTR1 | 17427 | 3.729887 | 0.0022 |
| SMARCC2 | 16984 | 3.729021 | 0.002205 |
| C7orf34 | 2937 | 3.72837 | 0.002208 |
| MEX3A | 11357 | 3.726715 | 0.002218 |
| CHRM4 | 3943 | 3.72669 | 0.002218 |
| OR2V2 | 12956 | 3.724642 | 0.002234 |
| NOV | 12468 | 3.723542 | 0.002241 |
| RP11-332P22.1 | 15575 | 3.721349 | 0.002258 |
| ZNF697 | 20472 | 3.720979 | 0.002259 |
| LOC728636 | 10625 | 3.719814 | 0.002267 |
| PLEKHO2 | 14036 | 3.717156 | 0.002286 |
| LOC100131646 | 9974 | 3.7171 | 0.002286 |
| KRTAP6-2 | 9461 | 3.716649 | 0.002288 |
| TTBK1 | 18958 | 3.715347 | 0.002297 |
| PI3 | 13815 | 3.715089 | 0.002297 |
| KIAA0494 | 9044 | 3.713196 | 0.002312 |
| LRP10 | 10770 | 3.711985 | 0.00232 |
| ANKRD27 | 990 | 3.711669 | 0.002321 |
| MYCN | 11919 | 3.711159 | 0.002323 |
| KRTAP5-6 | 9456 | 3.709407 | 0.002336 |
| AC007743.1 | 155 | 3.70832 | 0.002341 |
| KRTAP20-2 | 9425 | 3.708318 | 0.002341 |
| WSB2 | 19744 | 3.707635 | 0.002345 |
| RP5-1121A15.1 | 15683 | 3.706966 | 0.002349 |
| ADI1 | 625 | 3.705488 | 0.00236 |
| LOC392352 | 10369 | 3.704516 | 0.002364 |
| KCTD15 | 8967 | 3.704473 | 0.002364 |
| RP11-19O2.2 | 15552 | 3.703554 | 0.002368 |
| SLPI | 16960 | 3.703525 | 0.002368 |
| MMP11 | 11527 | 3.700163 | 0.002397 |
| FILIP1L | 6593 | 3.697042 | 0.002424 |
| RLN3 | 15376 | 3.696737 | 0.002424 |
| OR2AE1 | 12911 | 3.695911 | 0.00243 |
| GOLIM4 | 7339 | 3.695172 | 0.002434 |
| CLIC3 | 4097 | 3.693326 | 0.002449 |
| OPTN | 12814 | 3.69306 | 0.002449 |
| TYW3 | 19127 | 3.69178 | 0.002459 |
| XXBAC-BPG308K3.6 | 19810 | 3.690285 | 0.002471 |
| LAMA2 | 9497 | 3.689773 | 0.002473 |
| HADHB | 7733 | 3.68897 | 0.002478 |
| CLCF1 | 4032 | 3.68721 | 0.002493 |
| CUL2 | 4658 | 3.685813 | 0.002504 |
| C7orf62 | 2959 | 3.682906 | 0.00253 |
| CREG2 | 4437 | 3.680461 | 0.002551 |
| PAH | 13331 | 3.678729 | 0.002566 |
| TRIML2 | 18831 | 3.677587 | 0.002575 |
| OR6C74 | 13121 | 3.676926 | 0.002576 |
| DPRXP4 | 5363 | 3.676912 | 0.002576 |
| ADAMTSL3 | 585 | 3.672495 | 0.002616 |
| AC010606.4 | 188 | 3.672474 | 0.002616 |
| WFDC10B | 19687 | 3.66992 | 0.002639 |
| VGF | 19475 | 3.668697 | 0.002648 |
| TOLLIP | 18611 | 3.668534 | 0.002648 |
| IFI35 | 8369 | 3.667899 | 0.002649 |
| TMPO | 18498 | 3.667877 | 0.002649 |
| SPPL2B | 17315 | 3.667116 | 0.002654 |
| SSR3 | 17421 | 3.666808 | 0.002654 |
| PPP1R3C | 14337 | 3.666643 | 0.002654 |
| ID2 | 8341 | 3.665375 | 0.002664 |
| FGFRL1 | 6566 | 3.665031 | 0.002665 |
| GLT1D1 | 7232 | 3.662401 | 0.00269 |
| OR10G2 | 12824 | 3.662155 | 0.00269 |
| CSNK1A1P1 | 4534 | 3.661411 | 0.002695 |
| PAGE1 | 13325 | 3.66027 | 0.002704 |
| GPA33 | 7361 | 3.659797 | 0.002706 |
| WDR86 | 19672 | 3.65774 | 0.002723 |
| SLC24A6 | 16669 | 3.657682 | 0.002723 |
| LOC643085 | 10470 | 3.656558 | 0.002732 |
| PRSS2 | 14585 | 3.656039 | 0.002735 |
| MEGF8 | 11308 | 3.654175 | 0.00275 |
| NCR3 | 12139 | 3.654032 | 0.00275 |
| FSTL4 | 6827 | 3.651832 | 0.002771 |
| MAPKAPK3 | 11127 | 3.650738 | 0.00278 |
| ST8SIA6 | 17478 | 3.648852 | 0.002798 |
| DPYS | 5374 | 3.648481 | 0.002799 |
| ZNF319 | 20201 | 3.647672 | 0.002805 |
| TAOK1 | 17786 | 3.646603 | 0.002814 |
| SATB1 | 16040 | 3.64616 | 0.002816 |
| GRAMD1C | 7528 | 3.645653 | 0.002818 |
| ACOT8 | 461 | 3.644779 | 0.002825 |
| PLEKHM2 | 14032 | 3.642942 | 0.002842 |
| GALM | 6959 | 3.638838 | 0.002885 |
| GRIN2C | 7561 | 3.637042 | 0.002902 |
| ZBTB9 | 19918 | 3.635583 | 0.002916 |
| LRRTM4 | 10870 | 3.635209 | 0.002917 |
| COL10A1 | 4237 | 3.633807 | 0.002929 |
| FSCN2 | 6815 | 3.633639 | 0.002929 |
| OSBPL1A | 13205 | 3.632777 | 0.002932 |
| KIAA0101 | 9020 | 3.632633 | 0.002932 |
| ITGAL | 8749 | 3.632559 | 0.002932 |
| KYNU | 9478 | 3.630679 | 0.002951 |
| SPRED3 | 17319 | 3.630173 | 0.002953 |
| SNX26 | 17118 | 3.629512 | 0.002958 |
| CCL22 | 3465 | 3.627744 | 0.002975 |
| ZFHX2 | 19996 | 3.625988 | 0.002993 |
| CCK | 3450 | 3.624816 | 0.003003 |
| ORMDL3 | 13199 | 3.624295 | 0.003005 |
| C12orf64 | 2145 | 3.624165 | 0.003005 |
| FAM149B1 | 6126 | 3.6238 | 0.003006 |
| HS3ST2 | 8205 | 3.623153 | 0.003011 |
| HIST1H3H | 7972 | 3.622662 | 0.003013 |
| DUSP6 | 5463 | 3.620601 | 0.003034 |
| SLC13A5 | 16592 | 3.618475 | 0.003056 |
| TSC1 | 18888 | 3.617628 | 0.003063 |
| PTPRF | 14771 | 3.616961 | 0.003068 |
| NCOA7 | 12133 | 3.614101 | 0.003099 |
| PRRG3 | 14575 | 3.613793 | 0.0031 |
| ZNF124 | 20083 | 3.613084 | 0.003105 |
| PROKR1 | 14523 | 3.611802 | 0.003117 |
| TMEFF2 | 18267 | 3.610531 | 0.00313 |
| CPXM2 | 4409 | 3.609174 | 0.003143 |
| FAM123B | 6088 | 3.608266 | 0.003151 |
| KRTAP10-9 | 9401 | 3.606051 | 0.003175 |
| CRCT1 | 4424 | 3.600356 | 0.003242 |
| CCBE1 | 3298 | 3.598858 | 0.003255 |
| MOSPD3 | 11598 | 3.598785 | 0.003255 |
| KIAA1586 | 9115 | 3.595975 | 0.003287 |
| RAX2 | 15072 | 3.594647 | 0.003301 |
| CCIN | 3449 | 3.594225 | 0.003303 |
| CA5BP | 3084 | 3.593918 | 0.003303 |
| LRRC32 | 10806 | 3.59333 | 0.003306 |
| A2ML1 | 8 | 3.593151 | 0.003306 |
| LOC730144 | 10685 | 3.592911 | 0.003306 |
| EFEMP2 | 5588 | 3.592375 | 0.00331 |
| RNF113B | 15411 | 3.589981 | 0.003337 |
| RHOD | 15320 | 3.589512 | 0.00334 |
| HTR3C | 8296 | 3.587908 | 0.003357 |
| CHEK1 | 3900 | 3.586287 | 0.003375 |
| SPRR3 | 17329 | 3.584558 | 0.003394 |
| C8orf46 | 2989 | 3.584327 | 0.003394 |
| IAPP | 8323 | 3.58223 | 0.003418 |
| AP1M2 | 1119 | 3.580765 | 0.003434 |
| NLK | 12381 | 3.57984 | 0.003443 |
| ELP3 | 5732 | 3.579022 | 0.00345 |
| ATBF1 | 1464 | 3.576853 | 0.003475 |
| MYO5B | 11978 | 3.575973 | 0.003484 |
| EFNA5 | 5600 | 3.573455 | 0.003514 |
| LOC652614 | 10568 | 3.572982 | 0.003517 |
| TSPAN18 | 18927 | 3.570525 | 0.003544 |
| ALK | 855 | 3.57047 | 0.003544 |
| AC138472.2 | 393 | 3.568876 | 0.003561 |
| IER5L | 8361 | 3.568747 | 0.003561 |
| ZP2 | 20605 | 3.567901 | 0.003569 |
| PRLH | 14498 | 3.561425 | 0.003654 |
| EBPL | 5529 | 3.559297 | 0.003681 |
| LOC100131960 | 9989 | 3.558604 | 0.003687 |
| LOC100287347 | 10079 | 3.558269 | 0.003688 |
| KBTBD11 | 8853 | 3.557622 | 0.003692 |
| CTNND2 | 4620 | 3.5575 | 0.003692 |
| B3GNT7 | 1655 | 3.556556 | 0.003701 |
| KLF10 | 9212 | 3.556285 | 0.003702 |
| AC139099.1 | 396 | 3.553114 | 0.003743 |
| FERD3L | 6515 | 3.552875 | 0.003743 |
| HOXD9 | 8166 | 3.552203 | 0.003749 |
| AC112641.2 | 355 | 3.550936 | 0.003763 |
| PNPLA7 | 14126 | 3.550378 | 0.003768 |
| C17orf39 | 2315 | 3.549794 | 0.003773 |
| TRIM6 | 18810 | 3.547092 | 0.003808 |
| HSPA9 | 8265 | 3.545388 | 0.003824 |
| LPP | 10746 | 3.545384 | 0.003824 |
| AKAP12 | 759 | 3.545216 | 0.003824 |
| FOXM1 | 6758 | 3.544682 | 0.003827 |
| CYP4Z1 | 4808 | 3.544516 | 0.003827 |
| WNT6 | 19732 | 3.544273 | 0.003827 |
| S1PR2 | 15980 | 3.54398 | 0.003828 |
| NTN3 | 12622 | 3.543073 | 0.003837 |
| MSX1 | 11781 | 3.54255 | 0.003841 |
| ATP6V1C1 | 1568 | 3.54193 | 0.003847 |
| ITGB1BP1 | 8754 | 3.539084 | 0.003885 |
| PHLPP2 | 13800 | 3.53858 | 0.003889 |
| HTR3B | 8295 | 3.536936 | 0.003909 |
| KCNJ3 | 8914 | 3.535818 | 0.003922 |
| POLR2H | 14175 | 3.533076 | 0.003959 |
| ATG5 | 1488 | 3.531485 | 0.003979 |
| SYCN | 17672 | 3.529793 | 0.004001 |
| DACH1 | 4835 | 3.529542 | 0.004001 |
| CXorf1 | 4702 | 3.528333 | 0.004016 |
| SVOP | 17666 | 3.526124 | 0.004046 |
| TMEM95 | 18485 | 3.524014 | 0.004074 |
| LOC643421 | 10473 | 3.523806 | 0.004074 |
| LOC100131504 | 9967 | 3.522837 | 0.004082 |
| PCDHB8 | 13488 | 3.522725 | 0.004082 |
| MSLN | 11767 | 3.522508 | 0.004082 |
| CDX1 | 3746 | 3.521602 | 0.004093 |
| OR2T33 | 12950 | 3.520085 | 0.004112 |
| BCKDK | 1754 | 3.519211 | 0.004118 |
| PCYOX1L | 13550 | 3.519207 | 0.004118 |
| BDH2 | 1785 | 3.517462 | 0.004142 |
| LOC729680 | 10668 | 3.517147 | 0.004143 |
| PROS1 | 14529 | 3.516319 | 0.004152 |
| HBE1 | 7772 | 3.515776 | 0.004157 |
| LSS | 10893 | 3.510541 | 0.004235 |
| GABRA1 | 6913 | 3.509118 | 0.004251 |
| STEAP1 | 17520 | 3.5091 | 0.004251 |
| WNT11 | 19723 | 3.50843 | 0.004254 |
| FMN1 | 6689 | 3.508399 | 0.004254 |
| ALDH2 | 826 | 3.507806 | 0.00426 |
| NFE2L1 | 12276 | 3.507554 | 0.00426 |
| C6orf1 | 2854 | 3.506933 | 0.004266 |
| LAMB1 | 9501 | 3.504526 | 0.004301 |
| NRIP2 | 12570 | 3.503429 | 0.004315 |
| CST5 | 4559 | 3.503143 | 0.004315 |
| LOC648603 | 10548 | 3.501763 | 0.004334 |
| TMEM52 | 18439 | 3.500897 | 0.004344 |
| LOC644662 | 10495 | 3.499547 | 0.004362 |
| TYRO3 | 19121 | 3.499186 | 0.004364 |
| LOC554207 | 10455 | 3.498606 | 0.004369 |
| OR2B2 | 12917 | 3.496851 | 0.004392 |
| CDH8 | 3697 | 3.496758 | 0.004392 |
| RASSF10 | 15060 | 3.493951 | 0.004434 |
| HSF2BP | 8240 | 3.492785 | 0.004449 |
| CXCR3 | 4693 | 3.492583 | 0.004449 |
| ULBP1 | 19275 | 3.490834 | 0.004474 |
| RP5-1068E13.3 | 15682 | 3.489622 | 0.004491 |
| ZBBX | 19872 | 3.488139 | 0.00451 |
| TCFL5 | 17941 | 3.487977 | 0.00451 |
| TCTA | 17959 | 3.487616 | 0.004512 |
| TOMM40L | 18620 | 3.487241 | 0.004515 |
| BCORL1 | 1780 | 3.485789 | 0.004535 |
| HIST1H3D | 7968 | 3.483194 | 0.004575 |
| PROCA1 | 14517 | 3.480704 | 0.004605 |
| WDR31 | 19617 | 3.480675 | 0.004605 |
| FOXE1 | 6742 | 3.480653 | 0.004605 |
| TUFM | 19074 | 3.480501 | 0.004605 |
| STX12 | 17581 | 3.480079 | 0.004608 |
| NUAK1 | 12634 | 3.479517 | 0.004614 |
| PRKDC | 14489 | 3.477627 | 0.00464 |
| TUBB1 | 19058 | 3.47752 | 0.00464 |
| ZBTB45 | 19905 | 3.476893 | 0.004643 |
| DDR2 | 4946 | 3.476876 | 0.004643 |
| PXN | 14826 | 3.474524 | 0.00468 |
| NUFIP2 | 12673 | 3.474137 | 0.004682 |
| GPX4 | 7521 | 3.470656 | 0.004739 |
| F7 | 6008 | 3.467738 | 0.004787 |
| PRSS33 | 14591 | 3.46486 | 0.004834 |
| LY6K | 10926 | 3.464526 | 0.004836 |
| SOX13 | 17167 | 3.463743 | 0.004845 |
| KRT9 | 9385 | 3.462324 | 0.004866 |
| GJC1 | 7180 | 3.462151 | 0.004866 |
| OTUD7A | 13246 | 3.461612 | 0.004871 |
| E2F3 | 5509 | 3.460617 | 0.004885 |
| CIB2 | 3986 | 3.458826 | 0.004913 |
| KCNV1 | 8958 | 3.457052 | 0.004941 |
| HES7 | 7875 | 3.456711 | 0.004943 |
| GKN1 | 7191 | 3.456209 | 0.004948 |
| TRAPPC9 | 18736 | 3.455739 | 0.00495 |
| ITGA5 | 8742 | 3.45546 | 0.00495 |
| OR2M1P | 12937 | 3.455369 | 0.00495 |
| FAM69B | 6290 | 3.455059 | 0.004952 |
| PEX10 | 13679 | 3.454248 | 0.004962 |
| PFN1P2 | 13710 | 3.453574 | 0.00497 |
| SLC6A12 | 16878 | 3.452968 | 0.004977 |
| RAB31 | 14895 | 3.452689 | 0.004978 |
| ETV3 | 5937 | 3.451039 | 0.005004 |
| CRH | 4441 | 3.450083 | 0.005018 |
| C2orf83 | 2723 | 3.447951 | 0.005053 |
| NETO2 | 12246 | 3.44633 | 0.005079 |
| F2RL3 | 6005 | 3.445982 | 0.005081 |
| ATCAY | 1465 | 3.445745 | 0.005081 |
| DUX4L15 | 5470 | 3.443874 | 0.005112 |
| VTI1B | 19558 | 3.442897 | 0.005126 |
| TMEM155 | 18326 | 3.441784 | 0.005139 |
| SOCS1 | 17136 | 3.441747 | 0.005139 |
| FKBP4 | 6608 | 3.441385 | 0.005142 |
| FAM167B | 6154 | 3.440627 | 0.005152 |
| MGLL | 11420 | 3.440096 | 0.005157 |
| CRIP2 | 4447 | 3.43975 | 0.00516 |
| SYNPO2L | 17699 | 3.439424 | 0.005161 |
| METAP1 | 11333 | 3.438599 | 0.005173 |
| ATXN7L2 | 1609 | 3.4376 | 0.005187 |
| TRIM47 | 18797 | 3.43732 | 0.005188 |
| SOX11 | 17165 | 3.437018 | 0.00519 |
| ZEB1 | 19985 | 3.436143 | 0.005202 |
| LRRN2 | 10863 | 3.435589 | 0.005206 |
| NKD2 | 12355 | 3.435462 | 0.005206 |
| OR1F2P | 12884 | 3.4352 | 0.005207 |
| ACOX2 | 464 | 3.434838 | 0.005209 |
| PDK3 | 13618 | 3.433932 | 0.005222 |
| KRT17 | 9334 | 3.433405 | 0.005228 |
| CACNA1I | 3110 | 3.432743 | 0.005236 |
| API5L1 | 1159 | 3.431623 | 0.00525 |
| ZC2HC1A | 19919 | 3.43155 | 0.00525 |
| ACSS1 | 493 | 3.430887 | 0.005257 |
| PINX1 | 13881 | 3.430652 | 0.005257 |
| RPH3A | 15710 | 3.430535 | 0.005257 |
| SLC2A12 | 16743 | 3.42995 | 0.005264 |
| HMGB3P27 | 8057 | 3.428958 | 0.005279 |
| CBL | 3268 | 3.428091 | 0.005287 |
| CD83 | 3614 | 3.428078 | 0.005287 |
| TMEM132B | 18298 | 3.427365 | 0.005296 |
| PCBD1 | 13438 | 3.426349 | 0.005312 |
| ZNF804B | 20551 | 3.423966 | 0.005353 |
| SSTR2 | 17428 | 3.423751 | 0.005353 |
| LCE1C | 9554 | 3.423568 | 0.005353 |
| SWAP70 | 17668 | 3.422439 | 0.00537 |
| RAB14 | 14875 | 3.421553 | 0.005383 |
| NRD1 | 12562 | 3.421179 | 0.005386 |
| LCE3E | 9566 | 3.418649 | 0.005432 |
| LOC401052 | 10391 | 3.417768 | 0.005445 |
| GLIPR1L2 | 7209 | 3.417152 | 0.005453 |
| SOX12 | 17166 | 3.416157 | 0.005468 |
| OR6K2 | 13125 | 3.415213 | 0.005483 |
| LOC100132483 | 10007 | 3.412192 | 0.005536 |
| TBC1D20 | 17849 | 3.412129 | 0.005536 |
| FAM110C | 6062 | 3.411253 | 0.005549 |
| LAMB3 | 9504 | 3.4091 | 0.005589 |
| RGL1 | 15264 | 3.405791 | 0.005646 |
| C17orf82 | 2345 | 3.40578 | 0.005646 |
| SCGB3A1 | 16093 | 3.405652 | 0.005646 |
| MAP4 | 11095 | 3.404789 | 0.005654 |
| C1orf144 | 2477 | 3.404779 | 0.005654 |
| LINC00473 | 9783 | 3.404172 | 0.005662 |
| HIST1H3J | 7974 | 3.403178 | 0.005675 |
| AP001053.11 | 1095 | 3.403102 | 0.005675 |
| HBS1L | 7779 | 3.401794 | 0.005697 |
| LOR | 10719 | 3.400153 | 0.005727 |
| PRKCG | 14480 | 3.399099 | 0.005744 |
| MPPED1 | 11623 | 3.398275 | 0.005757 |
| C11orf92 | 2102 | 3.397832 | 0.005762 |
| EMX2OS | 5761 | 3.397225 | 0.00577 |
| HCFC1R1 | 7786 | 3.395799 | 0.005795 |
| SCUBE1 | 16138 | 3.395319 | 0.005801 |
| PMP2 | 14087 | 3.395057 | 0.005801 |
| LOC100292160 | 10213 | 3.394787 | 0.005802 |
| ENTPD2 | 5792 | 3.39441 | 0.005805 |
| GPR157 | 7438 | 3.394213 | 0.005805 |
| CCDC88C | 3436 | 3.393336 | 0.005818 |
| VILL | 19484 | 3.393058 | 0.005818 |
| LOC100130360 | 9935 | 3.392935 | 0.005818 |
| TMEM184B | 18359 | 3.392239 | 0.005828 |
| GJB5 | 7177 | 3.389172 | 0.005889 |
| RHEBL1 | 15312 | 3.386144 | 0.005949 |
| BEST3 | 1800 | 3.384036 | 0.00599 |
| ADAT3 | 596 | 3.383678 | 0.005993 |
| MSI2 | 11762 | 3.382567 | 0.006013 |
| QPRT | 14850 | 3.382263 | 0.006014 |
| FAM59A | 6279 | 3.381086 | 0.006035 |
| C6orf27 | 2904 | 3.379093 | 0.006074 |
| TMEM150B | 18321 | 3.376355 | 0.00613 |
| FBXO28 | 6434 | 3.374317 | 0.006171 |
| CR1 | 4411 | 3.373565 | 0.006183 |
| GJD4 | 7185 | 3.372939 | 0.006192 |
| KCNG2 | 8889 | 3.371165 | 0.006227 |
| GALNT1 | 6961 | 3.370242 | 0.006242 |
| C22orf36 | 2663 | 3.369814 | 0.006247 |
| LCMT2 | 9573 | 3.368127 | 0.006277 |
| CCDC90A | 3439 | 3.368053 | 0.006277 |
| MBOAT2 | 11199 | 3.367343 | 0.006288 |
| LOC100130935 | 9944 | 3.365148 | 0.006333 |
| SLC35F2 | 16791 | 3.364576 | 0.006341 |
| AC068658.1 | 274 | 3.364299 | 0.006343 |
| S100A8 | 15972 | 3.363455 | 0.006357 |
| MAP1LC3B | 11066 | 3.362401 | 0.006374 |
| PRAF2 | 14390 | 3.362294 | 0.006374 |
| RXRA | 15949 | 3.361785 | 0.00638 |
| TMEM14E | 18319 | 3.360698 | 0.006398 |
| SLC39A2 | 16821 | 3.360582 | 0.006398 |
| DZIP3 | 5506 | 3.359921 | 0.006408 |
| RIMBP3 | 15348 | 3.359459 | 0.006414 |
| LOC100129272 | 9900 | 3.357247 | 0.00646 |
| TMIE | 18491 | 3.355794 | 0.006489 |
| SDCCAG10 | 16153 | 3.354329 | 0.006518 |
| GH2 | 7133 | 3.354057 | 0.00652 |
| CNR1 | 4202 | 3.35375 | 0.006522 |
| KRTAP10-10 | 9391 | 3.350467 | 0.006594 |
| ST3GAL1 | 17454 | 3.348694 | 0.006631 |
| SNX21 | 17114 | 3.348349 | 0.006634 |
| ASS1 | 1447 | 3.347832 | 0.006638 |
| C21ORF119 | 2622 | 3.347745 | 0.006638 |
| PYDC1 | 14832 | 3.347316 | 0.006643 |
| NKX1-2 | 12362 | 3.346462 | 0.006652 |
| RG9MTD1 | 15259 | 3.346414 | 0.006652 |
| DCAF7 | 4886 | 3.346318 | 0.006652 |
| SLMAP | 16956 | 3.346 | 0.006654 |
| MIR100HG | 11460 | 3.345429 | 0.006662 |
| LOC389831 | 10355 | 3.338861 | 0.006817 |
| GPR160 | 7440 | 3.336873 | 0.00686 |
| PRTG | 14602 | 3.336648 | 0.00686 |
| MFGE8 | 11368 | 3.335019 | 0.006895 |
| MURC | 11883 | 3.333531 | 0.006927 |
| ACPT | 473 | 3.332753 | 0.006941 |
| ROBO3 | 15502 | 3.331574 | 0.006965 |
| PRPH | 14547 | 3.331299 | 0.006966 |
| SMAD3 | 16967 | 3.33098 | 0.006969 |
| STX1A | 17586 | 3.330415 | 0.006978 |
| HMMR | 8076 | 3.32994 | 0.006984 |
| CRYBB3 | 4490 | 3.329288 | 0.006995 |
| RAPGEF3 | 15019 | 3.32666 | 0.007054 |
| FGD1 | 6529 | 3.326501 | 0.007054 |
| RTN4IP1 | 15913 | 3.325812 | 0.007061 |
| LOC100287146 | 10072 | 3.325792 | 0.007061 |
| UCP3 | 19242 | 3.324283 | 0.007094 |
| LRRC62 | 10840 | 3.3216 | 0.007157 |
| TAF3 | 17762 | 3.320743 | 0.007174 |
| UNC5B | 19295 | 3.319107 | 0.00721 |
| FTL | 6834 | 3.318477 | 0.007221 |
| MYEOV2 | 11925 | 3.318268 | 0.007221 |
| FBXO41 | 6446 | 3.317019 | 0.007247 |
| C14orf43 | 2219 | 3.316601 | 0.007253 |
| COMP | 4294 | 3.316127 | 0.007259 |
| PDIA4 | 13611 | 3.313689 | 0.007316 |
| CORT | 4330 | 3.313539 | 0.007316 |
| LCNL1 | 9583 | 3.311381 | 0.007361 |
| ST8SIA5 | 17477 | 3.311366 | 0.007361 |
| IFT57 | 8410 | 3.311026 | 0.007365 |
| CGB2 | 3864 | 3.310633 | 0.007367 |
| C12orf5 | 2132 | 3.31052 | 0.007367 |
| DLEU1 | 5170 | 3.307708 | 0.007435 |
| KCNA10 | 8863 | 3.306933 | 0.00745 |
| MMEL1 | 11523 | 3.306423 | 0.007458 |
| NKX2-3 | 12365 | 3.305655 | 0.007473 |
| AC120024.1 | 365 | 3.305282 | 0.007477 |
| LMO3 | 9843 | 3.304336 | 0.007497 |
| CNIH3 | 4175 | 3.303465 | 0.007514 |
| SLFNL1 | 16945 | 3.303207 | 0.007515 |
| FAM48B1 | 6261 | 3.302467 | 0.00753 |
| ANKRD22 | 986 | 3.300812 | 0.007568 |
| CYP17A1 | 4756 | 3.300252 | 0.007574 |
| LOC100289143 | 10158 | 3.300193 | 0.007574 |
| SGTB | 16423 | 3.299892 | 0.007576 |
| C2orf70 | 2712 | 3.299658 | 0.007577 |
| STK17A | 17532 | 3.297986 | 0.007616 |
| BMPR2 | 1865 | 3.296662 | 0.007646 |
| KCNK1 | 8920 | 3.295701 | 0.007667 |
| C1S | 2446 | 3.295167 | 0.007672 |
| OMP | 12792 | 3.295089 | 0.007672 |
| LOC344593 | 10335 | 3.29299 | 0.007723 |
| GULP1 | 7683 | 3.291242 | 0.007762 |
| NPY5R | 12526 | 3.291155 | 0.007762 |
| RCN1 | 15162 | 3.283631 | 0.007966 |
| INGX | 8597 | 3.283321 | 0.007969 |
| WNT7B | 19734 | 3.283116 | 0.007969 |
| ADAMTS18 | 572 | 3.282653 | 0.007976 |
| ARPC3P3 | 1355 | 3.281695 | 0.007992 |
| HCG8 | 7794 | 3.281657 | 0.007992 |
| AC010336.2 | 185 | 3.280787 | 0.008011 |
| AHSG | 732 | 3.280463 | 0.008014 |
| KCNH5 | 8896 | 3.280128 | 0.008018 |
| FTHL17 | 6833 | 3.278594 | 0.008055 |
| TXNRD3IT1 | 19116 | 3.278161 | 0.008062 |
| CRYBB2 | 4488 | 3.27773 | 0.008068 |
| LOC100290566 | 10199 | 3.277038 | 0.008082 |
| AP2A2 | 1124 | 3.274306 | 0.008154 |
| C14orf37 | 2216 | 3.272022 | 0.008214 |
| MUC4 | 11872 | 3.271829 | 0.008214 |
| ST20 | 17453 | 3.271449 | 0.008219 |
| NR4A3 | 12551 | 3.269739 | 0.008262 |
| VAT1 | 19445 | 3.269362 | 0.008267 |
| PTPN11 | 14749 | 3.269019 | 0.008271 |
| SAMD10 | 16002 | 3.268679 | 0.008271 |
| ACAA2 | 411 | 3.268597 | 0.008271 |
| SYT1 | 17705 | 3.267711 | 0.008285 |
| PDLIM7 | 13625 | 3.26769 | 0.008285 |
| HSP90AA2 | 8248 | 3.265172 | 0.008353 |
| NDP | 12153 | 3.26405 | 0.00838 |
| LRRC63 | 10841 | 3.263562 | 0.008386 |
| TMEM150C | 18322 | 3.263426 | 0.008386 |
| ACR | 474 | 3.258615 | 0.008516 |
| RIOK1 | 15359 | 3.258375 | 0.008516 |
| INO80B | 8605 | 3.258268 | 0.008516 |
| USP35 | 19380 | 3.258225 | 0.008516 |
| PAPLN | 13362 | 3.257082 | 0.008545 |
| MARCKSL1 | 11149 | 3.255342 | 0.008591 |
| CDC42EP5 | 3658 | 3.254322 | 0.008615 |
| BLK | 1838 | 3.252562 | 0.008662 |
| TAX1BP1 | 17834 | 3.252026 | 0.008672 |
| TRIM66 | 18818 | 3.251077 | 0.008695 |
| FLJ39653 | 6657 | 3.249892 | 0.008725 |
| PTCRA | 14702 | 3.249107 | 0.008743 |
| CD79A | 3609 | 3.248118 | 0.008764 |
| RRP7A | 15872 | 3.247986 | 0.008764 |
| TMX2 | 18528 | 3.246577 | 0.008801 |
| CILP | 3996 | 3.244681 | 0.008849 |
| CAPN12 | 3190 | 3.244484 | 0.008849 |
| SMURF1 | 17028 | 3.244427 | 0.008849 |
| DGCR14 | 5068 | 3.243697 | 0.008865 |
| SERPINE2 | 16316 | 3.242389 | 0.008899 |
| PRAMEF23 | 14402 | 3.241988 | 0.008905 |
| RALBP1 | 14990 | 3.241438 | 0.008916 |
| PRIM2 | 14458 | 3.240756 | 0.008931 |
| KIF21A | 9163 | 3.240185 | 0.008937 |
| CHDH | 3899 | 3.240142 | 0.008937 |
| INE1 | 8590 | 3.238093 | 0.008995 |
| TTC38 | 18989 | 3.237639 | 0.009003 |
| GPRIN2 | 7508 | 3.237135 | 0.009011 |
| RUSC1-AS1 | 15936 | 3.236967 | 0.009011 |
| CD300C | 3570 | 3.235569 | 0.009049 |
| ZNF624 | 20419 | 3.234541 | 0.009067 |
| LOC392288 | 10368 | 3.234416 | 0.009067 |
| ATP1A2 | 1516 | 3.234301 | 0.009067 |
| PARP1 | 13390 | 3.234189 | 0.009067 |
| GPR176 | 7450 | 3.232664 | 0.009108 |
| MFSD7 | 11384 | 3.232483 | 0.009108 |
| CCDC40 | 3380 | 3.232188 | 0.009111 |
| MCTP1 | 11243 | 3.230556 | 0.009155 |
| ZNF703 | 20479 | 3.230395 | 0.009155 |
| LOC100130344 | 9933 | 3.229524 | 0.009176 |
| C9orf23 | 3033 | 3.229003 | 0.009182 |
| AAGAB | 21 | 3.228881 | 0.009182 |
| TNFRSF8 | 18560 | 3.228713 | 0.009182 |
| GSX2 | 7633 | 3.228148 | 0.009194 |
| THEMIS | 18109 | 3.225404 | 0.009276 |
| TMEM181 | 18355 | 3.225121 | 0.009277 |
| ONECUT1 | 12793 | 3.224955 | 0.009277 |
| MAOB | 11062 | 3.224222 | 0.009294 |
| FKSG2 | 6617 | 3.22387 | 0.009294 |
| STYXL1 | 17604 | 3.223822 | 0.009294 |
| BATF3 | 1718 | 3.222783 | 0.009321 |
| KIAA1958 | 9139 | 3.220126 | 0.009402 |
| SPR | 17316 | 3.218481 | 0.009449 |
| GBA3 | 7025 | 3.217299 | 0.009478 |
| LARP4 | 9523 | 3.217181 | 0.009478 |
| ATP8B1 | 1582 | 3.216451 | 0.009496 |
| FAM21C | 6218 | 3.215333 | 0.009526 |
| PHLDB2 | 13797 | 3.214052 | 0.009562 |
| PNMAL2 | 14114 | 3.213528 | 0.009562 |
| Sep-11 | 16258 | 3.213493 | 0.009562 |
| LOC100289760 | 10182 | 3.213427 | 0.009562 |
| SYT12 | 17708 | 3.213232 | 0.009562 |
| KCNQ4 | 8949 | 3.211687 | 0.009607 |
| WBP5 | 19591 | 3.209915 | 0.00966 |
| OSBPL8 | 13211 | 3.208249 | 0.009709 |
| PDX1 | 13636 | 3.207628 | 0.009723 |
| PFKL | 13706 | 3.206389 | 0.009758 |
| RGS4 | 15290 | 3.20584 | 0.00977 |
| ZNF365 | 20233 | 3.205335 | 0.009778 |
| TNMD | 18582 | 3.205201 | 0.009778 |
| HIST1H4J | 7984 | 3.204162 | 0.009806 |
| RAB2B | 14893 | 3.203267 | 0.00983 |
| RP1-177G6.1 | 15524 | 3.202252 | 0.009858 |
| CXCR5 | 4695 | 3.201644 | 0.009867 |
| LILRA1 | 9683 | 3.201567 | 0.009867 |
| DCI | 4898 | 3.198892 | 0.009952 |
| GPSM2 | 7513 | 3.19826 | 0.009967 |
| LILRA6 | 9688 | 3.197415 | 0.00999 |
| KLK10 | 9277 | 3.197185 | 0.009991 |
| RIPK4 | 15365 | 3.196976 | 0.009991 |
| TSPAN31 | 18931 | 3.196764 | 0.009991 |
| TAL1 | 17780 | 3.1953 | 0.010028 |
| BEND4 | 1794 | 3.195298 | 0.010028 |
| CNDP2 | 4165 | 3.194218 | 0.010059 |
| RAPH1 | 15024 | 3.191707 | 0.01014 |
| FAM84B | 6332 | 3.189581 | 0.010207 |
| C10orf131 | 2015 | 3.189277 | 0.010211 |
| BAT2D1 | 1711 | 3.18854 | 0.01023 |
| ZNF28 | 20177 | 3.187795 | 0.010247 |
| DYNC1LI1 | 5485 | 3.18765 | 0.010247 |
| SYT17 | 17713 | 3.18624 | 0.01029 |
| FOXD3 | 6738 | 3.184869 | 0.010332 |
| SULF2 | 17616 | 3.181694 | 0.010439 |
| IFNAR1 | 8394 | 3.180853 | 0.010462 |
| LCE3B | 9563 | 3.18038 | 0.010466 |
| TMEM90A | 18481 | 3.180345 | 0.010466 |
| SPATA18 | 17231 | 3.179344 | 0.010495 |
| NTSR1 | 12632 | 3.178868 | 0.010505 |
| FYCO1 | 6877 | 3.17795 | 0.010531 |
| SIM1 | 16521 | 3.177014 | 0.010553 |
| PPM1K | 14305 | 3.176941 | 0.010553 |
| NANOS2 | 12048 | 3.176411 | 0.010565 |
| C12orf42 | 2126 | 3.174365 | 0.010633 |
| MSGN1 | 11755 | 3.173365 | 0.01066 |
| ZBTB42 | 19902 | 3.173207 | 0.01066 |
| GJD2 | 7183 | 3.172814 | 0.01066 |
| APEH | 1154 | 3.17265 | 0.01066 |
| TRAF3 | 18709 | 3.172609 | 0.01066 |
| LOC100131082 | 9948 | 3.171563 | 0.010692 |
| KRT16P2 | 9333 | 3.169285 | 0.010764 |
| ID4 | 8343 | 3.169218 | 0.010764 |
| OR1F1 | 12883 | 3.167375 | 0.010825 |
| TLCD1 | 18188 | 3.166722 | 0.010838 |
| CDKL5 | 3721 | 3.166358 | 0.010838 |
| URB1 | 19333 | 3.166241 | 0.010838 |
| SNRNP27 | 17075 | 3.16618 | 0.010838 |
| KRTAP3-1 | 9435 | 3.166031 | 0.010838 |
| HSF1 | 8238 | 3.165757 | 0.010841 |
| TIMM8B | 18165 | 3.164712 | 0.010872 |
| G0S2 | 6891 | 3.164512 | 0.010872 |
| TCP1P3 | 17958 | 3.161877 | 0.010964 |
| KDM5B | 8998 | 3.161625 | 0.010966 |
| SLC9A7 | 16922 | 3.159411 | 0.011042 |
| MAFG | 10990 | 3.158782 | 0.011058 |
| GOLGA7B | 7333 | 3.156645 | 0.011125 |
| PPYR1 | 14384 | 3.156637 | 0.011125 |
| ZDHHC23 | 19975 | 3.156318 | 0.01113 |
| GRID2 | 7551 | 3.155542 | 0.011152 |
| LOC100288902 | 10145 | 3.154833 | 0.011172 |
| NEDD9 | 12219 | 3.154414 | 0.01118 |
| LOC100287723 | 10094 | 3.153235 | 0.011218 |
| KIAA1881 | 9136 | 3.152749 | 0.011229 |
| ARX | 1387 | 3.150651 | 0.011302 |
| PLG | 14037 | 3.149888 | 0.011324 |
| CTF1 | 4607 | 3.149192 | 0.011344 |
| ELMO2 | 5717 | 3.14747 | 0.011403 |
| DYDC2 | 5480 | 3.146582 | 0.011424 |
| C12orf52 | 2135 | 3.146349 | 0.011424 |
| TG | 18062 | 3.146336 | 0.011424 |
| MSLNL | 11768 | 3.145804 | 0.011437 |
| LZIC | 10964 | 3.145228 | 0.011452 |
| MPI | 11610 | 3.144963 | 0.011455 |
| ADAMTS19 | 573 | 3.144617 | 0.011461 |
| TLR9 | 18211 | 3.142936 | 0.011519 |
| YSK4 | 19847 | 3.142506 | 0.011528 |
| FBXL19 | 6410 | 3.142259 | 0.011531 |
| PGAM1P5 | 13718 | 3.140508 | 0.011592 |
| AC136604.1 | 390 | 3.140074 | 0.011594 |
| FAM135B | 6114 | 3.140054 | 0.011594 |
| GFRA4 | 7110 | 3.139632 | 0.011603 |
| TPST2 | 18694 | 3.138616 | 0.011636 |
| SNHG7 | 17063 | 3.137924 | 0.011652 |
| OR5C1 | 13077 | 3.137824 | 0.011652 |
| PCDHB7 | 13487 | 3.137595 | 0.011653 |
| FZD1 | 6880 | 3.137107 | 0.011664 |
| KIAA1239 | 9085 | 3.136929 | 0.011664 |
| S100A2 | 15964 | 3.135776 | 0.011703 |
| THAP4 | 18095 | 3.132741 | 0.011817 |
| ELOVL2 | 5725 | 3.131251 | 0.011869 |
| FAS | 6375 | 3.130842 | 0.01187 |
| NEFH | 12220 | 3.130836 | 0.01187 |
| NFATC1 | 12270 | 3.130429 | 0.011879 |
| LOC100130811 | 9941 | 3.130166 | 0.011881 |
| VAV2 | 19448 | 3.128443 | 0.011938 |
| LOC100128816 | 9885 | 3.128237 | 0.011938 |
| ODF3L1 | 12754 | 3.128198 | 0.011938 |
| PID1 | 13827 | 3.126912 | 0.011967 |
| CD300LB | 3572 | 3.126906 | 0.011967 |
| PCDH10 | 13448 | 3.126886 | 0.011967 |
| C9orf163 | 3029 | 3.124845 | 0.01204 |
| OR2A5 | 12909 | 3.124543 | 0.01204 |
| EMILIN2 | 5746 | 3.124494 | 0.01204 |
| TTBK2 | 18959 | 3.124329 | 0.01204 |
| SCO2 | 16125 | 3.123903 | 0.012044 |
| ACTRT2 | 525 | 3.123863 | 0.012044 |
| GLP2R | 7220 | 3.123584 | 0.012047 |
| ATP5EP2 | 1539 | 3.123102 | 0.012059 |
| PITPNC1 | 13898 | 3.121534 | 0.012116 |
| LOC644794 | 10498 | 3.119951 | 0.012172 |
| SYCE1 | 17670 | 3.119781 | 0.012172 |
| TRPS1 | 18877 | 3.119522 | 0.012175 |
| PP13 | 14243 | 3.119053 | 0.012186 |
| CDC45L | 3661 | 3.118591 | 0.012192 |
| CHST13 | 3965 | 3.118524 | 0.012192 |
| EPPK1 | 5845 | 3.116894 | 0.012246 |
| CEND1 | 3788 | 3.116848 | 0.012246 |
| KRT85 | 9380 | 3.116235 | 0.012263 |
| PLEKHO1 | 14035 | 3.115639 | 0.01228 |
| PKD2L1 | 13919 | 3.11451 | 0.012319 |
| HSPC081 | 8276 | 3.113971 | 0.012334 |
| WWOX | 19755 | 3.111736 | 0.01242 |
| PCDHB9 | 13489 | 3.110482 | 0.012464 |
| ZFAT | 19994 | 3.109554 | 0.012496 |
| IGF1 | 8418 | 3.108877 | 0.012516 |
| HIST1H3F | 7970 | 3.107674 | 0.012559 |
| CRTAC1 | 4473 | 3.106498 | 0.012601 |
| TSPAN15 | 18924 | 3.104194 | 0.012691 |
| GIP | 7158 | 3.103263 | 0.012719 |
| C19orf73 | 2419 | 3.10318 | 0.012719 |
| KCNA6 | 8868 | 3.101249 | 0.012786 |
| ISM2 | 8723 | 3.101203 | 0.012786 |
| LOC150519 | 10260 | 3.10105 | 0.012786 |
| SOX2 | 17172 | 3.100403 | 0.012805 |
| FAT1 | 6383 | 3.099325 | 0.012844 |
| HTT | 8307 | 3.098989 | 0.01285 |
| HTR6 | 8301 | 3.097745 | 0.012896 |
| MAB21L2 | 10972 | 3.097141 | 0.012914 |
| DCTN1 | 4915 | 3.096363 | 0.01294 |
| SLC20A2 | 16636 | 3.09533 | 0.012977 |
| GLT25D2 | 7234 | 3.094737 | 0.012994 |
| CCDC149 | 3349 | 3.094109 | 0.013013 |
| CD3D | 3583 | 3.091913 | 0.013102 |
| CCL24 | 3467 | 3.091471 | 0.013111 |
| RAMP2 | 14997 | 3.091325 | 0.013111 |
| MINA | 11452 | 3.089183 | 0.013197 |
| OR2W5 | 12959 | 3.088705 | 0.01321 |
| KLF4 | 9223 | 3.088313 | 0.013219 |
| UHMK1 | 19269 | 3.087497 | 0.013244 |
| C1orf85 | 2549 | 3.087383 | 0.013244 |
| HRG | 8194 | 3.084882 | 0.013332 |
| KCNJ14 | 8909 | 3.084808 | 0.013332 |
| TSHZ2 | 18907 | 3.084695 | 0.013332 |
| SPCS2 | 17253 | 3.084647 | 0.013332 |
| LOC100287521 | 10086 | 3.084399 | 0.013335 |
| OR14C36 | 12871 | 3.084128 | 0.013339 |
| CLP1 | 4121 | 3.083752 | 0.013347 |
| KCTD1 | 8961 | 3.083295 | 0.013359 |
| RELB | 15201 | 3.082326 | 0.013394 |
| AP005668.1 | 1111 | 3.080887 | 0.013451 |
| TMEM27 | 18412 | 3.079166 | 0.01352 |
| BCAR3 | 1741 | 3.078055 | 0.013562 |
| NEFL | 12221 | 3.077852 | 0.013563 |
| PPP1R9A | 14344 | 3.077449 | 0.013572 |
| KIF26B | 9170 | 3.077276 | 0.013572 |
| BPIL1 | 1891 | 3.077065 | 0.013573 |
| LOC644006 | 10485 | 3.076774 | 0.013578 |
| PSMB11 | 14641 | 3.075112 | 0.013645 |
| EXTL1 | 5985 | 3.073804 | 0.013697 |
| SERPINI2 | 16323 | 3.072008 | 0.013771 |
| PAPPA2 | 13367 | 3.070531 | 0.01383 |
| FAM164C | 6150 | 3.070328 | 0.013831 |
| WDR64 | 19653 | 3.069171 | 0.013876 |
| AEN | 661 | 3.068442 | 0.013896 |
| FLRT2 | 6678 | 3.068302 | 0.013896 |
| C9orf62 | 3050 | 3.068176 | 0.013896 |
| EFEMP1 | 5587 | 3.063586 | 0.014096 |
| OR2Z1 | 12961 | 3.063426 | 0.014096 |
| RUNDC1 | 15926 | 3.063352 | 0.014096 |
| ZNF226 | 20142 | 3.063016 | 0.014103 |
| SLC43A3 | 16836 | 3.061861 | 0.014148 |
| C4orf48 | 2807 | 3.061581 | 0.014153 |
| FTCD | 6829 | 3.060895 | 0.014176 |
| GNRH2 | 7319 | 3.060311 | 0.014192 |
| ACSBG1 | 478 | 3.060192 | 0.014192 |
| SLC39A5 | 16823 | 3.05953 | 0.014215 |
| PTGFRN | 14719 | 3.059063 | 0.014228 |
| S100A13 | 15961 | 3.057114 | 0.014312 |
| PROM2 | 14527 | 3.056003 | 0.01435 |
| SLC1A3 | 16630 | 3.055835 | 0.01435 |
| MLL2 | 11499 | 3.055552 | 0.01435 |
| AP000911.1 | 1092 | 3.055543 | 0.01435 |
| ZNF205 | 20128 | 3.055381 | 0.01435 |
| USP39 | 19384 | 3.055094 | 0.014355 |
| STK3 | 17538 | 3.054642 | 0.014361 |
| LOC100289550 | 10176 | 3.054467 | 0.014361 |
| CISD3 | 4004 | 3.054277 | 0.014361 |
| C7orf61 | 2958 | 3.05423 | 0.014361 |
| PDHB | 13606 | 3.053783 | 0.014374 |
| SLCO1C1 | 16930 | 3.053388 | 0.014384 |
| OR10J3 | 12835 | 3.052994 | 0.014394 |
| C17orf61 | 2327 | 3.052097 | 0.014428 |
| TJP3 | 18182 | 3.051141 | 0.014465 |
| DYSFIP1 | 5501 | 3.048804 | 0.014569 |
| MYL7 | 11951 | 3.047719 | 0.014613 |
| RCAN2 | 15152 | 3.046007 | 0.014687 |
| SPARCL1 | 17224 | 3.045557 | 0.0147 |
| SIK2 | 16517 | 3.044331 | 0.014751 |
| GSTM1 | 7619 | 3.042978 | 0.014809 |
| FAM215A | 6216 | 3.04212 | 0.014837 |
| SULF1 | 17615 | 3.042044 | 0.014837 |
| DKK1 | 5161 | 3.041312 | 0.014864 |
| GSTM2P1 | 7621 | 3.040927 | 0.014874 |
| C17orf74 | 2337 | 3.039786 | 0.01492 |
| GPC6 | 7378 | 3.039612 | 0.01492 |
| PLA2G5 | 13956 | 3.038857 | 0.014949 |
| PITX3 | 13905 | 3.03849 | 0.014958 |
| UGT2B17 | 19262 | 3.03782 | 0.014982 |
| BANK1 | 1700 | 3.036667 | 0.015022 |
| FMO5 | 6698 | 3.036643 | 0.015022 |
| NUDT8 | 12668 | 3.036128 | 0.015032 |
| LSM11 | 10879 | 3.036088 | 0.015032 |
| TUSC2 | 19082 | 3.035905 | 0.015032 |
| B3GNT6 | 1654 | 3.03569 | 0.015033 |
| LENG1 | 9616 | 3.03142 | 0.01523 |
| PHF23 | 13780 | 3.031397 | 0.01523 |
| WASF3 | 19579 | 3.029757 | 0.015304 |
| DMRT3 | 5207 | 3.029542 | 0.015306 |
| LOC642852 | 10465 | 3.029307 | 0.015308 |
| RHOBTB3 | 15318 | 3.029002 | 0.015314 |
| EGFEM1P | 5610 | 3.028198 | 0.015324 |
| SV2C | 17662 | 3.028066 | 0.015324 |
| TREX1 | 18749 | 3.027963 | 0.015324 |
| CTDP1 | 4602 | 3.027918 | 0.015324 |
| KRTAP4-2 | 9441 | 3.027906 | 0.015324 |
| HNF1A | 8085 | 3.027207 | 0.01535 |
| PRDX1 | 14433 | 3.025798 | 0.015412 |
| LRRC36 | 10809 | 3.024713 | 0.015458 |
| BMPER | 1862 | 3.02407 | 0.015467 |
| ZNF671 | 20451 | 3.024003 | 0.015467 |
| CORO2B | 4327 | 3.023826 | 0.015467 |
| SERINC3 | 16283 | 3.023815 | 0.015467 |
| TUBB | 19057 | 3.023463 | 0.015476 |
| MIR22HG | 11467 | 3.022813 | 0.0155 |
| COL4A4 | 4265 | 3.022459 | 0.015509 |
| GJB2 | 7174 | 3.020968 | 0.015576 |
| ABCC12 | 61 | 3.020477 | 0.015592 |
| MT1E | 11786 | 3.019236 | 0.015647 |
| FLT3LG | 6682 | 3.018244 | 0.015684 |
| C13orf36 | 2170 | 3.018143 | 0.015684 |
| STARD13 | 17498 | 3.01737 | 0.015713 |
| MOXD1 | 11601 | 3.017223 | 0.015713 |
| AC011498.2 | 200 | 3.01692 | 0.015718 |
| PCDH20 | 13456 | 3.016691 | 0.015718 |
| SRGAP1 | 17372 | 3.016599 | 0.015718 |
| SPTLC1 | 17349 | 3.016125 | 0.015733 |
| PLCB4 | 13980 | 3.01583 | 0.015739 |
| LOC442249 | 10442 | 3.01412 | 0.015818 |
| FLRT1 | 6677 | 3.013572 | 0.01583 |
| ZNF593 | 20394 | 3.013541 | 0.01583 |
| LOC100287593 | 10091 | 3.012557 | 0.015872 |
| RP11-269F19.1 | 15563 | 3.012011 | 0.015891 |
| TUBA1B | 19047 | 3.011699 | 0.015898 |
| HTR3A | 8294 | 3.011355 | 0.0159 |
| C16orf55 | 2276 | 3.011292 | 0.0159 |
| PTPRD | 14769 | 3.010972 | 0.015908 |
| SATB2 | 16041 | 3.010603 | 0.015917 |
| ZKSCAN1 | 20050 | 3.00868 | 0.016002 |
| AC100756.1 | 330 | 3.008636 | 0.016002 |
| FAM173B | 6165 | 3.00832 | 0.016009 |
| AC134982.1 | 380 | 3.008088 | 0.016012 |
| UBE2G2 | 19165 | 3.007466 | 0.016035 |
| BEGAIN | 1791 | 3.006938 | 0.016044 |
| SLC20A1 | 16635 | 3.006807 | 0.016044 |
| MCF2L-AS1 | 11222 | 3.006762 | 0.016044 |
| GCLM | 7054 | 3.006572 | 0.016045 |
| GRB7 | 7539 | 3.006176 | 0.016056 |
| UBC | 19150 | 3.005896 | 0.016061 |
| PRSS21 | 14586 | 3.005268 | 0.016085 |
| OR2L13 | 12933 | 3.002951 | 0.016199 |
| ARL6IP1 | 1322 | 3.00242 | 0.01621 |
| CD70 | 3606 | 3.002384 | 0.01621 |
| AC022098.3 | 238 | 3.001587 | 0.016243 |
| NT5DC2 | 12613 | 3.001109 | 0.016259 |
| SLC38A8 | 16813 | 3.000223 | 0.01629 |
| DUOXA1 | 5435 | 3.000176 | 0.01629 |
| PPP1R28 | 14332 | 2.998867 | 0.01635 |
| INHBE | 8602 | 2.997247 | 0.016428 |
| CABP2 | 3096 | 2.996968 | 0.016433 |
| TBX10 | 17892 | 2.995277 | 0.016514 |
| NDUFB1 | 12181 | 2.995124 | 0.016514 |
| TMX4 | 18530 | 2.994652 | 0.016529 |
| MGA | 11387 | 2.993183 | 0.0166 |
| ASXL2 | 1453 | 2.992555 | 0.016609 |
| ITPKA | 8781 | 2.992487 | 0.016609 |
| WWC1 | 19752 | 2.992485 | 0.016609 |
| ADORA2B | 638 | 2.992045 | 0.016623 |
| CYP2J2 | 4783 | 2.991606 | 0.016637 |
| ZSCAN20 | 20621 | 2.991427 | 0.016637 |
| WIF1 | 19706 | 2.990889 | 0.016657 |
| C7orf43 | 2943 | 2.989485 | 0.016721 |
| ABCC6 | 67 | 2.989371 | 0.016721 |
| VANGL2 | 19436 | 2.986321 | 0.016878 |
| BRUNOL6 | 1932 | 2.985176 | 0.016932 |
| SHBG | 16469 | 2.984513 | 0.016959 |
| ARHGEF4 | 1285 | 2.983496 | 0.017005 |
| CALCR | 3143 | 2.9819 | 0.017085 |
| LOC393078 | 10371 | 2.981479 | 0.017098 |
| FGL1 | 6569 | 2.980886 | 0.017121 |
| NDOR1 | 12152 | 2.978359 | 0.017253 |
| PKN3 | 13932 | 2.977316 | 0.017302 |
| SELRC1 | 16217 | 2.976875 | 0.017317 |
| IGSF21 | 8457 | 2.976141 | 0.017348 |
| CDC14A | 3630 | 2.974391 | 0.017438 |
| CRKRS | 4458 | 2.973732 | 0.017463 |
| ZDHHC21 | 19973 | 2.973446 | 0.017463 |
| FAM136A | 6115 | 2.973416 | 0.017463 |
| CROCC | 4467 | 2.972456 | 0.017508 |
| STX11 | 17580 | 2.972057 | 0.01752 |
| EHF | 5628 | 2.971153 | 0.017562 |
| TST | 18956 | 2.969934 | 0.017622 |
| PITPNM3 | 13901 | 2.968975 | 0.017667 |
| RANBP3L | 15005 | 2.96735 | 0.01775 |
| NRSN1 | 12579 | 2.96696 | 0.017763 |
| DNAJC1 | 5250 | 2.96581 | 0.017819 |
| RHOF | 15321 | 2.964478 | 0.017886 |
| KLC3 | 9209 | 2.963626 | 0.017925 |
| ATP2C2 | 1531 | 2.963351 | 0.017931 |
| ITPR2 | 8785 | 2.963008 | 0.017941 |
| MCHR2 | 11226 | 2.962653 | 0.017951 |
| TRIM72 | 18824 | 2.96053 | 0.018065 |
| MT2A | 11793 | 2.959361 | 0.018124 |
| KRTAP19-7 | 9418 | 2.958495 | 0.018164 |
| SSR2 | 17420 | 2.956786 | 0.018255 |
| ALDH4A1 | 831 | 2.955914 | 0.018296 |
| AC021054.1 | 231 | 2.955294 | 0.018323 |
| ACTL8 | 508 | 2.955033 | 0.018328 |
| CTDSP1 | 4603 | 2.954179 | 0.018368 |
| FGF3 | 6550 | 2.953843 | 0.018378 |
| PIGV | 13846 | 2.953237 | 0.018378 |
| GTF2H3 | 7644 | 2.953187 | 0.018378 |
| SEMA4G | 16231 | 2.953139 | 0.018378 |
| FAM155B | 6136 | 2.953134 | 0.018378 |
| KCNK7 | 8933 | 2.952488 | 0.018406 |
| PLA2G2C | 13946 | 2.951791 | 0.018437 |
| EFNB1 | 5601 | 2.949065 | 0.01859 |
| SIRPG | 16534 | 2.948252 | 0.018629 |
| PTPN18 | 14753 | 2.946609 | 0.018717 |
| SLC6A7 | 16892 | 2.946415 | 0.018719 |
| EVI1 | 5945 | 2.945925 | 0.018738 |
| ZNRF3 | 20602 | 2.945591 | 0.018739 |
| CCR7 | 3516 | 2.945554 | 0.018739 |
| WDR41 | 19628 | 2.945209 | 0.018749 |
| MBOAT1 | 11198 | 2.9438 | 0.018824 |
| CARD17 | 3215 | 2.942947 | 0.018866 |
| MKX | 11488 | 2.942641 | 0.018874 |
| FLT3 | 6681 | 2.939553 | 0.01904 |
| HLF | 8037 | 2.939408 | 0.01904 |
| ACTG1 | 501 | 2.939354 | 0.01904 |
| DNAJC21 | 5262 | 2.939234 | 0.01904 |
| SLC35E4 | 16789 | 2.938074 | 0.0191 |
| MOBKL2B | 11564 | 2.937878 | 0.019102 |
| MSANTD1 | 11752 | 2.935654 | 0.019228 |
| CDCA3 | 3667 | 2.935313 | 0.019234 |
| COQ4 | 4317 | 2.935055 | 0.019234 |
| FAM78A | 6315 | 2.935043 | 0.019234 |
| LINGO1 | 9802 | 2.9344 | 0.019263 |
| SLC5A2 | 16867 | 2.933545 | 0.019306 |
| NPAS2 | 12478 | 2.932229 | 0.019377 |
| ZNF474 | 20299 | 2.930885 | 0.01945 |
| SYDE2 | 17678 | 2.930154 | 0.019485 |
| CDGAP | 3674 | 2.929863 | 0.019492 |
| GATC | 7019 | 2.929327 | 0.019515 |
| GPR27 | 7463 | 2.92827 | 0.019568 |
| RAB13 | 14874 | 2.928139 | 0.019568 |
| MRPL41 | 11687 | 2.927661 | 0.019581 |
| WWTR1 | 19758 | 2.92759 | 0.019581 |
| CARS2 | 3224 | 2.927372 | 0.019584 |
| IPMK | 8651 | 2.925362 | 0.0197 |
| ALKBH8 | 863 | 2.924546 | 0.019734 |
| SLC18A1 | 16622 | 2.924469 | 0.019734 |
| TNFRSF11B | 18546 | 2.921955 | 0.019883 |
| SYNM | 17696 | 2.921686 | 0.01989 |
| GNL3L | 7309 | 2.920392 | 0.01996 |
| EFCAB4A | 5581 | 2.920247 | 0.01996 |
| ABCC9 | 69 | 2.919723 | 0.019982 |
| CHCHD8 | 3888 | 2.919234 | 0.020002 |
| Z83840.4 | 19863 | 2.918363 | 0.020047 |
| RP4-697K14.1 | 15670 | 2.917675 | 0.020081 |
| VPS35 | 19524 | 2.91569 | 0.020189 |
| TNFSF9 | 18572 | 2.915651 | 0.020189 |
| LOC283177 | 10285 | 2.914944 | 0.020217 |
| SLC39A10 | 16816 | 2.914873 | 0.020217 |
| MUC3 | 11869 | 2.912387 | 0.020367 |
| LRG1 | 10761 | 2.912153 | 0.020372 |
| KRTAP2-1 | 9420 | 2.911753 | 0.020386 |
| ABHD10 | 84 | 2.911166 | 0.020414 |
| HSD17B7P2 | 8230 | 2.910532 | 0.020444 |
| LEPREL1 | 9625 | 2.909574 | 0.020495 |
| CTSF | 4634 | 2.909253 | 0.020505 |
| BTBD9 | 1959 | 2.908942 | 0.020514 |
| FAM184A | 6184 | 2.907742 | 0.02058 |
| TIGD4 | 18149 | 2.907598 | 0.02058 |
| UBE2D4 | 19160 | 2.906686 | 0.020629 |
| HELB | 7849 | 2.906262 | 0.020646 |
| RASL11B | 15057 | 2.90595 | 0.020655 |
| FBRSL1 | 6402 | 2.904937 | 0.02071 |
| MARCH8 | 11146 | 2.90477 | 0.02071 |
| BRI3BP | 1916 | 2.904313 | 0.020729 |
| CCDC8 | 3423 | 2.903562 | 0.020768 |
| RALA | 14988 | 2.902116 | 0.020834 |
| OR4A15 | 12965 | 2.902081 | 0.020834 |
| BARX1 | 1706 | 2.902052 | 0.020834 |
| C11orf83 | 2095 | 2.901216 | 0.020864 |
| CD164L2 | 3542 | 2.901094 | 0.020864 |
| UCA1 | 19229 | 2.901094 | 0.020864 |
| RAB4A | 14922 | 2.899073 | 0.020987 |
| DENND1A | 5034 | 2.898459 | 0.021006 |
| RP11-3B7.1 | 15592 | 2.898455 | 0.021006 |
| RAX | 15071 | 2.897253 | 0.021075 |
| SPAG9 | 17211 | 2.897029 | 0.021079 |
| RAMP3 | 14998 | 2.896282 | 0.021118 |
| DCTN3 | 4917 | 2.895762 | 0.021141 |
| C1orf106 | 2452 | 2.893087 | 0.021303 |
| ZBTB12 | 19882 | 2.89293 | 0.021303 |
| PEX14 | 13685 | 2.892851 | 0.021303 |
| CMAH | 4145 | 2.892456 | 0.021319 |
| KIF3A | 9175 | 2.89085 | 0.021416 |
| C11orf48 | 2072 | 2.890331 | 0.02144 |
| BZRPL1 | 1994 | 2.890004 | 0.021451 |
| EVX1 | 5953 | 2.889437 | 0.021478 |
| WDR48 | 19637 | 2.889079 | 0.021491 |
| SLC8A3 | 16911 | 2.888292 | 0.021533 |
| ENSA | 5788 | 2.887981 | 0.021543 |
| KRTAP4-1 | 9438 | 2.887575 | 0.021559 |
| RPL38 | 15763 | 2.88711 | 0.021571 |
| LINGO4 | 9805 | 2.886721 | 0.021571 |
| PRDM1 | 14418 | 2.886718 | 0.021571 |
| SGK493 | 16409 | 2.886591 | 0.021571 |
| GRM8 | 7583 | 2.886553 | 0.021571 |
| LRRC14 | 10786 | 2.884365 | 0.02171 |
| GGCT | 7115 | 2.882565 | 0.021823 |
| IL17A | 8490 | 2.881378 | 0.021893 |
| BCL11B | 1757 | 2.880521 | 0.021941 |
| CSDC2 | 4508 | 2.880031 | 0.021964 |
| FIGLA | 6588 | 2.878317 | 0.022071 |
| HIST1H4K | 7985 | 2.878095 | 0.022075 |
| STEAP2 | 17521 | 2.877119 | 0.022132 |
| ZNF354C | 20230 | 2.876731 | 0.02214 |
| WARS2 | 19575 | 2.876631 | 0.02214 |
| PRR11 | 14555 | 2.876496 | 0.02214 |
| SNTB2 | 17095 | 2.87553 | 0.022196 |
| KIF21B | 9164 | 2.874878 | 0.02223 |
| RASSF3 | 15062 | 2.873885 | 0.022281 |
| GRIK4 | 7556 | 2.873824 | 0.022281 |
| CHRM2 | 3941 | 2.872836 | 0.022339 |
| CDH9 | 3698 | 2.871465 | 0.022424 |
| TMEM191B | 18370 | 2.871162 | 0.022433 |
| LGALS8 | 9645 | 2.870727 | 0.022452 |
| TFCP2 | 18044 | 2.870426 | 0.022452 |
| POLR3A | 14181 | 2.870397 | 0.022452 |
| C7orf41 | 2941 | 2.869344 | 0.022515 |
| C1orf2 | 2506 | 2.868809 | 0.022541 |
| ELMO1 | 5716 | 2.867738 | 0.022606 |
| ICAM4 | 8333 | 2.867527 | 0.022609 |
| C6orf192 | 2890 | 2.867145 | 0.022624 |
| MCART6 | 11213 | 2.865223 | 0.02275 |
| RHOJ | 15324 | 2.864781 | 0.022769 |
| EGFL7 | 5612 | 2.864183 | 0.0228 |
| GHRLOS2 | 7141 | 2.863978 | 0.022803 |
| LINC00174 | 9731 | 2.863642 | 0.022815 |
| RNF38 | 15475 | 2.863116 | 0.022836 |
| PGM1 | 13743 | 2.863017 | 0.022836 |
| ADAM19 | 547 | 2.862239 | 0.022874 |
| PON2 | 14208 | 2.862162 | 0.022874 |
| HS3ST5 | 8209 | 2.861961 | 0.022876 |
| HBZ | 7781 | 2.861591 | 0.022891 |
| C14orf68 | 2225 | 2.860822 | 0.022934 |
| KCNJ10 | 8905 | 2.859871 | 0.022991 |
| LOC643997 | 10484 | 2.858701 | 0.023064 |
| PLS1 | 14055 | 2.857954 | 0.023106 |
| DCUN1D3 | 4924 | 2.85759 | 0.023121 |
| KLF17 | 9219 | 2.856748 | 0.02317 |
| MMP17 | 11533 | 2.855666 | 0.023237 |
| HPS6 | 8181 | 2.85512 | 0.023265 |
| COL24A1 | 4254 | 2.854826 | 0.023274 |
| RBP2 | 15138 | 2.854162 | 0.023311 |
| APOL4 | 1193 | 2.853652 | 0.023336 |
| SLC25A15 | 16676 | 2.853039 | 0.023369 |
| SERPINF1 | 16318 | 2.851926 | 0.023438 |
| SNN | 17066 | 2.849522 | 0.023604 |
| HEPH | 7858 | 2.848604 | 0.02366 |
| LOC727916 | 10595 | 2.848264 | 0.023673 |
| TMEM158 | 18328 | 2.847009 | 0.023754 |
| CYB561D1 | 4729 | 2.846753 | 0.023758 |
| FAM73A | 6301 | 2.84658 | 0.023758 |
| FZD7 | 6887 | 2.846451 | 0.023758 |
| PINK1 | 13880 | 2.84608 | 0.023774 |
| ARL4D | 1317 | 2.845732 | 0.023787 |
| HSPH1 | 8280 | 2.844845 | 0.023836 |
| ZIC3 | 20044 | 2.844743 | 0.023836 |
| LRP2 | 10774 | 2.843609 | 0.023909 |
| UBXN6 | 19226 | 2.843024 | 0.023941 |
| UGT1A9 | 19257 | 2.842547 | 0.023952 |
| CCDC109B | 3312 | 2.842483 | 0.023952 |
| S100A1 | 15957 | 2.842374 | 0.023952 |
| RTN4RL2 | 15916 | 2.842195 | 0.023953 |
| DHH | 5092 | 2.841691 | 0.023979 |
| PLCB1 | 13977 | 2.840725 | 0.024039 |
| LOC100131294 | 9957 | 2.83931 | 0.024133 |
| ECHS1 | 5539 | 2.837276 | 0.024275 |
| MED12L | 11272 | 2.836883 | 0.024292 |
| SFTA1P | 16387 | 2.835651 | 0.024365 |
| HSD17B12 | 8222 | 2.835603 | 0.024365 |
| C11ORF36 | 2054 | 2.835417 | 0.024366 |
| SNX24 | 17116 | 2.833822 | 0.024476 |
| PSMB7 | 14647 | 2.83351 | 0.024477 |
| MMD2 | 11521 | 2.833481 | 0.024477 |
| CPEB3 | 4371 | 2.832282 | 0.024556 |
| MRPS21 | 11716 | 2.830968 | 0.024644 |
| HBQ1 | 7778 | 2.830735 | 0.02465 |
| MFN2 | 11372 | 2.82864 | 0.024799 |
| DIRAS1 | 5141 | 2.828326 | 0.02481 |
| NKX6-3 | 12374 | 2.827933 | 0.024827 |
| SLC4A5 | 16859 | 2.827783 | 0.024827 |
| ZIC2 | 20043 | 2.827566 | 0.024829 |
| P2RY4 | 13285 | 2.827427 | 0.024829 |
| BIRC3 | 1831 | 2.82654 | 0.024884 |
| SDC4 | 16149 | 2.826386 | 0.024884 |
| TYW1 | 19125 | 2.823476 | 0.025099 |
| KIF18B | 9156 | 2.820926 | 0.025286 |
| PSPN | 14685 | 2.820113 | 0.025337 |
| TRPV5 | 18883 | 2.819768 | 0.025352 |
| ZNF737 | 20503 | 2.819563 | 0.025355 |
| ETHE1 | 5930 | 2.818533 | 0.025423 |
| TLN2 | 18200 | 2.817942 | 0.025457 |
| TNIP3 | 18576 | 2.817165 | 0.025506 |
| MRGPRD | 11644 | 2.816802 | 0.025522 |
| MORC2 | 11582 | 2.816221 | 0.025555 |
| TMEM184A | 18358 | 2.815817 | 0.025574 |
| IGLON5 | 8451 | 2.81535 | 0.025598 |
| ASCL2 | 1418 | 2.814442 | 0.025658 |
| GPR37L1 | 7470 | 2.813396 | 0.025728 |
| VIP | 19486 | 2.81192 | 0.025833 |
| NOS1 | 12455 | 2.811234 | 0.025875 |
| ADD1 | 614 | 2.809933 | 0.025967 |
| TBX21 | 17898 | 2.808635 | 0.026058 |
| HHAT | 7895 | 2.808482 | 0.026058 |
| B3GNT3 | 1651 | 2.80767 | 0.02611 |
| SEPT9 | 16270 | 2.807187 | 0.026136 |
| RCN2 | 15163 | 2.806625 | 0.026169 |
| PPIF | 14287 | 2.806294 | 0.026172 |
| ZAN | 19868 | 2.806255 | 0.026172 |
| OR2T5 | 12953 | 2.805107 | 0.026252 |
| PDGFRA | 13601 | 2.804171 | 0.026296 |
| PRY | 14607 | 2.804109 | 0.026296 |
| RNASE13 | 15386 | 2.804086 | 0.026296 |
| TTN | 19018 | 2.80217 | 0.026439 |
| LHX6 | 9674 | 2.798723 | 0.026709 |
| LOC650095 | 10556 | 2.797547 | 0.026793 |
| DPH3P1 | 5346 | 2.796696 | 0.02685 |
| SRCRB4D | 17363 | 2.795952 | 0.026883 |
| OXSR1 | 13266 | 2.795929 | 0.026883 |
| RP11-257K9.7 | 15560 | 2.795812 | 0.026883 |
| CELA3A | 3781 | 2.795499 | 0.026896 |
| CHD1L | 3890 | 2.794621 | 0.026955 |
| LCE5A | 9568 | 2.793576 | 0.027029 |
| RNF43 | 15479 | 2.792623 | 0.027095 |
| DRD4 | 5387 | 2.790475 | 0.027262 |
| GPR148 | 7429 | 2.789517 | 0.027329 |
| PRKX | 14495 | 2.788423 | 0.027408 |
| CTNNBIP1 | 4617 | 2.787877 | 0.02744 |
| GFOD2 | 7104 | 2.786308 | 0.02755 |
| SLC14A2 | 16594 | 2.786265 | 0.02755 |
| HBM | 7776 | 2.782409 | 0.027865 |
| QRICH2 | 14854 | 2.782001 | 0.027886 |
| FOXR1 | 6771 | 2.781825 | 0.027887 |
| ITSN1 | 8790 | 2.780599 | 0.027969 |
| SCARF2 | 16075 | 2.780503 | 0.027969 |
| P2RY8 | 13287 | 2.780285 | 0.027969 |
| SLAMF8 | 16567 | 2.780226 | 0.027969 |
| HNF1B | 8087 | 2.779681 | 0.028002 |
| FAM107A | 6051 | 2.777979 | 0.028135 |
| KRTAP5-7 | 9457 | 2.776618 | 0.028239 |
| LZTR1 | 10966 | 2.775682 | 0.028307 |
| MED7 | 11295 | 2.775321 | 0.028324 |
| RAP2A | 15014 | 2.774368 | 0.028393 |
| SNAP29 | 17040 | 2.773578 | 0.028448 |
| KCNQ5 | 8950 | 2.772654 | 0.028514 |
| TFCP2L1 | 18045 | 2.772501 | 0.028514 |
| ZFHX4 | 19997 | 2.770779 | 0.028651 |
| CTRB1 | 4625 | 2.770354 | 0.028674 |
| LOC100288578 | 10131 | 2.768785 | 0.028798 |
| ARPP19 | 1360 | 2.768504 | 0.028809 |
| MYC | 11914 | 2.76777 | 0.028859 |
| CDK2AP2 | 3703 | 2.767487 | 0.02887 |
| EVPL | 5951 | 2.766946 | 0.028904 |
| LCE1A | 9552 | 2.766235 | 0.02894 |
| SSU72P1 | 17434 | 2.766219 | 0.02894 |
| NOL6 | 12440 | 2.765714 | 0.02897 |
| AGSK1 | 711 | 2.764428 | 0.029069 |
| PPFIBP1 | 14275 | 2.76414 | 0.029069 |
| PCP4L1 | 13536 | 2.764124 | 0.029069 |
| HIST1H4F | 7980 | 2.762907 | 0.029163 |
| LGR5 | 9655 | 2.762543 | 0.029181 |
| ST3GAL3 | 17456 | 2.761804 | 0.029233 |
| RILP | 15344 | 2.760864 | 0.029296 |
| PLAC4 | 13964 | 2.760779 | 0.029296 |
| CPNE5 | 4384 | 2.759959 | 0.029355 |
| RP4-665J23.1 | 15668 | 2.759737 | 0.02936 |
| LOC727796 | 10589 | 2.758405 | 0.029466 |
| ROBO2 | 15501 | 2.757892 | 0.029498 |
| ANGEL1 | 937 | 2.757309 | 0.029536 |
| HIC1 | 7908 | 2.754616 | 0.029765 |
| LRRC20 | 10795 | 2.753446 | 0.029843 |
| CACNA1H | 3109 | 2.75338 | 0.029843 |
| KLK4 | 9285 | 2.753288 | 0.029843 |
| NFAM1 | 12267 | 2.752749 | 0.029877 |
| TMEM26 | 18411 | 2.75217 | 0.029915 |
| ADAMTSL2 | 584 | 2.75117 | 0.029964 |
| TNFRSF11A | 18545 | 2.75109 | 0.029964 |
| S1PR1 | 15979 | 2.750896 | 0.029964 |
| ZSCAN10 | 20616 | 2.750875 | 0.029964 |
| OGG1 | 12769 | 2.750758 | 0.029964 |
| NCBP1 | 12113 | 2.750673 | 0.029964 |
| WDR36 | 19622 | 2.749491 | 0.030058 |
| FPR2 | 6780 | 2.748717 | 0.030114 |
| SEC24D | 16196 | 2.748367 | 0.030132 |
| CBX8 | 3291 | 2.747789 | 0.03017 |
| SYN1 | 17682 | 2.746686 | 0.030239 |
| ATP13A5 | 1514 | 2.746615 | 0.030239 |
| GPR97 | 7498 | 2.746565 | 0.030239 |
| LRRC37A16P | 10810 | 2.746327 | 0.030246 |
| ADPRHL1 | 642 | 2.745571 | 0.030301 |
| RP11-267N12.3 | 15562 | 2.744988 | 0.03034 |
| ETV1 | 5935 | 2.744632 | 0.030358 |
| MRAS | 11637 | 2.742991 | 0.030496 |
| C1orf158 | 2484 | 2.742715 | 0.0305 |
| MYOM2 | 11992 | 2.742628 | 0.0305 |
| LOC100287988 | 10107 | 2.741098 | 0.030628 |
| SST | 17426 | 2.740203 | 0.030696 |
| IDI1 | 8350 | 2.73984 | 0.030709 |
| ZFP36L1 | 20008 | 2.739745 | 0.030709 |
| AGBL4 | 688 | 2.738907 | 0.030764 |
| GFRAL | 7111 | 2.738845 | 0.030764 |
| CDC6 | 3663 | 2.738416 | 0.030775 |
| SPRED1 | 17317 | 2.738401 | 0.030775 |
| C2orf71 | 2713 | 2.73807 | 0.030792 |
| BTRC | 1983 | 2.7377 | 0.030811 |
| NT5C1A | 12607 | 2.737542 | 0.030811 |
| GNPDA1 | 7313 | 2.737184 | 0.030822 |
| TMEM120A | 18285 | 2.737109 | 0.030822 |
| TBC1D23 | 17853 | 2.735564 | 0.030952 |
| ABCG8 | 82 | 2.735195 | 0.030972 |
| CSAG1 | 4504 | 2.73501 | 0.030975 |
| KCNC2 | 8876 | 2.733419 | 0.03111 |
| ZDHHC5 | 19979 | 2.733242 | 0.031111 |
| C17orf103 | 2311 | 2.732256 | 0.03119 |
| C3orf59 | 2765 | 2.731309 | 0.031265 |
| CAPS | 3204 | 2.72999 | 0.031375 |
| GOLPH3L | 7342 | 2.728987 | 0.031443 |
| GCDH | 7045 | 2.728956 | 0.031443 |
| IFIH1 | 8373 | 2.728213 | 0.031499 |
| TAAR1 | 17729 | 2.728022 | 0.031502 |
| GPR35 | 7468 | 2.727134 | 0.031572 |
| HRK | 8199 | 2.726733 | 0.031586 |
| CDH6 | 3695 | 2.726474 | 0.031586 |
| MT1M | 11791 | 2.726443 | 0.031586 |
| STAC2 | 17482 | 2.726359 | 0.031586 |
| SEPT3 | 16263 | 2.724371 | 0.031761 |
| AOC2 | 1077 | 2.724166 | 0.031766 |
| NPY1R | 12524 | 2.721562 | 0.032002 |
| ZNF175 | 20108 | 2.720707 | 0.032062 |
| OR4K5 | 12997 | 2.720569 | 0.032062 |
| SNAI3 | 17037 | 2.720467 | 0.032062 |
| CDC25A | 3638 | 2.71986 | 0.032106 |
| C21orf128 | 2625 | 2.719366 | 0.032139 |
| C13orf15 | 2158 | 2.719053 | 0.032154 |
| AC017096.1 | 224 | 2.718813 | 0.032162 |
| ABI3 | 100 | 2.718127 | 0.032213 |
| TDRKH | 17982 | 2.717669 | 0.032242 |
| DHCR24 | 5086 | 2.716503 | 0.032341 |
| EXT2 | 5984 | 2.715864 | 0.032388 |
| OGDH | 12763 | 2.715363 | 0.032417 |
| HTRA1 | 8303 | 2.71525 | 0.032417 |
| CBWD6 | 3282 | 2.714797 | 0.032446 |
| TRIM16 | 18765 | 2.712823 | 0.032619 |
| ANGPTL6 | 947 | 2.71272 | 0.032619 |
| MGAT4C | 11393 | 2.712445 | 0.032631 |
| CNN1 | 4180 | 2.711337 | 0.032725 |
| UBE3B | 19189 | 2.710847 | 0.032758 |
| NOX5 | 12474 | 2.709716 | 0.032854 |
| TP53TG1 | 18654 | 2.708631 | 0.032946 |
| AC009139.1 | 174 | 2.707601 | 0.033033 |
| BRK1 | 1919 | 2.707357 | 0.033042 |
| TMBIM6 | 18236 | 2.70708 | 0.033054 |
| PDZD2 | 13643 | 2.705787 | 0.033167 |
| AC073135.1 | 281 | 2.704517 | 0.033274 |
| RUNDC2A | 15927 | 2.704402 | 0.033274 |
| NUMB | 12675 | 2.703664 | 0.033332 |
| DPY19L1 | 5365 | 2.701356 | 0.033549 |
| TMEM93 | 18484 | 2.700616 | 0.033608 |
| LINC00339 | 9771 | 2.699821 | 0.033672 |
| PYCRL | 14831 | 2.69859 | 0.033781 |
| CEP250 | 3814 | 2.697939 | 0.033832 |
| CPZ | 4410 | 2.697749 | 0.033835 |
| THTPA | 18130 | 2.697309 | 0.033864 |
| AC079341.1 | 291 | 2.696627 | 0.033917 |
| CDCA7 | 3670 | 2.695695 | 0.033996 |
| TXNDC5 | 19105 | 2.694517 | 0.034101 |
| EXOC3L2 | 5964 | 2.693943 | 0.034144 |
| CLDN6 | 4059 | 2.692909 | 0.034234 |
| DENND2A | 5037 | 2.692449 | 0.034263 |
| KRTAP4-5 | 9444 | 2.692314 | 0.034263 |
| PTH2R | 14729 | 2.691313 | 0.03435 |
| ATP8B3 | 1584 | 2.690642 | 0.034403 |
| ARHGEF16 | 1279 | 2.688527 | 0.034605 |
| LCE2D | 9561 | 2.68791 | 0.034653 |
| NBAS | 12088 | 2.687147 | 0.034716 |
| ANKRD53 | 1020 | 2.685968 | 0.034823 |
| GPR119 | 7409 | 2.685238 | 0.034882 |
| TESK1 | 18013 | 2.68499 | 0.034892 |
| ZSWIM4 | 20632 | 2.68424 | 0.034954 |
| XPO6 | 19795 | 2.683487 | 0.035017 |
| SSTR5 | 17431 | 2.682893 | 0.035063 |
| GRIK3 | 7555 | 2.68093 | 0.035248 |
| KCNB1 | 8873 | 2.680819 | 0.035248 |
| ACADM | 419 | 2.679814 | 0.035337 |
| TPRG1L | 18686 | 2.678107 | 0.035499 |
| ZFAND3 | 19991 | 2.677971 | 0.035499 |
| C18orf1 | 2354 | 2.677776 | 0.035504 |
| CPAMD8 | 4364 | 2.677524 | 0.035514 |
| SPACA1 | 17197 | 2.677198 | 0.035526 |
| NTN4 | 12623 | 2.677102 | 0.035526 |
| LARGE | 9519 | 2.676757 | 0.03553 |
| EPHB6 | 5833 | 2.676755 | 0.03553 |
| LOC100290528 | 10198 | 2.676238 | 0.035568 |
| CD8A | 3617 | 2.675069 | 0.035676 |
| MED16 | 11277 | 2.674506 | 0.035719 |
| C1orf50 | 2527 | 2.674168 | 0.035739 |
| MAGIX | 11027 | 2.67396 | 0.035744 |
| RBPJL | 15143 | 2.672744 | 0.035858 |
| ZC4H2 | 19939 | 2.671949 | 0.035926 |
| TIPARP | 18175 | 2.670658 | 0.036048 |
| GANAB | 6987 | 2.669568 | 0.036148 |
| CBX1 | 3283 | 2.668222 | 0.036277 |
| EIF4E | 5681 | 2.667522 | 0.036336 |
| ZNF844 | 20577 | 2.666851 | 0.036392 |
| NKX2-6 | 12368 | 2.666492 | 0.036411 |
| RNF207 | 15457 | 2.666362 | 0.036411 |
| SIDT1 | 16501 | 2.666166 | 0.036415 |
| TUBA3D | 19050 | 2.664994 | 0.036526 |
| KLHL29 | 9259 | 2.66455 | 0.036557 |
| BARHL2 | 1705 | 2.662465 | 0.036768 |
| TTR | 19021 | 2.66173 | 0.036824 |
| BAI1 | 1689 | 2.661645 | 0.036824 |
| STK40 | 17550 | 2.660597 | 0.036921 |
| CCDC120 | 3323 | 2.659802 | 0.036992 |
| TMSB10 | 18515 | 2.659067 | 0.037056 |
| DBX1 | 4877 | 2.658642 | 0.037072 |
| NEUROG1 | 12260 | 2.658611 | 0.037072 |
| ACTN1 | 510 | 2.658326 | 0.037074 |
| HMGB1P16 | 8051 | 2.658216 | 0.037074 |
| SUSD5 | 17654 | 2.658131 | 0.037074 |
| GAS6 | 7006 | 2.657643 | 0.037089 |
| ABCC11 | 60 | 2.657618 | 0.037089 |
| C16orf35 | 2268 | 2.657526 | 0.037089 |
| ESAM | 5905 | 2.656929 | 0.037138 |
| FUT10 | 6854 | 2.656657 | 0.037151 |
| C3orf62 | 2766 | 2.656401 | 0.037163 |
| AC068580.3 | 273 | 2.655175 | 0.037281 |
| LOC400352 | 10379 | 2.654671 | 0.03732 |
| LYPD1 | 10938 | 2.654165 | 0.037359 |
| LOC100287525 | 10087 | 2.652563 | 0.037519 |
| NOX4 | 12473 | 2.651939 | 0.037572 |
| SH2D6 | 16435 | 2.65067 | 0.037696 |
| RAET1K | 14979 | 2.650206 | 0.037731 |
| PRMT6 | 14509 | 2.64982 | 0.037753 |
| CLDND2 | 4064 | 2.649697 | 0.037753 |
| LOC100289632 | 10179 | 2.649012 | 0.037813 |
| PLCE1 | 13984 | 2.648844 | 0.037814 |
| LGI4 | 9652 | 2.648163 | 0.037863 |
| FCAMR | 6469 | 2.648101 | 0.037863 |
| PGBD4 | 13729 | 2.647431 | 0.037921 |
| CAST | 3252 | 2.645134 | 0.038162 |
| POLR2J4 | 14179 | 2.644896 | 0.038172 |
| CES7 | 3837 | 2.644448 | 0.038182 |
| ADAMTSL4 | 586 | 2.644374 | 0.038182 |
| NKAIN2 | 12348 | 2.644342 | 0.038182 |
| OTUD6A | 13244 | 2.643455 | 0.038265 |
| ZNF579 | 20382 | 2.642032 | 0.038409 |
| SH3BGRL2 | 16439 | 2.641383 | 0.038465 |
| ANKRD56 | 1023 | 2.640652 | 0.038531 |
| BAALC | 1672 | 2.640329 | 0.03855 |
| OR6C76 | 13123 | 2.640114 | 0.038558 |
| SORCS1 | 17153 | 2.639619 | 0.038597 |
| SPRR2E | 17327 | 2.639074 | 0.038641 |
| ARHGEF3 | 1284 | 2.637921 | 0.038736 |
| CEACAM20 | 3757 | 2.63791 | 0.038736 |
| PCSK9 | 13544 | 2.637693 | 0.038736 |
| PPAP2B | 14249 | 2.637632 | 0.038736 |
| CENPL | 3798 | 2.636836 | 0.03881 |
| OR1N1 | 12897 | 2.636586 | 0.038821 |
| SLC7A4 | 16902 | 2.635653 | 0.0389 |
| MTND4P12 | 11840 | 2.635589 | 0.0389 |
| SLC19A3 | 16627 | 2.635109 | 0.038938 |
| CHIA | 3909 | 2.633706 | 0.039081 |
| ACOT11 | 454 | 2.632519 | 0.039201 |
| ITGA8 | 8745 | 2.631847 | 0.039261 |
| PTGIR | 14720 | 2.630994 | 0.039342 |
| PRIMA1 | 14459 | 2.629293 | 0.039521 |
| MACF1 | 10974 | 2.628819 | 0.039559 |
| CST3 | 4558 | 2.628326 | 0.039582 |
| ZDHHC16 | 19967 | 2.628317 | 0.039582 |
| KIAA2018 | 9143 | 2.628055 | 0.039595 |
| ZNF461 | 20291 | 2.627387 | 0.039655 |
| FBXO48 | 6453 | 2.62682 | 0.039698 |
| NAT8L | 12083 | 2.626588 | 0.039698 |
| KIAA1644 | 9121 | 2.626566 | 0.039698 |
| TSPAN8 | 18938 | 2.626281 | 0.039698 |
| RP11-65D13.1 | 15635 | 2.626024 | 0.039698 |
| PYROXD2 | 14841 | 2.626018 | 0.039698 |
| AC021850.1 | 234 | 2.625952 | 0.039698 |
| EPN3 | 5842 | 2.624999 | 0.039792 |
| FAM124A | 6090 | 2.624502 | 0.039809 |
| HSFX1 | 8243 | 2.624472 | 0.039809 |
| KCNG3 | 8890 | 2.6244 | 0.039809 |
| CLU | 4139 | 2.623401 | 0.039908 |
| NRIP1 | 12569 | 2.621922 | 0.040064 |
| LOC100131704 | 9976 | 2.621697 | 0.040073 |
| TAT | 17830 | 2.620208 | 0.04023 |
| CLIP2 | 4103 | 2.619733 | 0.040268 |
| SLC9A3R1 | 16917 | 2.617715 | 0.040489 |
| SPDEF | 17255 | 2.615232 | 0.040767 |
| PAQR8 | 13375 | 2.614843 | 0.040795 |
| DCHS2 | 4897 | 2.61466 | 0.040799 |
| PCSK1 | 13537 | 2.614398 | 0.040812 |
| PRKACG | 14467 | 2.613008 | 0.04096 |
| MOV10L1 | 11600 | 2.611979 | 0.041066 |
| PHGDH | 13786 | 2.611825 | 0.041066 |
| SDSL | 16176 | 2.611668 | 0.041067 |
| AANAT | 24 | 2.608505 | 0.04143 |
| SENP2 | 16242 | 2.608217 | 0.041444 |
| APLN | 1163 | 2.608091 | 0.041444 |
| LOC100133337 | 10037 | 2.607552 | 0.041491 |
| GJB6 | 7178 | 2.607325 | 0.0415 |
| LOC653051 | 10572 | 2.606999 | 0.041521 |
| CYB561 | 4728 | 2.606562 | 0.041556 |
| CACNA1S | 3111 | 2.604091 | 0.041827 |
| FABP1 | 6015 | 2.604035 | 0.041827 |
| CCNB2 | 3483 | 2.603668 | 0.041853 |
| F2R | 6002 | 2.602397 | 0.041964 |
| CCDC73 | 3415 | 2.602349 | 0.041964 |
| DES | 5057 | 2.602305 | 0.041964 |
| LDLR | 9598 | 2.601797 | 0.042 |
| OAF | 12722 | 2.601716 | 0.042 |
| CREB3L1 | 4427 | 2.600604 | 0.042118 |
| DYRK1B | 5496 | 2.600354 | 0.04213 |
| RPL29P2 | 15748 | 2.599517 | 0.042183 |
| TUBA3C | 19049 | 2.599355 | 0.042183 |
| KCNJ4 | 8915 | 2.599322 | 0.042183 |
| LOC100132147 | 9993 | 2.599319 | 0.042183 |
| GABRG1 | 6924 | 2.599099 | 0.042183 |
| BBC3 | 1724 | 2.598881 | 0.042183 |
| PC | 13437 | 2.598868 | 0.042183 |
| ISLR | 8721 | 2.597352 | 0.042351 |
| SCARNA17 | 16076 | 2.594569 | 0.042677 |
| TUBG1 | 19067 | 2.594147 | 0.042711 |
| MEIS2 | 11313 | 2.592875 | 0.042851 |
| ACTL7A | 506 | 2.591729 | 0.042975 |
| GDA | 7065 | 2.590804 | 0.043072 |
| BECN1 | 1790 | 2.590436 | 0.043099 |
| NGB | 12302 | 2.590031 | 0.043128 |
| SCAI | 16058 | 2.589907 | 0.043128 |
| C8orf73 | 2998 | 2.589314 | 0.043165 |
| PITPNB | 13897 | 2.589307 | 0.043165 |
| CRB3 | 4421 | 2.58894 | 0.043188 |
| SLC1A2 | 16629 | 2.588609 | 0.043188 |
| AC011427.1 | 196 | 2.588475 | 0.043188 |
| SEMG1 | 16239 | 2.588433 | 0.043188 |
| CHRM3 | 3942 | 2.588376 | 0.043188 |
| SUPT16H | 17639 | 2.587683 | 0.043257 |
| LOC100132644 | 10012 | 2.586765 | 0.043353 |
| AURKB | 1616 | 2.58615 | 0.043407 |
| RORC | 15517 | 2.586042 | 0.043407 |
| DLEU7 | 5172 | 2.58582 | 0.043416 |
| FOLR1 | 6722 | 2.585532 | 0.043433 |
| NOTCH2 | 12462 | 2.585278 | 0.043446 |
| NOVA1 | 12469 | 2.5846 | 0.043513 |
| OR5A1 | 13063 | 2.583474 | 0.043637 |
| UQCRHP2 | 19331 | 2.582932 | 0.043686 |
| KRTAP4-4 | 9443 | 2.582692 | 0.043698 |
| CMTM4 | 4156 | 2.582345 | 0.043723 |
| TSGA10 | 18900 | 2.581385 | 0.043826 |
| PHF15 | 13771 | 2.581106 | 0.043829 |
| AC024940.3 | 252 | 2.58106 | 0.043829 |
| KAT2B | 8845 | 2.578483 | 0.044097 |
| C3orf18 | 2736 | 2.578447 | 0.044097 |
| DCK | 4899 | 2.578386 | 0.044097 |
| WDTC1 | 19681 | 2.578365 | 0.044097 |
| RTL1 | 15908 | 2.577708 | 0.044161 |
| P2RX6 | 13275 | 2.577172 | 0.044211 |
| ZNF701 | 20477 | 2.576644 | 0.044248 |
| NOL10 | 12435 | 2.576584 | 0.044248 |
| FAM59B | 6280 | 2.576366 | 0.044257 |
| SLAMF7 | 16566 | 2.576029 | 0.044276 |
| GGN | 7118 | 2.575922 | 0.044276 |
| ZNF503 | 20317 | 2.575575 | 0.044301 |
| BAIAP2L2 | 1694 | 2.575144 | 0.044338 |
| ATIC | 1493 | 2.573943 | 0.044473 |
| LMBRD1 | 9832 | 2.573731 | 0.044481 |
| RETN | 15221 | 2.573119 | 0.044535 |
| NR2E1 | 12542 | 2.573016 | 0.044535 |
| AC010980.2 | 193 | 2.572654 | 0.044562 |
| KLHL25 | 9256 | 2.572053 | 0.044621 |
| MYO10 | 11960 | 2.57174 | 0.044628 |
| AC020663.1 | 230 | 2.571702 | 0.044628 |
| MFSD9 | 11386 | 2.571108 | 0.044685 |
| TCTE3 | 17961 | 2.56997 | 0.044813 |
| OTOF | 13230 | 2.569821 | 0.044813 |
| NAT14 | 12076 | 2.569355 | 0.044855 |
| AC074286.1 | 286 | 2.568999 | 0.044866 |
| PLXNA2 | 14068 | 2.568972 | 0.044866 |
| KCNA5 | 8867 | 2.567838 | 0.044994 |
| RNFT2 | 15487 | 2.567484 | 0.045021 |
| TMEM81 | 18467 | 2.567212 | 0.045037 |
| C20orf166 | 2584 | 2.5668 | 0.045071 |
| SLC2A13 | 16744 | 2.56656 | 0.045083 |
| UQCRFS1 | 19328 | 2.564407 | 0.045344 |
| SERPINC1 | 16313 | 2.563485 | 0.045446 |
| ENDOD1 | 5767 | 2.563329 | 0.045447 |
| MKLN1 | 11480 | 2.563111 | 0.045456 |
| LINC00479 | 9787 | 2.561987 | 0.045584 |
| PSMB3 | 14643 | 2.561261 | 0.045657 |
| LOC100133660 | 10041 | 2.561139 | 0.045657 |
| RABIF | 14946 | 2.560797 | 0.045682 |
| CT45A4 | 4574 | 2.560185 | 0.045744 |
| PSD4 | 14615 | 2.559792 | 0.045765 |
| RILPL1 | 15345 | 2.559602 | 0.045765 |
| KIAA0368 | 9037 | 2.559584 | 0.045765 |
| C10orf76 | 2040 | 2.559316 | 0.045781 |
| RINT1 | 15358 | 2.559132 | 0.045786 |
| PGAM4 | 13721 | 2.558303 | 0.045875 |
| AK3L1 | 753 | 2.556794 | 0.046055 |
| ZNF157 | 20101 | 2.556466 | 0.046079 |
| DLC1 | 5167 | 2.555509 | 0.046187 |
| PAPPA | 13366 | 2.555088 | 0.046223 |
| HGC6.3 | 7889 | 2.554841 | 0.046236 |
| LY6G5C | 10920 | 2.554668 | 0.04624 |
| DYRK3 | 5498 | 2.554345 | 0.046263 |
| TMEM72 | 18462 | 2.553835 | 0.046312 |
| ELL3 | 5715 | 2.552774 | 0.046421 |
| EIF5B | 5696 | 2.552718 | 0.046421 |
| RINL | 15357 | 2.552214 | 0.046469 |
| RFX1 | 15250 | 2.550694 | 0.046653 |
| ATP2B4 | 1529 | 2.55032 | 0.046683 |
| C2orf57 | 2700 | 2.550103 | 0.046692 |
| SCXB | 16141 | 2.549539 | 0.046735 |
| C10orf27 | 2024 | 2.549495 | 0.046735 |
| NEUROD2 | 12257 | 2.549021 | 0.04676 |
| LOC100128364 | 9876 | 2.549014 | 0.04676 |
| FAM27L | 6235 | 2.548485 | 0.046811 |
| CASKIN1 | 3233 | 2.548116 | 0.046841 |
| PMVK | 14099 | 2.547237 | 0.04694 |
| MLL3 | 11500 | 2.546026 | 0.047083 |
| C9orf129 | 3018 | 2.545784 | 0.047096 |
| ABCA3 | 42 | 2.545523 | 0.047112 |
| FLJ38723 | 6655 | 2.545187 | 0.047137 |
| ZNF2 | 20124 | 2.543695 | 0.047319 |
| NDUFS5P7 | 12200 | 2.543028 | 0.04739 |
| NT5E | 12615 | 2.54224 | 0.047477 |
| RELN | 15204 | 2.541999 | 0.04749 |
| LOC730167 | 10687 | 2.541651 | 0.047518 |
| CHRND | 3958 | 2.541501 | 0.047518 |
| PRODH | 14519 | 2.540351 | 0.047655 |
| BCL6 | 1769 | 2.540031 | 0.047679 |
| SCAF1 | 16056 | 2.539683 | 0.047706 |
| C14orf23 | 2213 | 2.538974 | 0.047765 |
| PALM | 13344 | 2.53896 | 0.047765 |
| MXD1 | 11895 | 2.538317 | 0.047832 |
| FREM2 | 6786 | 2.538181 | 0.047832 |
| COL8A1 | 4275 | 2.536596 | 0.048029 |
| FOXP4 | 6769 | 2.536135 | 0.048072 |
| TSPAN11 | 18920 | 2.535993 | 0.048072 |
| FLJ38668 | 6654 | 2.535474 | 0.048123 |
| KLK3 | 9284 | 2.534034 | 0.048301 |
| EFCAB1 | 5578 | 2.533603 | 0.04834 |
| SCN10A | 16105 | 2.532255 | 0.048507 |
| C1orf38 | 2524 | 2.531804 | 0.048549 |
| TTLL8 | 19016 | 2.530922 | 0.048651 |
| LMO4 | 9844 | 2.530737 | 0.048656 |
| POPDC3 | 14215 | 2.530412 | 0.048681 |
| GPT | 7515 | 2.530127 | 0.048701 |
| ACTL9 | 509 | 2.529785 | 0.048728 |
| SMAD5OS | 16970 | 2.529082 | 0.048806 |
| CABLES1 | 3093 | 2.528067 | 0.048927 |
| SIK1 | 16516 | 2.527015 | 0.049053 |
| CCDC108 | 3310 | 2.526192 | 0.049148 |
| RIT1 | 15367 | 2.52567 | 0.049201 |
| UTP14C | 19413 | 2.525244 | 0.049209 |
| C15orf26 | 2237 | 2.525233 | 0.049209 |
| C1orf61 | 2535 | 2.52518 | 0.049209 |
| NF1 | 12265 | 2.524936 | 0.049222 |
| PIK3CD | 13861 | 2.523797 | 0.049362 |
| APOL1 | 1190 | 2.523229 | 0.049421 |
| C2orf50 | 2694 | 2.522561 | 0.049478 |
| NUCB1 | 12640 | 2.522537 | 0.049478 |
| LOC124685 | 10246 | 2.521964 | 0.049517 |
| ATP13A4 | 1513 | 2.521717 | 0.049517 |
| SLITRK5 | 16953 | 2.521709 | 0.049517 |
| IHH | 8464 | 2.521678 | 0.049517 |
| ATN1 | 1498 | 2.518678 | 0.049915 |
| MCHR1 | 11225 | 2.518575 | 0.049915 |
| MRPL12 | 11659 | 2.518165 | 0.049953 |
